# Design, Synthesis and Antiparasitic Evaluation of Click Phospholipids

**DOI:** 10.3390/molecules26144204

**Published:** 2021-07-10

**Authors:** George E. Magoulas, Pantelis Afroudakis, Kalliopi Georgikopoulou, Marina Roussaki, Chiara Borsari, Theano Fotopoulou, Nuno Santarem, Emile Barrias, Paloma Tejera Nevado, Julia Hachenberg, Eugenia Bifeld, Bernhard Ellinger, Maria Kuzikov, Irini Fragiadaki, Effie Scoulica, Joachim Clos, Sheraz Gul, Maria Paola Costi, Wanderley de Souza, Kyriakos C. Prousis, Anabela Cordeiro da Silva, Theodora Calogeropoulou

**Affiliations:** 1 National Hellenic Research Foundation, Institute of Chemical Biology, 11653 Athens, Greece; gmagoulas@eie.gr (G.E.M.); pafroudakischem@yahoo.gr (P.A.); k.georgikopoulou@access-one.gr (K.G.); mroussaki@eie.gr (M.R.); tfotop@eie.gr (T.F.); kyrprous@eie.gr (K.C.P.); 2 Department of Biomedicine, University of Basel, 4058 Basel, Switzerland; chiara.borsari@unibas.ch; 3 i3S–Instituto de Investigação e Inovação em Saúde, Universidade do Porto, 4200-135 Porto, Portugal; santarem@ibmc.up.pt (N.S.); cordeiro@ibmc.up.pt (A.C.d.S.); 4 Parasite Disease Group, IBMC-Instituto de Biologia Molecular e Celular, 4150-180 Porto, Portugal; 5 Instituto Nacional de Ciência e Tecnologia em Biologia Estrutural e Bioimagens, Universidade Federal do Rio de Janeiro, Rio de Janeiro 21941-902, Brazil; emilebarrias@gmail.com (E.B.); wsouza@biof.ufrj.br (W.d.S.); 6 Instituto de Biofísica Carlos Chagas Filho, Universidade Federal do Rio de Janeiro, Ilha do Fundão, Rio Janeiro 21941-902, Brazil; 7 Bernhard Nocht Institute for Tropical Medicine, 20359 Hamburg, Germany; paloma.tejera.nevado@gu.se (P.T.N.); Julia.Hachenberg@amedes-group.com (J.H.); bifeld@bnitm.de (E.B.); clos@bni-hamburg.de (J.C.); 8 Fraunhofer Institute for Translational Medicine and Pharmacology ITMP, 22525 Hamburg, Germany; Bernhard.Ellinger@itmp.fraunhofer.de (B.E.); Maria.Kuzikov@itmp.fraunhofer.de (M.K.); Sheraz.Gul@itmp.fraunhofer.de (S.G.); 9 Fraunhofer Cluster of Excellence for Immune-Mediated Diseases CIMD, 22525 Hamburg, Germany; 10 Department of Clinical Microbiology and Microbial Pathogenesis, Faculty of Medicine, University of Crete, 70013 Heraklion, Greece; fragiada@med.uoc.gr (I.F.); e.scoulica@uoc.gr (E.S.); 11 Department of Pharmacy, Università degli Studi di Modena e Reggio Emilia, 41125 Modena, Italy; costimp@unimore.it; 12 Departmento de Ciências Biológicas, Faculdade de Farmácia, Universidade do Porto, 4099-002 Porto, Portugal

**Keywords:** ether phospholipids, heterocyclic rings, antiparasitic activity, *Leishmania* *infantum*, *Leishmania donovani*, *Trypanosoma brucei*, *Trypanosoma cruzi*

## Abstract

A library of seventeen novel ether phospholipid analogues, containing 5-membered heterocyclic rings (1,2,3-triazolyl, isoxazolyl, 1,3,4-oxadiazolyl and 1,2,4-oxadiazolyl) in the lipid portion were designed and synthesized aiming to identify optimised miltefosine analogues. The compounds were evaluated for their in vitro antiparasitic activity against *Leishmania infantum* and *Leishmania donovani* intracellular amastigotes, against *Trypanosoma brucei brucei* and against different developmental stages of *Trypanosoma cruzi*. The nature of the substituents of the heterocyclic ring (tail) and the oligomethylene spacer between the head group and the heterocyclic ring was found to affect the activity and toxicity of these compounds leading to a significantly improved understanding of their structure–activity relationships. The early ADMET profile of the new derivatives did not reveal major liabilities for the potent compounds. The 1,2,3-triazole derivative **27** substituted by a decyl tail, an undecyl spacer and a choline head group exhibited broad spectrum antiparasitic activity. It possessed low micromolar activity against the intracellular amastigotes of two *L. infantum* strains and *T. cruzi Y* strain epimastigotes, intracellular amastigotes and trypomastigotes, while its cytotoxicity concentration (CC_50_) against THP-1 macrophages ranged between 50 and 100 μM. Altogether, our work paves the way for the development of improved ether phospholipid derivatives to control neglected tropical diseases.

## 1. Introduction

Neglected tropical diseases (NTDs) are a group of infections primarily affecting people in developing regions worldwide, mainly in Africa, Asia and Latin America, due to the poor sanitation, lack of potable water and limited or no access to health care [1]. According to the WHO, NTDs such as leishmaniasis (*Leishmania* sp.), Chagas disease (*Trypanosoma cruzi*) and human African trypanosomiasis (HAT) or sleeping sickness as it is commonly called (*Trypanosoma brucei*) affect more than one billion people in 149 countries [2]. The new road map, launched by WHO, aiming to end the suffering caused by NTDs by the end of 2030, creates grounds for optimism regarding the fight against these diseases [3].

Leishmaniasis is a vector-borne disease caused by the protozoan *Leishmania* parasites, which are transmitted by the bite of infected female phlebotomine sandflies [4,5]. Leishmaniasis is endemic in areas of the tropics, subtropics and Southern Europe, whereas 900,000–1.3 million new cases and 20,000–30,000 deaths are estimated annually. The main forms of the disease are visceral (VL, also known as kala-azar), cutaneous (CL, the most common) and mucocutaneous. VL is the most severe form of Leishmaniasis, caused by *Leishmania infantum* and *Leishmania donovani*. Undoubtedly, it represents an emergent threat with high morbidity and mortality rates, since VL is fatal if left untreated in over 95% of cases. Unfortunately, the standard treatment of Leishmaniasis depends on poorly tolerated drugs with high toxicity. In addition, a major problem encountered in clinical practice is the increased chemoresistance of the parasite [6].

Pentavalent antimonials (sodium stibogluconate and meglumine antimonate) remain the first-line treatment for both VL and CL. However, they exhibit poor oral absorption and therefore they are administered intravenously or intramuscularly [7]. An improvement towards the treatment of Leishmaniasis has been the development of liposomal formulations of amphotericin B [8,9], which are more effective and less toxic. However, the high cost constitutes a stumbling block for people in developing countries. Another antileishmanial drug, pentamidine, currently used as a first-line treatment for CL caused by *Leishmania guyanensis*, is administered intravenously with severe side effects such as hypoglycaemia, myocarditis or hypotension [10,11].

Miltefosine (hexadecylphosphocholine) is an alkylphosphocholine analogue initially developed as an anticancer drug, which was later shown to exhibit antileishmanial activity. Miltefosine is currently the only oral drug available for the treatment of VL and CL. It has a long half-life (100–200 h) in humans and a low therapeutic index. It reaches steady-state concentrations in the plasma only a few days before the end of treatment, when viable parasites may still be present. Its major downside is that it induces teratogenesis in animals and, thus, cannot be administered to pregnant women [12,13]. Furthermore, miltefosine can result in severe gastrointestinal side effects such as vomiting, diarrhoea, nausea or loss of appetite [14].

Trypanosomiasis is a serious parasitic disease transmitted by insects caused by protozoa of the genus *Trypanosoma*. There are two forms of the disease: Chagas disease caused by *T. cruzi* and human African trypanosomiasis (HAT) caused by *Trypanosoma brucei gambiense* or *Trypanosoma brucei rhodesiense*. Chagas disease is encountered mainly in South America with more than 25 million people being at risk and approximately 10,000 deaths annually [15]. Treatment of the disease is challenging since the majority of symptoms are elusive. Often the patient’s health deteriorates and death from sudden cardiac arrest is an outcome. There are only two drugs currently available for the treatment of Chagas disease, namely, nifurtimox and benznidazole. These drugs are used for both the acute and chronic stages of the disease, albeit with low cure rates in particular during the chronic phase. In addition, they are associated with serious side effects especially in elderly patients, with dermatitis due to hypersensitivity and digestive intolerance being the most frequent [16,17,18].

HAT is endemic in sub-Saharan Africa where over 70 million people are at risk [19]. The available treatments of HAT are few depending on the stage of the disease, with limitations mainly due to toxicity and route of administration. Pentamidine and suramin are the treatment of choice for the haemolymphatic early stage of HAT. Melarsoprol, despite its high toxicity, is widely used against the second meningo-encephalitic stage of HAT caused by *T. b. rhodesiense* during which, the parasite invades the central nervous system (CNS) [20,21]. At the same time, eflornithine is the drug of choice for the CNS stage of HAT caused by *T. b. gambiense*.

Undoubtedly, the existing medications for the treatment of leishmaniasis and trypanosomiasιs suffer from a variety of problems including serious side-effects, lengthy course of treatment, high cost and development of parasite resistance.

In the context of our studies towards derivatives of miltefosine with improved antileishmanial and antitrypanosomal activity, [22,23,24,25,26,27,28,29,30] it became evident that introduction of carbocyclic rings in the lipid portion of alkylphosphocholines resulted in increased potency against *L. infantum* intracellular amastigotes accompanied by reduced toxicity against THP-1 macrophages. Prompted by the well documented important biological and pharmacological properties of five-membered heterocyclic rings [31], we set out to investigate their effect on alkylphosphocholines. In particular, the 1,2,3-triazole ring, being the most explored amide bond bioisostere [32], has been extensively employed in medicinal chemistry, resulting in compounds with antimicrobial, antitumor, antitubercular, anticonvulsant, antidepressant, antimalarial and anti-inflammatory activities [33]. Furthermore, the isoxazole [34], the 1,2,4- [35] and 1,3,4-oxadiazoles [36] have been widely used as pharmacophores due to their low toxicity and the wide range of bioactivities of the respective derivatives including antibacterial, antiviral, anticonvulsant and antitrypanosomal activity.

**Figure 1:**
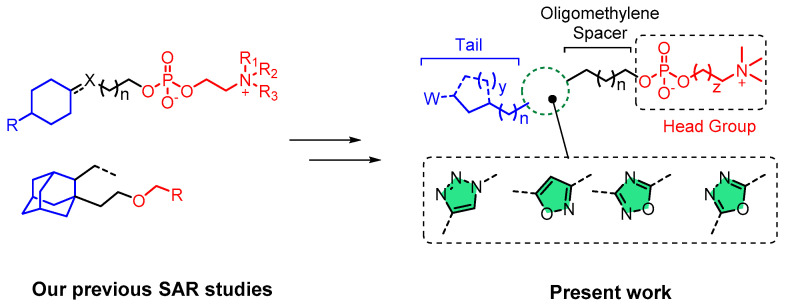
General structures of the compounds synthesized in the past and in the present work.

In the context of the present work, a series of 17 novel ether phospholipid derivatives was synthesized and structure–activity relationships were obtained. A systematic chemical investigation was performed considering the following modifications: (a) conformational restriction by incorporating 5-membered heteroaromatic rings in the lipid portion namely, 1,2,3-triazole, 1,2,4-oxadiazole, 1,3,4-oxadiazole and 3,5-disubstituted isoxazole, (b) variable length of the oligomethylene spacer between the heterocyclic ring and the phosphate head group, (c) replacement of choline by homocholine in the phosphate polar head-group and (d) attachment of alkyl, cycloalkyl, alkylcarbamate and carboxyalkyl groups as substituents of the heterocyclic ring (Figure 1).

## 2. Results and Discussion

### 2.1. Chemistry

The general synthetic procedure for the synthesis of the new ether phospholipids **25–36**, **53**, **54**, **65** and **66** involves the phosphorylation of the appropriate alcohols **17**–**24, 40**, **51**, **52**, **63** and **64**, respectively with phosphorus oxychloride in the presence of triethylamine in THF to afford the corresponding phosphoric acid derivatives. These in turn are treated with pyridine to form the pyridinium salts, which are coupled with choline or homocholine tosylate in the presence of 1-(mesitylenesulfonyl)-3-nitro-1,2,4-triazolide (MSNT) or 2,4,6-tri-isopropylbenzenesulfonyl chloride (TPSCl) as a condensing agent to afford the phospholipid derivatives of the present study.

**Scheme 1:**
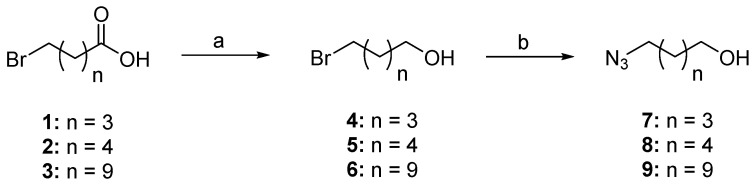
Synthesis of azides **7**–**9**. Reagents and conditions: (**a**) BH_3_∙Me_2_S, THF, rt, 1 h; (**b**) NaN_3_, DMF, 40 °C, 2 days.

**Scheme 2:**
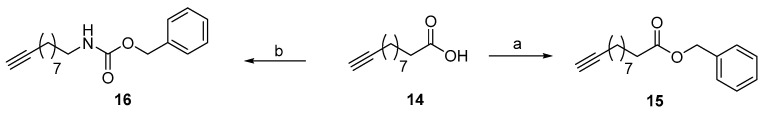
Synthetic route to alkynes **17**, **18**, **21** and **22**. Reagents and conditions: (**a**) benzyl bromide, Cs_2_CO_3_, DMF, rt, 16 h. (**b**) (1) DPPA, Et_3_N, benzene, 80 °C, 1 h; (2) benzyl alcohol, 80 °C, 16 h.

**Scheme 3:**
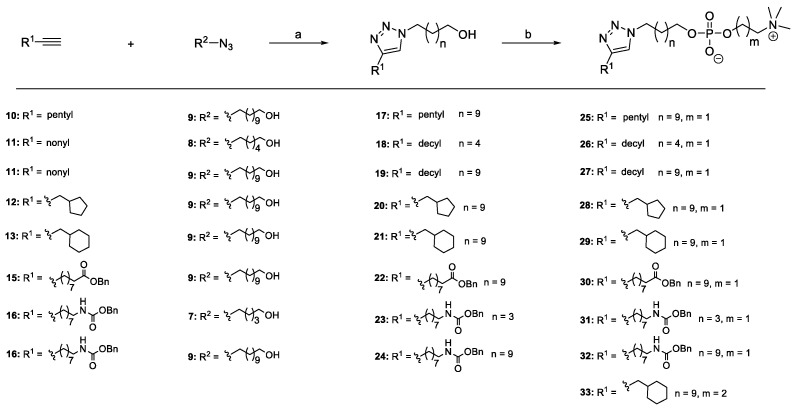
Synthesis of 1,2,3-triazolyl-substituted alcohols (**17**–**24**) and ether phospholipids (**25**–**33**). Reagents and conditions: (**a**) CuSO_4_, sodium ascorbate, ^t^BuOH/H_2_O (4:1), rt, 12 h. (**b**) (i) POCl_3_, Et_3_N and THF, −5 °C, 15 min then H_2_O, 30 min; (ii) pyridine, 45 °C, 2.5 h; (iii) pyridine, choline or homocholine tosylate, MSNT or TPSCl, 0 °C then rt, 48 h.

In particular, the synthesis of the 1,2,3-triazole-substituted alcohols **17**–**24** was affected through a click reaction between azides **7**–**9** and alkynes **10**–**16** and is described in Scheme 1, Scheme 2 and Scheme 3. Thus, commercially available ω-bromo carboxylic acids **1**–**3** were treated with BH_3_∙Me_2_S in THF to afford the corresponding bromoalcohols **4**–**6** in 95–100% yield [37], which were in turn reacted with NaN_3_ in DMF to give ω-azidoalcohols **7**–**9** in 92–97% yield. (Scheme 1).

The required alkynes were either commercially available (compounds **10**–**13**) or prepared as described in Scheme 2. Alkyne **15** was obtained by esterification of 10-undecynoic acid (**14**) with benzyl bromide in DMF using Cs_2_CO_3_ as a base in 93% yield. Alkyne **16** was synthesized through a Curtius rearrangement reaction from 10-undecynoic acid (**14**) using diphenylphosphoryl azide followed by the addition of benzyl alcohol in 66% yield [38].

Following the synthesis of the desired alkynes (**10**–**13**, **15** and **16**) and azides (**7**–**9**), alcohols **17**–**24** incorporating a 1,2,3-triazole ring were prepared through a copper catalysed Huisgen cycloaddition reaction in 51–80% yield (Scheme 3) [39]. The reaction was carried out in the presence of CuSO_4_∙5H_2_O and sodium ascorbate in ^t^BuOH/H_2_O (4:1). Finally, the desired ether phospholipids (**25**–**33**) were obtained according to the procedure described above (Scheme 3).

**Scheme 4:**
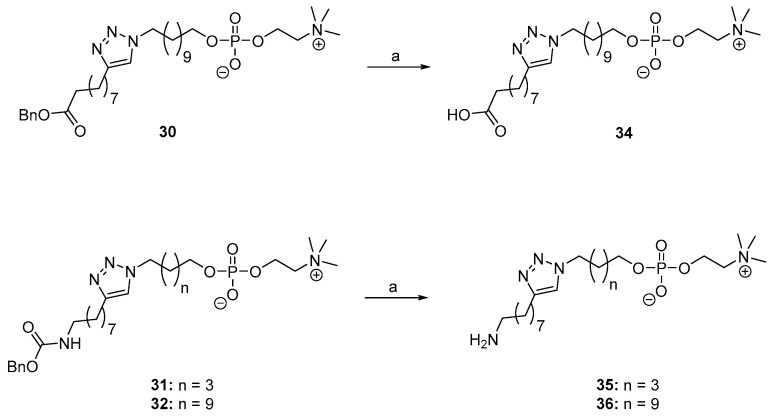
Synthesis of ether phospholipids **34**–**36** via catalytic hydrogenolysis. Reagents and conditions: (**a**) H_2_, 10% Pd/C, MeOH, rt, 12 h.

Subsequently, ether phospholipid **30** was subjected to palladium-catalysed hydrogenolysis to obtain carboxylic acid **34** in 95% yield, while, the treatment of phospholipids **31** and **32**, under the same reaction conditions, led to analogues **35** and **36** bearing a primary amino functionality in 90 and 97% yield respectively (Scheme 4).

**Scheme 5:**
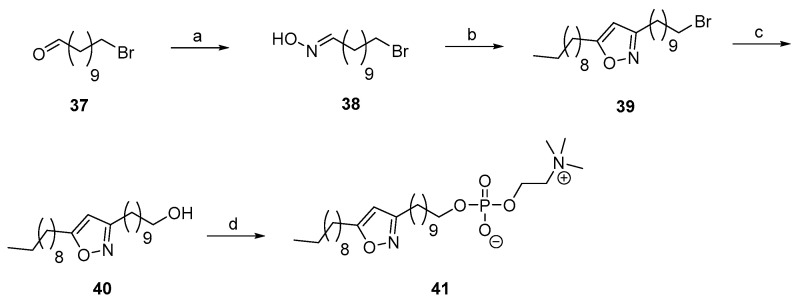
Synthesis of ether phospholipid **41**. Reagents and conditions: (**a**) NH_2_OH∙HCl, 1 M aq. NaOH and ^t^BuOH, rt, 1.25 h; (**b**) 1-dodecyne, Et_3_N, NaOCl and CH_2_Cl_2_, rt, 24 h; (**c**) K_2_CO_3_ and H_2_O/DMSO (2:1), 100 °C, 24 h; (**d**) (1) POCl_3_, Et_3_N and THF, −5 °C, 15 min then H_2_O, 30 min; (2) pyridine, 45 °C, 2.5 h; (3) pyridine, choline tosylate and MSNT, 0 °C then rt, 48 h.

The library of click phospholipids was further expanded with the synthesis of selected new derivatives in which the 1,2,3-triazole ring was replaced by isoxazole, 1,2,4- or 1,3,4-oxadiazole rings. The synthesis of phospholipid **41** incorporating an isoxazole ring is depicted in Scheme 5. Aldehyde **37,** which was obtained after PCC-mediated oxidation of 11-bromoundecanol (**6**) [40], was converted to the corresponding oxime **38** upon treatment with NH_2_OH∙HCl in ^t^BuOH:H_2_O (1:1). **38** was then subjected to a 1,3-dipolar nitrile oxide/alkyne cycloaddition reaction with commercially available 1-dodecyne in the presence of NaOCl to afford the isoxazole derivative **39**. Subsequently, bromide **39** was converted to the corresponding alcohol **40** in the presence of K_2_CO_3_ in H_2_O/DMSO (2:1), which was phosphorylated using POCl_3_ and further reacted with choline tosylate in the presence of MSNT as a condensing agent to obtain the desired phospholipid derivative **41** (Scheme 5).

**Scheme 6:**
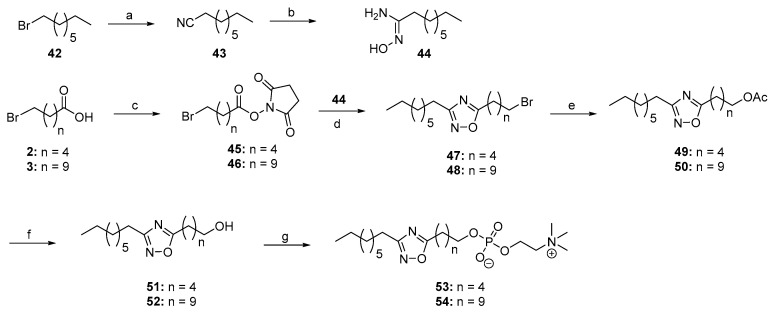
Synthesis of ether phospholipids **53** and **54**. Reagents and conditions: (**a**) NaCN and DMF, 90 °C, 2 days; (**b**) NH_2_-OH∙HCl, NaHCO_3_ and ^i^PrOH, 85 °C, 4 h; (**c**) NHS, DCC and THF, rt, 4 h; (**d**) PhMe and MW, 160 °C, 5 min; (**e**) CH_3_COOK and DMF, 80 °C, 3–4 h; (**f**) LiOH and THF/MeOH (1:3), rt, 2 h; (**g**) (1) POCl_3_, Et_3_N and THF, −5 °C, 15 min then H_2_O, 30 min; (2) pyridine, 45 °C, 2.5 h; (3) pyridine, choline tosylate and MSNT, 0 °C then rt, 48 h.

The preparation of phospholipids **53** and **54** bearing a 1,2,4-oxadiazole ring is described in Scheme 6. Nonanitrile (**43**) was obtained upon treatment of 1-bromooctane (**42**) with NaCN in DMF and was subsequently reacted with NH_2_-OH∙HCl in the presence of NaHCO_3_ to obtain amidoxime **44** in 59% yield. The required active esters **45** and **46** were synthesized from the commercially available ω-bromo acids **2** and **3**, respectively, after reaction with NHS and DCC in THF. Subsequently, the formation of the 1,2,4-oxadiazole-containing bromides **47** and **48** was accomplished in 52% and 64% yield, respectively, through the reaction of **44** with **45** or **46** under microwave irradiation. Thereafter, reaction of bromides **47** and **48** with CH_3_CO_2_K and hydrolysis of the obtained acetates **49** and **50** with LiOH afforded the required alcohols **51** and **52,** which were phosphorylated following the procedure described above to obtain phospholipids **53** and **54**, respectively (Scheme 6).

**Scheme 7:**
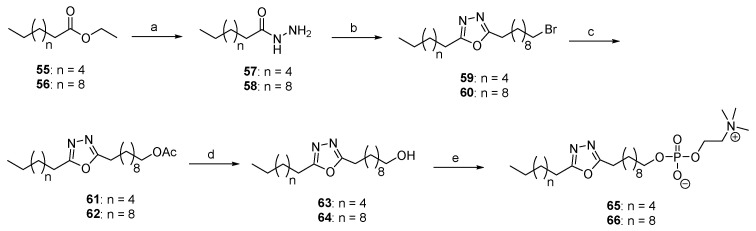
Synthesis of ether phospholipids **65** and **66**. Reagents and conditions: (**a**) NH_2_NH_2_∙H_2_O, EtOH and MW, 120 °C, 17 min (2 cycles); (**b**) **3**, POCl_3_, reflux, 2–4 h; (**c**) CH_3_COOK and DMF, 80 °C, 3 h; (**d**) 0.2 M aq. solution LiOH and THF/MeOH (1:3), rt, 1 h; (**e**) (1) POCl_3_, Et_3_N and THF, −5 °C, 15 min then H_2_O, 30 min; (2) pyridine, 45 °C, 2.5 h; (3) pyridine, choline tosylate and MSNT, 0 °C then rt, 48 h.

The synthesis of phospholipids **65** and **66** bearing a 1,3,4-oxadiazole ring is reported in Scheme 7. Commercially available ethyl octanoate (**55**) or ethyl dodecanoate (**56**) were converted to the corresponding hydrazides **57** and **58** under microwave irradiation using NH_2_NH_2_∙H_2_O in 33% and 54% yield, respectively, and were subsequently coupled with 11-bromoundecanoic acid (**3**) in the presence of POCl_3_ to furnish the 1,3,4-oxadiazole-substituted bromides **59** and **60** in 60% and 77% yield, respectively. These in turn were converted to the corresponding alcohols **63** and **64** following an analogous procedure as described above for alcohol **51**. Eventually, phospholipids **65** and **66** were obtained from the phosphorylation of alcohols **63** and **64** and reaction with choline tosylate in the presence of MSNT (Scheme 7).

### 2.2. Biological Evaluation

In vitro evaluation of antileishmanial activity: Initially, the antileishmanial activity of the 1,2,3-triazolyl-substituted phospholipids **25**–**29** was evaluated against the intracellular amastigotes of *L. infantum* strain MHOM/TN/80/LEM235 (see Table S1). Interestingly, the oligomethylene spacer between the heterocyclic ring and the phosphocholine head group and the aliphatic substituent on the 1,2,3-triazole ring (tail) influenced the activity. Thus, compound **25** bearing a pentyl tail and an undecyl spacer was less potent (IC_50_ = 9.33 ± 0.99 μΜ) against *L. infantum* than **26**, substituted by a decyl tail and a hexyl spacer (IC_50_ = 5.21 ± 0.95 μΜ), although the total number of carbons of the tail and spacer of the two compounds were identical. Furthermore, maintaining the decyl tail, the length of the spacer was increased to eleven carbons leading to a further improvement in the potency of compound **27** against *L. infantum* (IC_50_ = 1.54 ± 0.22 μΜ). Based on the above data, the undecyl spacer was kept and the aliphatic tail was replaced by cycloalkyl rings aiming to reduced compound flexibility. Thus, **28** bearing a cyclopentylmethylene tail possessed similar potency to **27** with IC_50_ = 1.5 ± 0.4 μΜ while increasing the ring size to a cyclohexyl group was beneficial to the antileishmanial activity of **29** (IC_50_ = 0.5 ± 0.2 μΜ) that was 13-fold more potent than miltefosine. Since it was observed that different *Leishmania* strains were associated with diverse susceptibility to the same compound [41,42], the effect of derivatives **25**–**29** was assessed against the intracellular amastigotes of a second *L. infantum* strain, namely MHOM/MA/67/ITMAP-263 (Table 1). However, this strain was not as sensitive to click phospholipids **25**–**29**. In particular, when tested at a single dose of 10 μΜ **28** was inactive while, **25**, **26**, **27** and **29** inhibited *L. infantum* amastigotes by 32%, 44%, 78% and 43%, respectively. Only **27** resulted in >50% inhibition at 10 μΜ and the IC_50_ value was determined to be 4.23 ± 0.94 μΜ, which was slightly inferior to that against *L. infantum* LEM235 amastigotes but similar to that of miltefosine. Thus, it was decided to proceed with the evaluation of the remaining new analogues only against *L. infantum* (MHOM/MA/67/ITMAP-263) amastigotes since it is more resistant to the click phospholipids (Table 1). Prompted by this initial SAR, additional 1,2,3-triazole containing phospholipids were synthesized by introducing polar head groups to the lipophilic tail. Thus, an ester (compound **30**), a carbamate (compounds **31** and **32**), a carboxy (compound **34**) and amino groups (compounds **35** and **36**) were introduced. An undecyl oligomethylene spacer was maintained for the ester and acid-substituted derivatives **30** and **34**. Two different spacers (the pentyl and undecyl groups) were studied for the carbamate and corresponding amino analogues. Unfortunately, the presence of polar groups as terminal substituents of the lipophilic tail abolished the activity against *L. infantum* (MHOM/MA/67/ITMAP-263) amastigotes while the toxicity against THP1 macrophages was not affected (see Table 1, compounds **30**–**36**). In addition, replacement of the choline head group by a homocholine in analogue **29** (compound **33**) did not improve the antileishmanial activity. This structural modification was based on previous reports that erufosine (ErPC3), which contains a homocholine head group, displays reduced bone marrow toxicity than miltefosine [43,44]. Subsequently, we investigated the effect of other five-membered heterocyclic rings on the antileishmanial activity. Thus, the 1,2,3-triazole moiety was replaced by isoxazole (**41**), 1,2,4-oxadiazole (**53**,**54**) and 1,3,4-oxadiazole (**65**,**66**) with concomitant variations of the length of the lipophilic tail and the oligomethylene spacer. Interestingly, the activity of the new compounds against *L. infantum* (MHOM/MA/67/ITMAP-263) amastigotes ranged between 92 and 100% inhibition at 10 µM. However, the high level of activity was not observed at a 1 μΜ concentration except for the 1,2,4-oxadiazole derivative **54** substituted by an octyl tail and a decyl oligomethylene spacer, which exhibited 67.1% inhibition at 1 µM and an IC_50_ value of 0.8 ± 0.18 μM. Thus, compound **54** was 4-fold more potent than miltefosine. A common characteristic of compounds **41**, **53**, **54**, **65** and **66** was the higher toxicity against THP1 macrophages, which ranged between 12.5 and 25 μM for **41**, **53** and **65** and 25–50 μM for **66**. Although the cytotoxicity of the potent derivative **54** was similar to that of miltefosine (CC_50_ > 10 μM), the corresponding selectivity index was 5.6-fold higher (SI = 13.6 and SI = 4.9, respectively).

**Figure 2:**
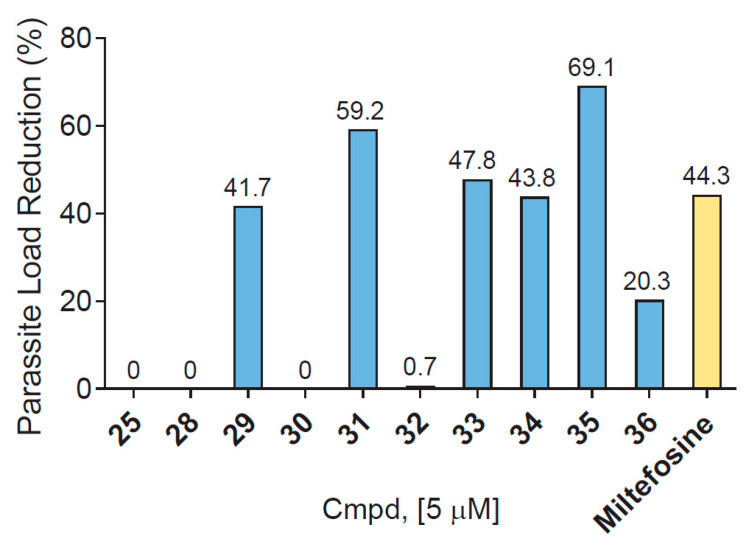
In vitro evaluation of activity against *L. donovani* infected bone marrow-derived macrophages.

To acquire a more comprehensive understanding of the range of activity of the new click phospholipids, the activity of the 1,2,3-triazolyl-substituted derivatives **25** and **28**–**36** were also screened at 5 μM concentration against *L. donovani* amastigotes in infected bone marrow-derived macrophages using Taqman qPCR of the *Leishmania* actin gene over the mouse actin gene (Figure 2). These derivatives exhibited higher activity than against *L. infantum* (MHOM/MA/67/ITMAP-263) amastigotes. Compound **29** bearing a cyclohexylmethyl tail reduced the parasite load by 41.7% similar to miltefosine (44.3%) and was also found to be active against *L. infantum* (MHOM/TN/80/LEM235) amastigotes. Replacing choline in **29** with homocholine in **33** did not reduce the percentage (%) of parasite load. Conversely, the length of the oligomethylene spacer in the two 1,2,3-triazolyl-carbamate derivatives **31** and **33** played a critical role in the activity against *L. donovani* amastigotes. **31** substituted by a pentyl spacer resulted in a 59.2% parasite load reduction at 5 μΜ, while the undecyl spacer abolished the activity (compound **32**). Furthermore, deprotection of the carbamate in **31** resulted in the amino derivative **35** with increased potency to a 69.1% parasite load reduction, corresponding to a 1.5-fold higher activity than miltefosine (Figure 2).

**Figure 3:**
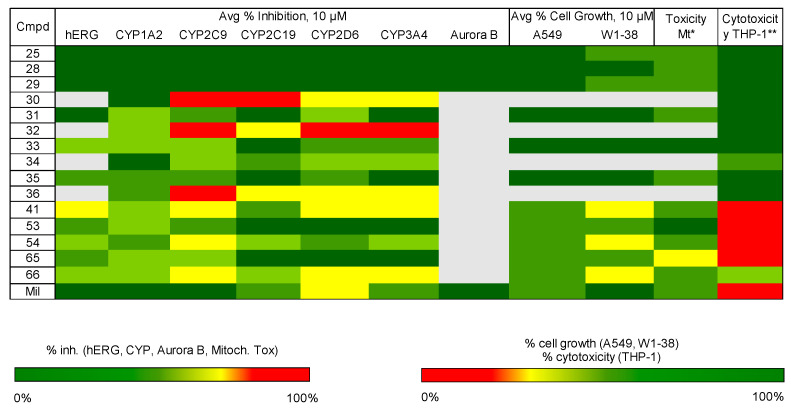
In vitro evaluation of toxicities (hERG, CYP1A2, CYP2C9, CYP2C19, CYP2D6, CYP3A4 and A549 and WI38 cytotoxicities) of compounds at 10 µM and miltefosine (10 µM or 1 µM). The compound with the best profile is that with dark green boxes in all assays. The grey colour corresponds to non-tested compounds. * Average % of mitochondrial toxicity 10 μM. ** Cytotoxicity THP-1 macrophages.

*Early ADMET profiling*: The early ADMET profiling of the new derivatives involved a group of assays widely used in drug development as part of the preclinical in vitro toxicity studies. In particular our panel included hERG inhibition, five cytochromes P450 (CYP1A2, CYP2C9, CYP2C19, CYP2D6 and CYP3A4), two cell-lines (A549, epithelial human lung adenocarcinoma, WI38 and foetal lung fibroblasts), Aurora B kinase and mitochondrial toxicity, in addition to the parasite relevant toxicity against THP1 macrophages. The compounds were tested at 10 μΜ, whilst the miltefosine control was tested at 10 μΜ or 1 μM. The toxicity of the new click phospholipids is depicted in Figure 3 in a traffic light format (from red through yellow to green for decreasing ADMET liabilities). The values are included in Table S2.

Ideally, for the perfect lead compound all results should be green. None of the compounds tested was associated with CYP1A2, or A549 liability. Only **41** was slightly toxic against hERG. Derivatives **25**, **28**, **29**, **31**, **33**, **34**, **35**, **53** and **65** had no liabilities, except for different levels of toxicity against THP1 macrophages. **30**, **32** and **36** exhibited toxicity against CYP2C9, CYP2C19, CYP2D6 and CYP3A4 while **41** and **66** were slightly toxic against CYP2C9, CYP2D6 and CYP3A4 and **54** against CYP2C9. Only **41**, **54** and **66** showed mild toxicity against W1-38 cells.

In vitro *evaluation of antitrypanosomal activity against T. b. brucei:* Most of the synthesized compounds were tested for their antitrypanosomal activity against the *T. b. brucei* L427 WT bloodstream form in a single dose assay at 10 μM using a modified resazurin-based assay. Pentamidine was used as an internal control for the fitness of the parasite (quality control of the assay). All compounds tested were associated with a minimum or no activity against *T. b. brucei* (see Table S3).

**Figure 4:**
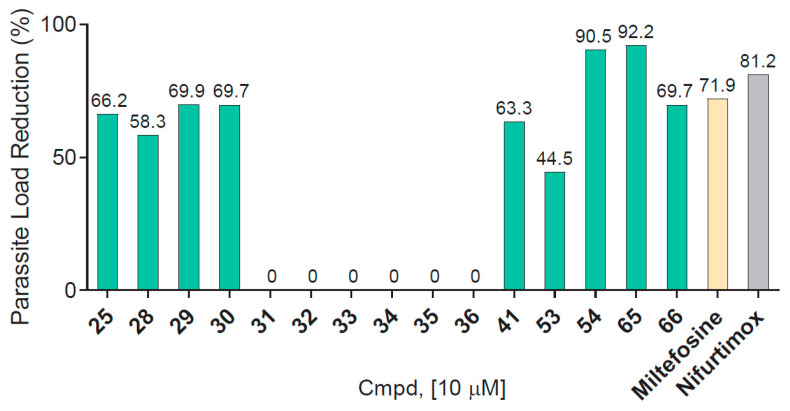
In vitro evaluation of activity against *T. cruzi* Y strain intracellular amastigotes.

In vitro *evaluation of antitrypanosomal activity against T. cruzi*: Selected compounds (**25**, **28**–**36**, **41**, **53**, **54**, **65** and **66**) were tested at 10 μM against *T. cruzi* Y strain intracellular amastigotes propagated through a HG39 cell-line (Figure 4). As a general observation among the 1,2,3-triazolyl-substituted phospholipids, the presence of a carbamate, acid or amino terminal group at the lipophilic tail abolished activity (**31**, **32** and **34**–**36).** Furthermore, replacement of the choline head group by homocholine in the cyclohexylmethyl derivative **29** was detrimental to activity against *T. cruzi* amastigotes, in particular **29** resulted in 69.7% parasite load reduction similar to miltefosine (71.9% parasite load reduction) while **33** was inactive. The isoxazole-substituted analogue **41** induced 63.3% parasite load reduction, while, for the two 1,2,4-oxadiazole-substituted derivatives **53** and **54**, the latter showed a higher antitrypanosomal potency (90.5% vs. 44.5% parasite load reduction). Finally, between the 1,3,4-oxadiazole derivatives **65** and **66** compound **65** bearing a shorter heptyl tail was very potent (92.2% parasite load reduction) in comparison to **66,** which was substituted by an undecyl tail. Interestingly, among compounds **41**, **53**, **54**, **65** and **66**, **53** with a pentyl spacer was the least potent highlighting that the activity was impacted by the oligomethylene spacer.

Subsequently, a more in-depth evaluation against all the parasite developmental stages of *T. cruzi* [45] was performed for a small number of 1,2,3-triazole-substituted derivatives (**25**, **27** and **29**) based on their antileishmanial activity and reduced toxicity against THP-1 derived macrophages. In particular, *T. cruzi* epimastigotes (Y strain) were obtained by axenic cultivation in the LIT medium, supplemented with 10% FBS (Cultilab) and the effect of compounds **25**, **27** and **29** was determined after 24 h, 48 h and 72 h treatment (Table 2). The most potent derivative was **27** substituted by a decyl tail, an undecyl spacer and a choline head group with IC_50_ values in the submicromolar range following 48 h or 72 h of treatment at 7.8 μΜ. Shortening the tail length to a pentyl group (**25**) resulted in a reduction of activity by about threefold after 72 h of treatment. Rigidification of the lipophilic tail through a cyclohexylmethyl substituent (**29**) led to a drop of activity. Interestingly, phospholipid **27** was also potent against *L. infantum* MHOM/TN/80/LEM235, *L. infantum* MHOM/MA/67/ITMAP-263 and *L. donovani* amastigotes.

**Figure 5:**
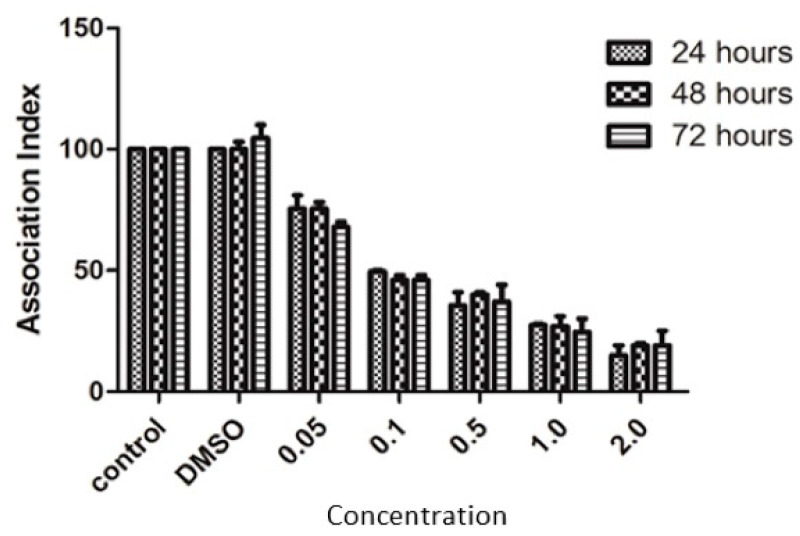
Treatment of peritoneal macrophages infected with intracellular amastigotes of *T. cruzi* with phospholipid **27**.

Click phospholipids **25**, **27** and **29** were also evaluated against *T. cruzi* amastigotes proliferating within peritoneal macrophages from Swiss mouse (CF1). A dose dependent inhibition was observed with phospholipid **27** with IC_50_ values of 0.1 μM at 24 h, 0.087 μΜ at 48 h and 0.057 μΜ at 72 h (Table 3 and Figure 5). Light microscopy observations of treated macrophages containing intracellular amastigotes revealed a gradual reduction in the number of amastigotes but no changes in the morphology of the host cell, which even remained attached to the substrate.

**Figure 6:**
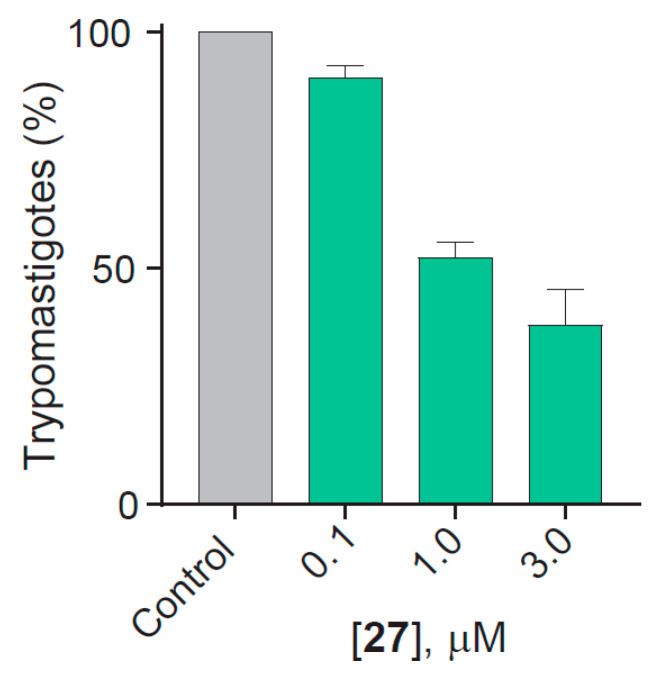
Evaluation of the cytotoxic effect on *T. cruzi* trypomastigotes with compound **27.** The evaluation was done after 24 h of treatment.

In order to determine if compounds **25**, **27** and **29** were able to lyse *T. cruzi* trypomastigotes, this developmental stage was treated for 24 h with various compound concentrations. Significant lysis of the parasites was observed only with the **27** treatment (LD_50_ = 1.02 μΜ, Figure 6), highlighting its broad spectrum antiparasitic profile.

**Figure 7:**
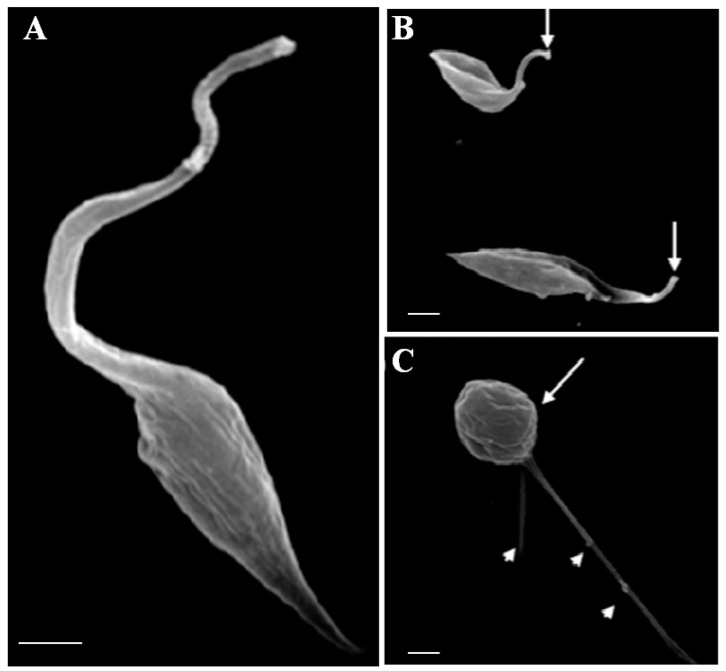
Scanning electron micrographs of *T. cruzi* epimastigotes treated with compound **27**. (**A**) Control after 72 h of growth. Integrity of untreated cells: elongated cell body and single tapering flagellum; (**B**) shortening of the flagellum when compared to the control (arrow); (**C**) multiple flagella (arrowheads) and misshapen cell body (arrow). Bars = 1 µM.

*Morphological analysis of epimastigotes and amastigotes treated with click phospholipid **27**:* Since compound **27** was the most potent against all the developmental stages of *T. cruzi*, scanning and transmission electron microscopy were employed to analyse the effects on epimastigotes and intracellular amastigotes. Treatment of *T. cruzi* epimastigotes with **27** for 72 h (100 nM) resulted in changes in their morphology, gradually losing the typical elongated form and acquiring a rounded shape (Figure 7B,C), confirming initial observations made by light microscopy (data not shown).

**Figure 8:**
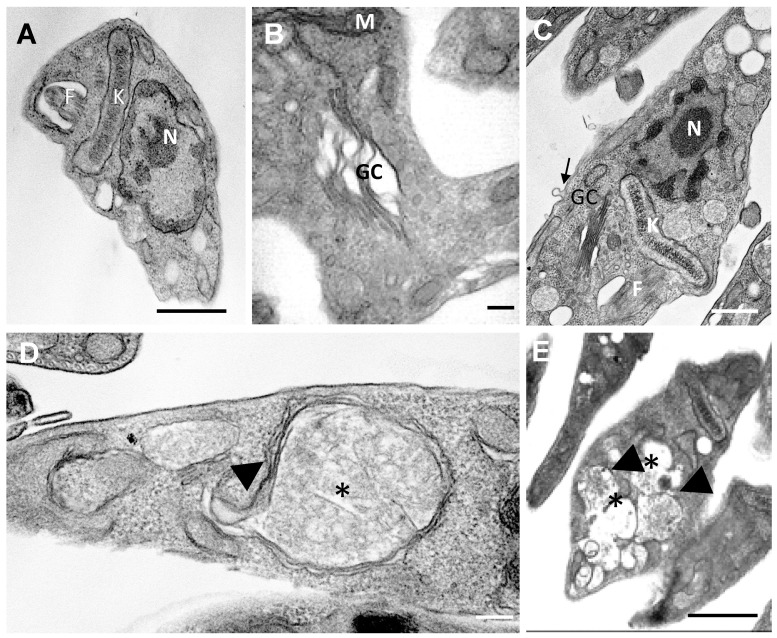
Transmission electron micrographs of *T. cruzi* epimastigotes treated with compound **27** (concentration = IC_50_ calculated in epimastigotes) for 48 h. (**A**) The untreated control shows the preserved organelles. (**B**–**E**) Epimastigotes treated with **27** and disorganization of the Golgi complex (**B** and **C**, GC), formation of autophagosome-like cytoplasmic vacuoles (**D** and **E**, asterisks) surrounded by a membrane similar to the endoplasmic reticulum (arrowheads) and detachment of the membrane plasma (B—arrow). N = nucleus; K = kinetoplast; GC = Golgi complex; F = flagellum. Bars = 0.5 µm.

Furthermore, observation of the untreated epimastigotes by transmission electron microscopy (TEM) showed cells with an elongated shape, a smooth cell surface and intact organelles (Figure 8A). Micrographs of epimastigotes treated with compound **27** (100 nM, for 72 h) revealed drastic alterations in the Golgi complex in particular disorganization of its cisternae, which had adopted dilated and empty appearances (Figure 8B). Mitochondrial swelling and membrane blebs on the parasite flagellum were also evident (Figure 8C,D).

**Figure 9:**
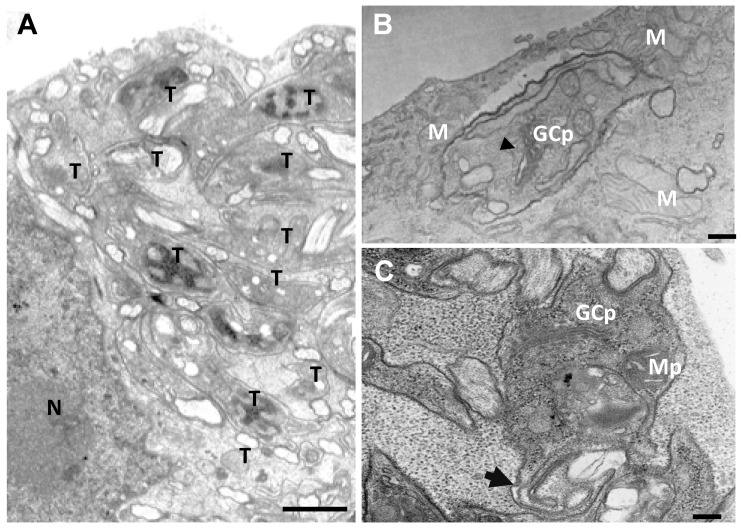
Transmission electron micrographs of *T. cruzi* intracellular amastigotes after 72 h of treatment with **27** (IC_50_ concentration). (**A**) The untreated control showing a host cell with intracellular trypomastigotes (T). Host cell and parasites do not show any changes. (**B**,**C**) Interactions between LLCM-K2 and *T. cruzi* treated with **27** show alterations in the Golgi complex (B–GC) and the formation of structures similar to autophagosomes (C-arrow). N = nucleus, T = trypomastigotes, GC = Golgi complex, M = mitochondria. Bar = 1 µm.

The ultrastructural analysis via TEM of T. cruzi intracellular amastigotes revealed, after 48 hours treatment with 100 nM of compound **27,** the presence of Golgi complex with mischaracterization of its lamellae (Figure 9B, GCp arrowhead) and formation of structures similar to autophagosomes (C-arrow). No ultrastructural changes were observed in the host cells.

**Figure 10:**
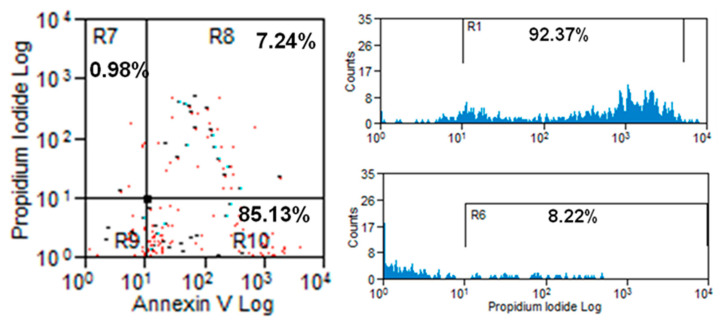
Flow cytometry of *T. cruzi* epimastigotes treated with 5 µM of compound **27** for 24 h labelled with annexin V-Alexa 488 nm and propidium iodide. Approximately 85% of epimastigotes are marked with annexin V, indicating an onset of the apoptotic process. Of these, more than 90% are also marked with propidium iodide, indicating a loss of integrity of the plasma membrane.

Flow cytometry studies in *T. cruzi* epimastigotes treated with **27**: Flow cytometry was subsequently employed to assess *T. cruzi* epimastigotes’ type of death induced by exposure to **27** at 5 µM for 24 h. The labelling of 86% of the population of *T. cruzi* epimastigotes with annexin V indicated that these parasites probably were involved in an apoptotic process. However, the absence of labelling with propidium iodide was suggestive that their plasma membranes were intact (Figure 10). Following 48 h of treatment, 13% of epimastigotes were labelled only with propidium iodide, thereby demonstrating a necrotic death of this population. Nevertheless, phosphatidylserine (PS) exposure was still present, indicating that death by apoptosis was also occurring.

**Figure 11:**
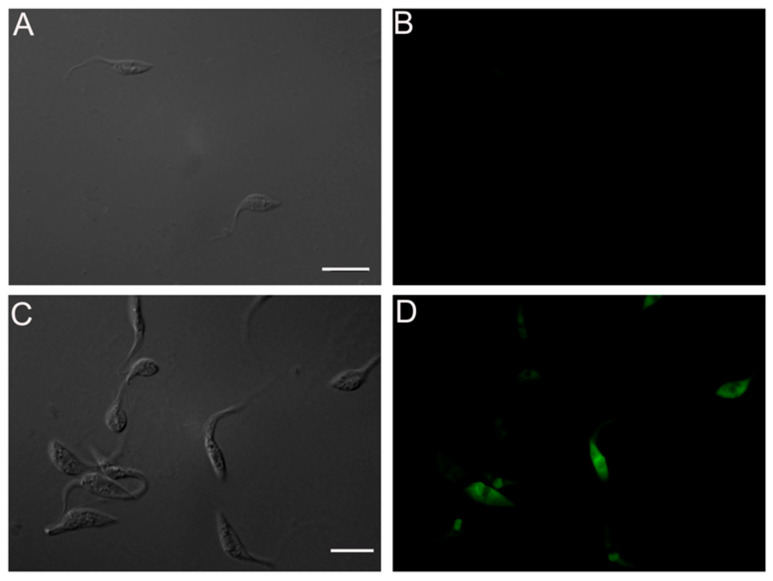
Immunofluorescence microscopy of *T. cruzi* epimastigotes labelled with the Click-iT TUNEL assay. (**A**,**B**) correspond to untreated cells and (**C**,**D**) represent cells treated with compound 27 at 5 µM for 24 h. Note that 60% of treated cells are marked (D-green) indicating that the main type of death used by epimastigotes after treatment is the apoptotic process. Bars = 15 µm.

To confirm the occurrence of apoptotic death after 24 h of treatment, epimastigotes were assessed using the Click-iT TUNEL assay for apoptosis, followed by fluorescence microscopy. The labelling of the epimastigotes with Annexin V/Alexa 488 was observed, suggesting that PS exposure and apoptotic death had taken place (Figure 11).

**Figure 12:**
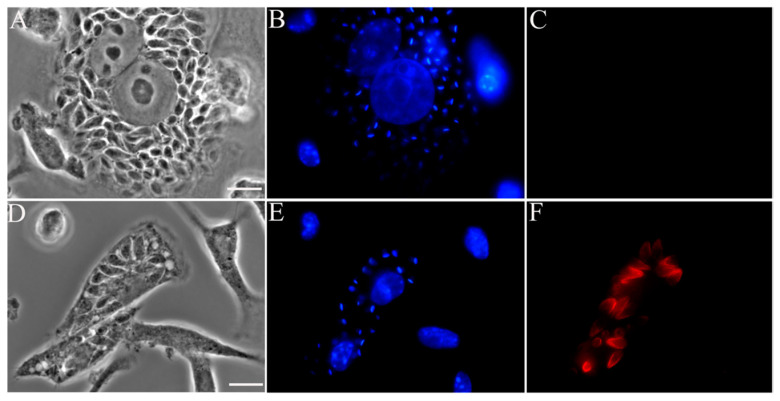
Immunofluorescence microscopy of *T. cruzi* amastigotes labelled with antibodies against LC3B (microtubule-associated protein light chain 3), an autophagic cell death marker (red), and DAPI, a nuclear marker (blue). **A**–**C** correspond to untreated cells and **D**–**F** represent cells treated with compound **27** (IC_50_). The LC3 marking (**F**) indicates that intracellular amastigotes use the death-moving autophagy process. Bar = 10 μm.

Programmed cell death by autophagy in intracellular amastigotes treated with compound **27**: 

Electron microscopy observations of *T. cruzi* intracellular amastigotes treated with **27** (data not shown) revealed that the parasites were surrounded by profiles of the endoplasmic reticulum, indicating the presence of autophagosomes. Therefore, we decided to monitor the autophagy process. To this end, infected macrophages were treated with the IC_50_ concentration of **27** and the anti-LC3B antibody was used to identify autophagic structures by fluorescence microscopy (Figure 12). Approximately 30% of amastigotes inside macrophages appeared strongly labelled after the treatment. No labelling was observed in untreated amastigotes, thereby suggesting that the treatment with **27** induces amastigote autophagy.

## 3. Materials and Methods

### 3.1. Chemistry

#### 3.1.1. General

Melting points were determined with a Buchi 510 apparatus (Buchi, Flawil, Switzerland) and are uncorrected. NMR spectra were recorded on Varian spectrometers (Varian, Palo Alto, CA, USA). ^1^H NMR spectra were recorded at 600 or 300 MHz, ^13^C NMR spectra at 150 or 75 MHz and were internally referenced to residual solvent signals. ^31^P NMR were recorded at 121.44 MHz. Chemical shifts are reported in *δ* units, parts per million (ppm). Electrospray ionization (ESI) mass spectra were recorded on a LC-MSn Fleet spectrometer (Thermo Scientific, Waltham, MA, USA) using MeOH as a solvent. HRMS spectra were recorded, in the ESI mode, on a UPLC-MSn Orbitrap Velos-Thermo spectrometer (Thermo Scientific, Waltham, MA, USA). Reactions under microwave irradiation were performed in a CEM Discover Lab Mate reactor (CEM Corporation, Matthews, NC, USA). Flash column chromatography (FCC) was performed on Merck silica gel 60 (230–400 mesh) and TLC on Merck 60 F254 films (0.2 mm) precoated glass plates. Spots were visualized with UV light at 254 nm and a PMA stain. All solvents were dried and/or purified according to standard procedures prior to use. All reagents employed in the present work were purchased from commercial suppliers and used without further purification. Reactions were run in flame-dried glassware under an atmosphere of argon. Active esters **45** [46] and **46** [47] were synthesized according to published procedures.

#### 3.1.2. General Procedure for the Synthesis of Alcohols **4**–**6**

To an ice-cold solution of acid **1**–**3** (20 mmol) in THF (30 mL), a borane dimethyl sulphide complex (2.7 mL, 28 mmol) was added dropwise. The solution was warmed at room temperature and stirred for 1 h at 45 °C. Then, the reaction was cooled at 0 °C, quenched with MeOH (60 mL) and concentrated under reduced pressure. The residue was dissolved in EtOAc, washed with brine, dried over Na_2_SO_4_ and concentrated in vacuo to obtain the projected alcohols **4**–**6,** which were used in the next step without further purification.

##### 5-bromopentan-1-ol (**4**)

It was synthesized according to the general procedure described above from 5-bromovaleric acid as a pale yellow solid (3.34 g, 100% yield); mp 72–74 °C; ^1^H-NMR (300 MHz, CDCl_3_) *δ* 3.66 (t, *J* = 6.2 Hz, 2H), 3.42 (t, *J* = 6.8 Hz, 2H), 1.94–1.85 (m, 2H), 1.62–1.48 (m, 4H); ^13^C-NMR (75 MHz, CDCl_3_) *δ* 62.5, 33.6, 32.4, 31.7, 24.4.

##### 6-bromohexan-1-ol (**5**)

It was synthesized according to the general procedure described above from 6-bromohexanoic acid as a colourless oil (3.51 g, 97% yield). ^1^H-NMR (300 MHz, CDCl_3_) *δ* 3.51 (t, *J* = 6.6 Hz, 2H), 3.33 (t, *J* = 6.6 Hz, 2H), 2.93 (br. s, 1H), 1.83–1.52 (m, 2H), 1.51–1.26 (m, 6H); ^13^C-NMR (75 MHz, CDCl_3_) *δ* 62.3, 33.9, 32.5, 32.3, 27.8, 24.8. 

##### 11-bromoundecan-1-ol (**6**)

It was synthesized according to the general procedure described above from 11-bromoundecanoic acid as a pale yellow solid (4.77 g, 95% yield); mp 45–47 °C; ^1^H-NMR (300 MHz, CDCl_3_) *δ* 3.63 (t, *J* = 6.6 Hz, 2H), 3.40 (t, *J* = 6.9 Hz, 2H), 1.89–1.80 (m, 2H), 1.60–1.51 (m, 2H), 1.48–1.24 (m, 15H); ^13^C-NMR (75 MHz, CDCl_3_) *δ* 63.0, 34.0, 32.8, 32.7, 29.5, 29.35, 29.32 (2C, 2CH_2_), 28.7, 28.1, 25.6.

#### 3.1.3. General Procedure for the Synthesis of Azides **7**–**9**

To a solution of the appropriate alcohol **4**–**6** (15 mmol) in DMF (30 mL), NaN_3_ (9.75 g, 150 mmol) was added. The reaction was stirred at 40 °C for 2 days, poured into water and extracted with EtOAc (3 × 50 mL). The organic layer was washed with brine, dried over Na_2_SO_4_ and evaporated under reduced pressure. The desired azides were obtained in pure form upon purification with FCC (petroleum ether/EtOAc 9:1).

##### 5-azidopentan-1-ol (**7**)

It was synthesized according to the general procedure described above from 5-bromopentan-1-ol (**4**). Colourless oil [48]; yield: 1.78 g, 92%; ^1^H-NMR (300 MHz, CDCl_3_) *δ* 3.65 (t, *J* = 6.4 Hz, 2H), 3.28 (t, *J* = 6.8 Hz, 2H), 1.68–1.55 (m, 4H), 1.50–1.39 (m, 2H); ^13^C-NMR (75 MHz, CDCl_3_) *δ* 62.8, 51.5, 32.2, 28.6, 23.3. 

##### 6-azidohexan-1-ol (**8**) 

It was synthesized according to the general procedure described above from 6-bromohexan-1-ol (**5**). Colourless oil [49]; yield: 2.0 g, 93%; ^1^H-NMR (300 MHz, CDCl_3_) *δ* 3.63 (t, *J* = 6.12 Hz, 2H), 3.26 (t, *J* = 6.72 Hz, 2H), 1.62–1.55 (m, 4H), 1.46–1.36 (m, 4H); ^13^C-NMR (75 MHz, CDCl_3_) *δ* 62.8, 51.4, 32.6, 28.8, 26.5, 25.3.

##### 11-azidoundecan-1-ol (**9**) 

It was synthesized according to the general procedure described above from 11-bromoundecan-1-ol (**6**). Colourless oil [50]; yield: 3.1 g, 97%; ^1^H-NMR (300 MHz, CDCl_3_) *δ* 3.47 (t, *J* = 6.72 Hz, 2H), 3.14 (t, *J* = 6.72 Hz, 2H), 1.50–1.41 (m, 4H), 1.17 (s, 14H); ^13^C-NMR (75 MHz, CDCl_3_) *δ* 62.4, 51.3, 32.6, 29.5, 29.3, 29.2, 29.0, 28.7 (2C, 2CH_2_), 26.5, 25.6.

#### 3.1.4. Benzyl undec-10-ynoate (**15**) 

To a solution of 10-undecynoic acid (1.0 g, 5.49 mmol), in DMF (4 mL), Cs_2_CO_3_ (0.9 g, 2.75 mmol) was added and the reaction was stirred at rt for 30 min. Then, benzyl bromide (0.65 mL, 5.49 mmol) was added and the reaction mixture was stirred overnight at rt. Upon completion, water was added and the aqueous phase was extracted twice with Et_2_O. The organic layers were combined, dried over Na_2_SO_4_, evaporated to dryness and the residue was then purified by FCC with Hex/EtOAc (95:5) to afford compound **15**.

Colourless oil [51]; yield: 1.39 g, 93%; ^1^H-NMR (300 MHz, CDCl_3_) *δ* 7.37–7.34 (m, 5H), 5.12 (s, 2H), 2.35 (t, *J* = 7.5 Hz, 2H), 2.17 (dt, *J* = 7.0, 2.6 Hz, 2H), 1.94 (t, *J* = 2.6 Hz, 1H), 1.67–1.62 (m, 2H), 1.54–1.47 (m, 2H), 1.43–1.31 (m, 8H); ^13^C-NMR (75 MHz, CDCl_3_) *δ* 173.6, 136.1, 128.8 (2C, Aryl), 128.5, 128.3 (2C, Aryl), 84.7, 68.3, 66.2, 34.5, 29.3, 29.2, 29.0, 28.7, 28.6, 25.0, 18.5.

#### 3.1.5. Benzyl dec-9-ynylcarbamate (**16**)

A mixture of 10-undecynoic acid 16 (1 g, 5.49 mmol), DPPA (1.42 mL, 6.58 mmol) and triethylamine (0.9 mL, 6.58 mmol), was refluxed in benzene (20 mL) for 1 h. Upon the formation of the intermediate (monitored by TLC), benzyl alcohol (1.25 mL, 12.06 mmol) was added and the reaction mixture was heated at 80 °C overnight. Then, the solvent was removed in vacuo and the residue was diluted with EtOAc, washed with 0.4 N HCl and aq. saturated NaHCO_3_. Pure 16 was obtained after FCC purification (Hex/EtOAc = 98:2). Light yellow oil; yield: 1.04 g, 66%; ^1^H-NMR (300 MHz, CDCl_3_) *δ* 7.37–7.30 (m, 5H), 5.09 (s, 2H), 4.73 (br.s, 1H), 3.26–3.15 (m, 2H), 2.17 (dt, *J* = 7.0 and 2.6 Hz, 2H), 1.95–1.93 (m, 1H), 1.54–1.49 (m, 4H), 1.39–1.30 (m, 8H); ^13^C-NMR (75 MHz, CDCl_3_) *δ* 156.4, 136.8, 130.0, 128.8 (2C, Aryl), 128.7 (2C, Aryl), 84.8, 68.3, 66.7, 41.2, 30.1, 29.2, 29.1, 28.8, 28.7, 28.6, 18.5.

#### 3.1.6. General Procedure for the Synthesis of Triazoles **17**–**24**

To a solution of the appropriate alkyne (1 equiv, 1 mmol) in a mixture of H_2_O/^t^BuOH 1:4 (22 mL) the appropriate azide (2 equiv), CuSO_4_∙5H_2_O (0.3 equiv) and sodium *L*(+)-ascorbate (0.6 equiv) were added sequentially. The resulting mixture was stirred at room temperature for 12 h. The reaction was cooled to 0 °C and partitioned between CH_2_Cl_2_ and H_2_O. The organic layer was washed with aq. NH_4_OH solution and brine until neutral pH and the solvent was evaporated in vacuo. The projected triazoles were obtained after FCC purification.

##### 11-(4-pentyl-1H-1,2,3-triazol-1-yl)undecan-1-ol (**17**)

It was synthesized according to the general procedure described above using hept-1-yne (**10**) and 11-azidoundecan-1-ol (**9**). White solid; mp 70–73 °C; yield 80%; ^1^H-NMR (300 MHz, CDCl_3_) *δ* 7.23 (s, 1H), 4.28 (t, *J* = 7.32 Hz, 2H), 3.62 (t, *J* = 6.69 Hz, 2H), 2.69 (t, *J* = 7.32 Hz, 2H), 1.88–1.84 (m, 2H), 1.68–1.52 (m, 4H), 1.34–1.25 (m, 18H), 0.88 (t, *J* = 6.72 Hz, 3H); ^13^C-NMR (75 MHz, CDCl_3_) *δ* 148.9, 120.3, 62.6, 50.0, 32.7, 31.3, 30.2, 29.4, 29.3, 29.0 (2C, 2CH_2_), 28.2, 27.8, 26.3, 25.6, 25.5, 22.3, 14.1; HRMS (ESI) (m/z): calcd for C_18_H_36_N_3_O (M + H)^+^ 310.2858, found 310.2848.

##### 6-(4-decyl-1H-1,2,3-triazol-1-yl)hexan-1-ol (**18**)

It was synthesized according to the general procedure described above using undec-1-yne (**11**) and 6-azidohexan-1-ol (**8**). White solid; yield 65%; ^1^H-NMR (300 MHz, CDCl_3_): *δ* 7.68 (s, 1H), 4.31 (t, *J* = 7.32 Hz, 2H), 3.48 (t, *J* = 6.72 Hz, 2H), 2.63 (t, *J* = 7.32 Hz, 2H), 1.90–1.80 (m, 2H), 1.63–1.59 (m, 2H), 1.49–1.42 (m, 2H), 1.40–1.24 (m, 18H), 0.85 (t, *J* = 7.35 Hz, 3H).

##### 11-(4-decyl-1H-1,2,3-triazol-1-yl)undecan-1-ol (**19**)

It was synthesized according to the general procedure described above using undec-1-yne (**11**) and 11-azidoundecan-1-ol (**9**). White solid; mp 85–88 °C; yield 72%; ^1^H-NMR (300 MHz, CDCl_3_) *δ* 7.22 (s, 1H), 4.25 (t, *J* = 7.2 Hz, 2H), 3.59 (t, *J* = 6.6 Hz, 2H), 2.65 (t, *J* = 7.5 Hz, 2H), 2.10 (br.s, 1H), 1.83 (t, *J* = 6.6 Hz, 2H), 1.64–1.49 (m, 4H), 1.26–1.22 (m, 26H), 0.83 (t, *J* = 6.6 Hz, 3H); ^13^C-NMR (75 MHz, CDCl_3_) *δ* 148.6, 120.5, 63.1, 50.3, 32.9, 32.0, 30.5, 29.8. 29.7, 29.6 (2C, 2CH_2_), 29.57, 29.54, 29.52, 29.4 (2C, 2CH_2_), 29.1, 26.6, 25.5, 22.8, 14.3.

##### 11-(4-(cyclopentylmethyl)-1H-1,2,3-triazol-1-yl)undecan-1-ol (**20**)

It was synthesized according to the general procedure described above using prop-2-yn-1-ylcyclopentane (**12**) and 11-azidoundecan-1-ol (**9**). White solid; mp 64–68 °C; yield 76%; ^1^H-NMR (300 MHz, CDCl_3_) *δ* 7.22 (s, 1H), 4.27 (t, *J* = 7.32 Hz, 2H), 3.61 (t, *J* = 6.72 Hz, 1H), 2.69 (d, *J* = 7.32 Hz, 2H), 2.20–2.10 (m, 1H), 1.83–1.23 (m, 26H); ^13^C-NMR (75 MHz, CDCl_3_) *δ* 148.2, 121.0, 63.2, 50.5, 40.3, 33.1, 32.8 (2C, 2CH_2_), 32.1, 30.6, 29.7 (3C, 3CH_2_), 29.2 26.7, 26.0, 25.5, 24.9 (2C, 2CH_2_); HRMS (ESI) (m/z): calcd for C_19_H_36_N_3_O (M + H)^+^ 322.2858, found 322.2854, calcd for C_19_H_35_N_3_ONa (M + Na)^+^ 344.2678, found 344.2669.

##### 11-(4-(cyclohexylmethyl)-1H-1,2,3-triazol-1-yl)undecan-1-ol (**21**)

It was synthesized according to the general procedure described above using prop-2-yn-1-ylcyclohexane (**13**) and 11-azidoundecan-1-ol (**9**). White solid; mp 62–65 °C; yield 79%; ^1^H-NMR (300 MHz, CDCl_3_) δ 7.25 (s, 1H), 4.28 (t, *J* = 7.2 Hz, 2H), 3.62 (t, *J* = 6.6 Hz, 2H), 2.57 (d, *J* = 6.9 Hz, 2H), 1.92–1.84 (m, 1H), 1.74–1.18 (m, 28H); ^13^C-NMR (75 MHz, CDCl_3_) δ 146.8, 121.1, 63.0, 50.2, 38.2, 33.5, 33.1 (2C, 2CH_2_), 32.9, 30.4, 29.6, 29.44, 29.41, 29.40, 29.0, 26.5 (2C, 2CH_2_), 26.3 (2C, 2CH_2_), 25.8.; HRMS (ESI) (m/z): calcd for C_20_H_38_N_3_O (M + H)^+^ 336.3015, found 336.3016, calcd for C_20_H_37_N_3_ONa (M + Na)^+^ 358.2834, found 358.2833.

##### Benzyl 9-(1-(11-hydroxyundecyl)-1H-1,2,3-triazol-4-yl)nonanoate (**22**)

It was synthesized according to the general procedure described above using benzyl undec-10-ynoate (**15**) and 11-azidoundecan-1-ol (**9**). White solid; mp 88–89 °C; yield 58%; ^1^H-NMR (300 MHz, CDCl_3_) *δ* 7.36–7.33 (m, 5H), 7.23 (s, 1H), 5.11 (s, 2H), 4.29 (t, *J* = 7.2 Hz, 2H), 3.63 (t, *J* = 6.6 Hz, 2H), 2.69 (m, 2H), 2.34 (t, *J* = 7.5 Hz, 2H), 1.89–1.85 (m, 2H), 1.64–1.50 (m, 6H), 1.29–1.26 (m, 22H); ^13^C-NMR (75 MHz, CDCl_3_) *δ* 173.8, 148.4, 136.2, 128.7 (2C, Aryl), 128.3 (2C, Aryl), 120.5, 120.4, 66.1, 63.1, 50.3, 34.4, 32.9, 30.4, 29.59, 29.56, 29.48, 29.46, 29.42, 29.28, 29.25, 29.2, 29.0, 26.6, 25.8, 25.78, 25.76, 25.0; HRMS (ESI) (m/z): calcd for C_29_H_48_N_3_O_3_ (M + H)^+^ 486.3696, found 486.3696, calcd for C_29_H_47_N_3_O_3_Na (M + Na)^+^ 508.3515, found 508.3521.

##### Benzyl 8-(1-(5-hydroxypentyl)-1H-1,2,3-triazol-4-yl)octylcarbamate (**23**)

It was synthesized according to the general procedure described above using benzyl dec-9-ynylcarbamate (**16**) and 5-azidopentan-1-ol (**7**). White solid; mp 77–79 °C; yield 51%; ^1^H-NMR (300 MHz, CDCl_3_) *δ* 7.34–7.30 (m,5H), 7.24 (s, 1H), 5.09 (s, 2H), 4.73 (br.s, 1H), 4.32 (t, *J* = 7.1 Hz, 2H), 3.64 (t, *J* = 6.4 Hz, 2H), 3.20–3.14 (m, 2H), 2.69 (t, *J* = 7.8 Hz, 2H), 1.98–1.88 (m, 2H), 1.67–1.30 (m, 16H); ^13^C-NMR (75 MHz, CDCl_3_) *δ* 156.5, 148.4, 136.8, 128.7 (2C, Aryl), 128.6, 128.2 (2C, Aryl), 123.4, 66.7, 62.5, 50.2, 41.2, 32.0, 30.2, 30.1, 29.5, 29.3, 29.24, 29.21, 26.8, 25.8, 23.0; HRMS (ESI) (m/z): calcd for C_23_H_37_N_4_O_3_ (M + H)^+^ 417.2866, found 417.2855, calcd for C_23_H_36_N_4_O_3_Na (M + Na)^+^ 439.2685, found 439.2670.

##### Benzyl 8-(1-(11-hydroxyundecyl)-1H-1,2,3-triazol-4-yl)octylcarbamate (**24**)

It was synthesized according to the general procedure described above using benzyl dec-9-ynylcarbamate (**16**) and 11-azidoundecan-1-ol (**9**). White solid; mp 86–88 °C; yield 60%; ^1^H-NMR (300 MHz, CDCl_3_) *δ* 7.36–7.31 (m, 5H), 7.23 (s, 1H), 5.09 (s, 2H), 4.73 (br.s, 1H), 4.29 (t, *J* = 7.0 Hz, 2H), 3.63 (t, *J* = 6.6 Hz, 2H), 3.14–3.21 (m, 2H), 2.69 (t, *J* = 7.7 Hz, 2H), 1.89–1.85 (m, 2H), 1.66–1.26 (m, 28H); ^13^C-NMR (75 MHz, CDCl_3_) *δ* 156.6, 148.3, 136.6, 128.4 (2C, Aryl), 128.1, 128.0 (2C, Aryl), 120.3, 66.6, 63.0, 50.1, 41.1, 32.8, 30.3, 29.5, 29.4, 29.3 (2C), 29.27, 29.2, 29.1, 28.94, 28.89, 26.6, 26.4, 25.7, 25.6.; HRMS (ESI) (m/z): calcd for C_29_H_49_N_4_O_3_ (M + H)^+^ 501.3805, found 501.3790, calcd for C_29_H_48_N_4_O_3_Na (M + Na)^+^ 523.3624, found 523.3616

#### 3.1.7. General Procedure for the Synthesis of Ether Phospholipids (Method A)

To a cold solution at −5 °C of POCl_3_ (1.3 equiv) in THF (0.2 M), a mixture of alcohol **17**–**24** (1 equiv, 1 mmol) and Et_3_N (1.8 equiv) in THF (0.15 M) was added dropwise. The reaction mixture was stirred at this temperature until the consumption of alcohol. Then, water was added and stirring was continued for 30 min. The aqueous layer was extracted with EtOAc and then with CH_2_Cl_2_. The combined organic extracts were dried over anhydrous Na_2_SO_4_ and the solvent was evaporated in vacuo to afford the corresponding phosphoric acid derivative, which was converted to its corresponding pyridinium salt upon addition of anhydrous pyridine and stirring for 2.5 h at 45 °C. After cooling, the solvent was evaporated in vacuo and pyridine (5 mL) was added to the residue. To the resulting solution were sequentially added choline or homocholine *p*-toluenesulfonate (1.5 equiv) and TPSCl (1.8 equiv) at 0 °C and the mixture was stirred at ambient temperature for 48 h. Subsequently, the mixture was hydrolysed by the addition of 2-propanol/H_2_O (7:3). The mixture was stirred for 1 h, the solvent was evaporated in vacuo and the residue was subjected to FCC (CH_2_Cl_2_/MeOH/NH_4_OH = 95:5:0.5–60:40:0.5) to afford the desired phospholipid derivative.

##### 11-(4-pentyl-1H-1,2,3-triazol-1-yl)undecyl (2-(trimethylammonio)ethyl) phosphate inner salt (**25**)

It was synthesized according to the general procedure described above from alcohol **17** and choline *p*-toluenesulfonate. Light yellow gummy oil; yield 46%; ^1^H-NMR (600 MHz, CDCl_3_) *δ* 7.66 (s, 1H), 4.30 (t, *J* = 7.32 Hz, 2H), 4.20 (br.s, 2H), 3.82 (q, *J* = 6.6 Hz, 2H), 3.58 (m, 2H), 3.19 (s, 9H), 2.63 (t, *J* = 7.92 Hz, 2H), 1.85–1.80 (m, 2H), 1.64–1.55 (m, 4H), 1.30–1.24 (m, 18H), 0.86 (t, *J* = 6.72 Hz, 3H); ^13^C-NMR (150 MHz, CDCl_3_) *δ* 148.3, 120.4, 64.9 (d, *J_C,P_* = 5.6 Hz), 59.3 (d, *J_C,P_* = 5.0 Hz), 54.3 (3C, N(CH_3_)_3_), 51.0, 50.0, 31.4, 30.8 (2C), 30.3, 29.55, 29.51, 29.1, 28.9, 28.8, 26.4, 25.6, 25.2, 22.3, 13.9 ppm; ^31^P-NMR (121 MHz, CDCl_3_) *δ*−0.73; HRMS (ESI) (m/z): calcd for C_23_H_48_N_4_O_4_P (M + H)^+^ 475.3413, found 475.3415.

##### 6-(4-decyl-1H-1,2,3-triazol-1-yl)hexyl (2-(trimethylammonio)ethyl) phosphate inner salt (**26**)

It was synthesized according to the general procedure described above from alcohol **18** and choline *p*-toluenesulfonate. Light yellow gummy oil; yield 42%; ^1^H-NMR (300 MHz, CDCl_3_) *δ* 7.16 (s, 1H), 4.13–4.10 (m, 4H), 3.71–3.61 (m, 4H), 3.26 (s, 9H), 2.51 (t, *J* = 7.32 Hz, 2H), 1.70 (br.s, 2H), 1.48–1.41 (m, 4H), 1.11 (br.s, 18H), 0.71 (br.s, 3H); ^13^C-NMR (75 MHz, CDCl_3_) *δ* 148.2, 120.5, 66.0 (d, *J_C,P_* = 5.6 Hz), 64.8 (d, *J_C,P_* = 5.0 Hz), 54.0 (3C, N(CH_3_)_3_), 49.7, 31.7, 31.6, 30.6, 30.0, 29.5, 29.4, 29.1, 29.0, 28.9, 25.9, 25.6, 25.5, 25.0, 22.5, 13.9 ppm; ^31^P-NMR (121 MHz, CDCl_3_) *δ*−0.66; HRMS (ESI) (m/z): calcd for C_23_H_48_N_4_O_4_P (M + H)^+^ 475.3413, found 475.3428.

##### 11-(4-decyl-1H-1,2,3-triazol-1-yl)undecyl (2-(trimethylammonio)ethyl) phosphate inner salt (**27**)

It was synthesized according to the general procedure described above from alcohol **19** and choline *p*-toluenesulfonate. Light yellow gummy oil; yield 48%; ^1^H-NMR (600 MHz, CD_3_OD) *δ* 7.66 (s, 1H), 4.29 (t, *J* = 6.72 Hz, 2H), 4.19 (br.s, 2H), 3.80 (t, *J* = 6.15 Hz, 2H), 3.58 (t, *J* = 4.26 Hz, 2H), 3.17 (s, 9H), 2.60 (t, *J* = 6.72 Hz, 2H), 1.79 (t, *J* = 6.69 Hz, 2H), 1.54 (t, *J* = 6.69 Hz, 4H), 1.17 (s, 26H), 0.79 (t, *J* = 5.49 Hz, 3H); ^13^C-NMR (150 MHz, CD_3_OD) *δ* 147.7, 122.4, 66.5, 66.1 (d, *J_C,P_* = 6.2 Hz), 59.3 (d, *J_C,P_* = 5.6 Hz), 54.0 (3C, N(CH_3_)_3_), 49.8, 31.9, 30.3 (2C), 30.2 (2C), 29.7 (2C), 29.51, 29.48, 29.10, 29.0, 28.9, 28.8, 28.4, 25.8, 25.3, 24.6, 22.1, 13.6; ^31^P-NMR (121 MHz, CD_3_OD) *δ* 0.26; HRMS (ESI) (m/z): calcd for C_28_H_58_N_4_O_4_P (M + H)^+^ 545.4196, found 545.4169.

##### 11-(4-(cyclopentylmethyl)-1H-1,2,3-triazol-1-yl)undecyl (2-(trimethylammo nio)ethyl) phosphate inner salt (**28**)

It was synthesized according to the general procedure described above from alcohol **20** and choline *p*-toluenesulfonate. Light yellow gummy oil; yield 42%; ^1^H-NMR (600 MHz, CDCl_3_) *δ* 7.25 (s, 1H), 4.24 (m, 4H), 3.73 (m, 4H), 3.32 (s, 9H), 2.65 (d, *J* = 7.5 Hz, 2H), 2.13 (m, 1H), 1.81–1.48 (m, 10H), 1.23–1.20 (m, 16H); ^13^C-NMR (150 MHz, CDCl_3_) *δ* 147.7, 120.6, 66.0, 65.4 (d, *J_C,P_* = 5.6 Hz), 59.1 (d, *J_C,P_* = 5.0 Hz), 54.1 (3 C, N(CH_3_)_3_), 50.0, 39.9, 32.3 (2C), 31.6, 30.9, 30.2, 29.5, 29.4, 29.3, 28.8, 26.8, 26.4, 25.8, 25.0 (2C, 2CH_2_); ^31^P-NMR (121 MHz, CDCl_3_) *δ*−0.65; HRMS (ESI) (m/z): calcd for C_24_H_48_O_4_N_4_P (M + H)^+^ 487.3408 found 487.3412, calcd for C_24_H_47_O_4_N_4_NaP (M + Na)^+^ 509.3227, found 509.3225.

##### 11-(4-(cyclohexylmethyl)-1H-1,2,3-triazol-1-yl)undecyl (2-(trimethylammonio) ethyl) phosphate inner salt (**29**)

It was synthesized according to the general procedure described above from alcohol **21** and choline *p*-toluenesulfonate. Light yellow gummy oil; yield 46%; ^1^H-NMR (600 MHz, CD_3_OD) *δ* 7.25 (s, 1H), 4.24–4.16 (m, 4H), 3.72 (br.s, 2H), 3.59 (br.s, 2H), 3.18 (s, 9H), 2.50 (m, 2H), 1.79 (br.s, 2H), 1.63–1.49 (m, 8H), 1.21–1.11 (m, 18H); ^13^C-NMR (150 MHz, CDCl_3_) *δ* 146.7, 121.0, 66.1, 65.5 (d, *J_C,P_* = 5.9 Hz), 59.1 d, *J_C,P_* = 5.0 Hz), 54.1 (3C, N(CH_3_)_3_), 50.0, 38.0, 33.3, 32.9 (2C, 2CH_2_), 31.0, 30.2, 29.48, 29.44, 29.3, 29.2, 28.9, 26.49, 26.45, 26.1, 26.0, 25.9; ^31^P-NMR (121 MHz, CD_3_OD) δ−0.85; HRMS (ESI) (m/z): calcd for C_25_H_50_N_4_O_4_P (M + H)^+^ 501.3570, found 501.3556.

##### 11-(4-(9-(benzyloxy)-9-oxononyl)-1H-1,2,3-triazol-1-yl)undecyl (2-(trimethyl ammonio)ethyl) phosphate inner salt (**30**)

It was synthesized according to the general procedure described above from alcohol 22 and choline *p*-toluenesulfonate. Light yellow gummy oil; yield 31%; ^1^H-NMR (600 MHz, CD_3_OD) *δ* 7.70 (s, 1H), 7.35–7.31 (m, 5H), 5.10 (s, 2H), 4.34 (t, *J* = 7.0 Hz, 2H), 4.25–4.23 (m, 2H), 3.86 (q, *J* = 6.5 Hz, 2H), 3.63 (dd, *J* = 5.9 and 3.3 Hz, 2H), 3.22 (s, 9H), 2.67 (t, *J* = 7.5 Hz, 2H), 2.35 (t, *J* = 7.3 Hz, 2H), 1.92–1.85 (m, 2H), 1.67–1.60 (m, 2H), 1.37–1.29 (m, 22H); ^13^C-NMR (150 MHz, CD_3_OD) *δ* 175.2, 149.2, 137.7, 129.5 (2C, Aryl), 129.2, 129.17 (2C, Aryl), 123.1, 67.7, 67.3 (m), 67.1, 66.9 (d, *J_C,P_* = 5.9 Hz), 60.2 (d, *J_C,P_* = 5.0 Hz), 54.7, 54.68, 54.63, 51.21, 35.05, 31.9 (d, *J_C,P_* = 7.4 Hz), 31.3, 30.7, 30.6, 30.54, 30.50, 30.45, 30.2, 30.19, 30.05, 30.03, 27.5, 26.9, 26.2, 26.0; ^31^P-NMR (121 MHz, CD_3_OD) *δ*−0.05; HRMS (ESI) (m/z): calcd for C_34_H_59_N_4_O_6_P (M + H)^+^ 651.4245, found 651.4245; calcd for C_34_H_59_N_4_O_6_NaP (M + Na)+ 673.4064, found 673.4057.

##### 5-(4-(8-(((benzyloxy)carbonyl)amino)octyl)-1H-1,2,3-triazol-1-yl)pentyl (2-(trimethylammonio)ethyl) phosphate inner salt (**31**)

It was synthesized according to the general procedure described above from alcohol **23** and choline *p*-toluenesulfonate. Light yellow gummy oil; yield 39%; ^1^H-NMR (600 MHz, CDCl_3_) *δ* 7.33–7.26 (m, 6H), 5.07 (s, 2H), 4.30–4.25 (m, 4H), 3.78–3.73 (m, 4H), 3.34 (s, 9H), 3.16–3.13 (m, 2H), 2.64 (t, *J* = 7.8 Hz, 2H), 1.87–1.83 (m, 2H), 1.63–1.28 (m, 16H); ^13^C-NMR (150 MHz, CD_3_OD) *δ* 158.9, 149.2, 138.5, 129.4 (2C, Aryl), 128.7 (2C, Aryl), 123.1, 123.0, 67.5, 67.2, 66.4 (d, *J_C,P_* = 5.9 Hz), 60.2 (d, *J_C,P_* = 5.0 Hz), 54.7, 54.69, 54.64, 51.1, 41.8, 31.0 (d, *J_C,P_* = 7.4 Hz), 30.9 (2C, 2CH_2_), 30.87, 30.5, 30.3, 30.1, 27.7, 26.3, 23.9; ^31^P-NMR (121 MHz, CD_3_OD) δ−0.85; HRMS (ESI) (m/z): calcd for C_28_H_49_O_6_N_5_P (M + H)^+^ 582.3415, found 582.3419.

##### 11-(4-(8-(((benzyloxy)carbonyl)amino)octyl)-1H-1,2,3-triazol-1-yl)undecyl (2-(trimethylammonio)ethyl) phosphate inner salt (**32**)

It was synthesized according to the general procedure described above from alcohol 24 and choline *p*-toluenesulfonate. Light yellow gummy oil; yield 32%; ^1^H-NMR (600 MHz, CD_3_OD) *δ* 7.71 (s, 1H), 7.34–7.26 (m, 5H), 5.05 (s, 2H), 4.34 (t, J = 7.0 Hz, 2H), 4.26–4.22 (m, 2H), 3.86 (q, J = 6.5 Hz, 2H), 3.64–3.60 (m, 2H), 3.22 (s, 9H), 3.09 (t, J = 7.0 Hz, 2H), 2.67 (t, J = 7.6 Hz, 2H), 1.91–1.85 (m, 2H), 1.65–1.29 (m, 28H); ^13^C-NMR (150 MHz, CD_3_OD) *δ* 158.8, 149.1, 138.5, 129.4 (2C, Aryl), 128.9, 128.7 (2C, Aryl), 123.0, 67.5 (m), 67.2, 66.9 (d, *J_C,P_* = 5.9 Hz), 60.3 (d, *J_C,P_* = 5.9 Hz), 54.7, 54.67, 54.62, 51.2, 41.8, 31.9, 31.8, 31.2, 30.8, 30.7, 30.6, 30.5 (2C, 2CH_2_), 30.4, 30.3, 30.2, 30.0, 27.7, 27.4, 26.9, 26.2; ^31^P-NMR (121 MHz, CD_3_OD) *δ*−0.064; HRMS (ESI) (m/z): calcd for C_34_H_61_N_5_O_6_P (M + H)^+^ 666.4354, found 666.4360, calcd for C_34_H_60_N_5_O_6_NaP (M + Na)+ 688.4173, found 688.4174.

##### 11-(4-(cyclohexylmethyl)-1H-1,2,3-triazol-1-yl)undecyl (3-(trimethylammonio) propyl) phosphate inner salt (**33**)

It was synthesized according to the general procedure described above from alcohol **21** and homocholine *p*-toluenesulfonate. Light yellow gummy oil; yield 41%; ^1^H-NMR (600 MHz, CDCl_3_) *δ* 7.22 (s, 1H), 4.26 (t, J = 7.2 Hz, 2H), 3.92–3.90 (m, 2H), 3.77–3.70 (m, 4H), 3.31 (s, 9H), 2.53 (d, J = 6.8 Hz, 2H), 2.10–2.05 (m, 2H), 1.86–1.82 (m, 2H), 1.68–1.49 (m, 7H), 1.26–1.14 (m, 18H), 0.97–0.89 (m, 2H); ^13^C-NMR (150 MHz, CDCl_3_) *δ* 146.9, 121.3, 65.7(d, *J_C,P_* = 7 Hz), 64.2, 62.0(d, *J_C,P_* = 5.4 Hz), 53.4 (3C, N(CH_3_)_3_), 50.2, 38.3, 33.6, 33.2 (2C, 2CH_2_), 31.0 (d, *J_C,P_* = 7.6 Hz), 30.5, 29.8, 29.7, 29.6, 29.2, 26.7, 26.6, 26.57, 26.3 (2C, 2CH_2_), 26.0, 24.7; ^31^P-NMR (121 MHz, CDCl_3_) δ−0.33; HRMS (ESI) (m/z): calcd for C_26_H_52_N_4_O_4_P (M + H)^+^ 515.3721, found 515.3726; calcd for C_26_H_52_N_4_O_4_NaP (M + Na)^+^ 537.3540, found 537.3540.

#### 3.1.8. General Procedure for the Hydrogenolysis (Compounds **34**–**36**)

To the solution of the appropriate benzyl ester or benzyl carbamate (1 mmol) in MeOH (0.1 M), Pd/C (10% *w*/*w*) was added and the resulting mixture was stirred at room temperature overnight under H_2_ atmosphere. The mixture was passed through a celite pad and washed with MeOH/NH_4_OH (30:1). The filtrate was evaporated to dryness to give the projected compounds.

##### 11-(4-(8-carboxyoctyl)-1H-1,2,3-triazol-1-yl)undecyl (2-(trimethylammonio) ethyl) phosphate inner salt (**34**)

It was synthesized according to the general procedure described above from ether phospholipid **30**. White gummy solid; yield 95%; ^1^H-NMR (600 MHz, CD_3_OD) *δ* 7.73 (s, 1H), 4.35 (t, *J* = 7.0 Hz, 2H), 4.25 (s, 2H), 3.87 (q, *J* = 6.5 Hz, 2H), 3.65–3.62 (m, 2H), 3.23 (s, 9H), 2.68 (t, *J* = 7.5 Hz, 2H), 2.19 (t, *J* = 7.5 Hz, 2H), 1.89–1.86 (m, 2H), 1.65–1.61 (m, 6H), 1.33–1.31 (m, 22H); ^13^C-NMR (150 MHz, CD_3_OD) *δ* 149.2, 123.1, 67.5, 67.48, 67.4, 66.9 (d, *J_C,P_* = 5.9 Hz), 60.3 (d, *J_C,P_* = 4.9 Hz), 54.7, 54.68, 54.63, 51.2, 37.7, 31.9 (d, *J_C,P_* = 7.4 Hz), 31.3, 30.7, 30.6, 30.54 (2C, 2CH_2_), 30.46, 30.4, 30.2, 30.1, 27.5, 27.2, 26.9, 26.3, 23.9; ^31^P-NMR (121 MHz, CD_3_OD) *δ*−0.04; HRMS (ESI) (m/z): calcd for C_27_H_54_N_4_O_6_P (M + H)^+^ 561.3775, found 561.3773; calcd for C_27_H_54_N_4_O_6_NaP (M + Na)^+^ 583.3595, found 583.3586.

##### 5-(4-(8-aminooctyl)-1H-1,2,3-triazol-1-yl)pentyl (2-(trimethylammonio)ethyl) phosphate inner salt (**35**)

It was synthesized according to the general procedure described above from ether phospholipid **31**. Yellowish oil; yield 90%; ^1^H-NMR (600 MHz, CD_3_OD) *δ* 7.74 (s, 1H), 4.37 (t, *J* = 6.9 Hz, 2H), 4.25–4.21 (m, 2H), 3.86 (q, *J* = 6.4 Hz, 2H), 3.65–3.62 (m, 2H), 3.23 (s, 9H), 2.75–2.64 (m, 4H), 1.97–1.88 (m, 2H), 1.69–1.35 (m, 16H); ^13^C-NMR (150 MHz, CD_3_OD) *δ* 149.2, 123.1, 67.5 (m), 66.5 (d, *J_C,P_* = 5.9 Hz), 60.3 (d, *J_C,P_* = 5.0 Hz), 54.8. 54.7, 54.6, 51.1, 41.8, 31.7, 31.1 (d, *J_C,P_* = 7.4 Hz), 30.9, 30.5, 30.4, 29.4, 28.1, 27.7, 26.2, 23.9; ^31^P-NMR (121 MHz, CD_3_OD) *δ*−0.12; HRMS (ESI) (m/z): calcd for C_20_H_43_N_5_O_4_P (M + H)^+^ 448.3047, found 448.3041.

##### 11-(4-(8-aminooctyl)-1H-1,2,3-triazol-1-yl)undecyl (2-(trimethylammonio) ethyl) phosphate inner salt (**36**)

It was synthesized according to the general procedure described above from ether phospholipid **32**. Yellowish oil; yield 97%; ^1^H-NMR (600 MHz, CD_3_OD) *δ* 7.71 (s, 1H), 4.35 (t, *J* = 7.0 Hz, 2H), 4.25–4.20 (m, 2H), 3.86 (q, *J* = 6.5 Hz, 2H), 3.64–3.61 (m, 2H), 3.22 (s, 9H), 2.68 (t, *J* = 7.5 Hz, 2H), 2.35–2.29 (m, 2H), 1.90–1.85 (m, 2H), 1.66–1.29 (m, 28H); ^13^C-NMR (150 MHz, CD_3_OD) *δ* 149.1, 123.0, 67.4 (m), 66.8 (d, *J_C,P_* = 5.9 Hz), 60.7, 60.2 (d, *J_C,P_* = 5.0 Hz), 54.7, 54.68, 54.63, 51.2, 45.3, 31.9, 31.8, 31.3, 30.7, 30.58(m), 30.53, 30.4, 30.3, 30.1, 30.0, 28.5, 28.2, 27.5, 26.9, 26.2; ^31^P-NMR (121 MHz, CD_3_OD) *δ*−0.041; HRMS (ESI) (m/z): calcd for C_26_H_55_N_5_O_4_P (M + H)^+^ 532.3987, found 532.4072.

#### 3.1.9. 11-bromoundecanal oxime (**38**)

To a solution of 11-bromoundecanal (**37**) (0.317 g, 1.27 mmol) in ^t^BuOH/H_2_O (1:1, 5 mL) hydroxylamine hydrochloride (0.093 g, 1.34 mmol) was added followed by 1 M aq. solution NaOH (1.3 mL). The resulting mixture was stirred at rt for 1.5 h. The reaction mixture was extracted with EtOAc. The organic layer washed with brine, dried over Na_2_SO_4_ and concentrated under reduced pressure to give oxime 38. White solid; mp 73–74 °C; yield: 0.319g, 95%; ^1^H-NMR (300 MHz, CDCl_3_) *δ* 8.00 (br.s, 1H), 6.71 (t, *J* = 5.5 Hz, 1H), 3.40 (t, *J* = 6.9 Hz, 2H), 2.37 (dt, *J* = 7.5 and 5.5 Hz, 2H), 1.90–1.80 (m, 2H), 1.56–1.39 (m, 2H), 1.36–1.25 (m, 12H); ^13^C-NMR (75 MHz, CDCl_3_) *δ* 153.1, 34.0, 32.8, 29.34 (2C, 2CH_2_), 29.31, 29.2, 28.7, 28.1, 26.0, 24.9; HRMS (ESI) (m/z): calcd for C_11_H_24_NO^79^Br (M + H)^+^ 264.0958, found 264.0957.

#### 3.1.10. 3-(10-bromodecyl)-5-decylisoxazole (**39**)

1-dodecyne (0.364 mL, 1.70 mmol) was added dropwise to a cooled solution (−5 °C/0 °C) of Et_3_N (0.05 mL) in CH_2_Cl_2_ (4 mL). A solution of oxime **38** (0.300 g, 1.14 mmol) in CH_2_Cl_2_ (3 mL) was added dropwise followed by sodium hypochlorite (2.7 mL, 5% of chloro active solution). The resulting mixture was stirred vigorously at room temperature for 24 h. Then, it was extracted with EtOAc and washed with water and brine. The organic layer was dried over Na_2_SO_4_ and concentrated under reduced pressure. The residue was subjected to FCC (petroleum ether/EtOAc = 99:1–98:2) to give compound 39. White solid; mp 45–47 °C; yield: 0.21 g, 43%; ^1^H-NMR (300 MHz, CDCl_3_) *δ* 5.79 (s, 1H), 3.40 (t, *J* = 6.9 Hz, 2H), 2.69 (t, *J* = 7.6 Hz), 2.60 (t, *J* = 7.7 Hz), 1.89–1.80 (m, 2H), 1.73–1.58 (m, 4H), 1.48–1.25 (m, 26H), 0.88 (t, *J* = 6.7 Hz, 3H); ^13^C-NMR (75 MHz, CDCl_3_) *δ* 173.3, 164.0, 100.2, 34.0, 32.8, 31.9, 29.6, 29.5, 29.4, 29.3, 29.28, 29.2, 29.16, 29.1 (2C, 2CH_2_), 28.7, 28.3, 28.1, 27.5, 26.7, 26.0, 22.7, 14.1; HRMS (ESI) (m/z): calcd for C_23_H_43_NO^79^Br (M + H)^+^ 428.2523, found 428.2523; calcd for C_23_H_43_NO^81^Br (M + H)^+^ 430.2502, found 430.2502; Calcd for C_23_H_42_NO^79^BrNa (M + Na)^+^ 450.2342, found 450.2341; calcd for C_23_H_42_NO^81^BrNa (M + Na)^+^ 452.2322, found 452.2317.

#### 3.1.11. 3-(10-hydroxydecyl)-5-decylisoxazole (**40**)

To a solution of **39** (0.210 g, 0.49 mmol) in DMSO (1 mL), a solution of K_2_CO_3_ (0.136 g, 0.98 mmol) in H_2_O (2 mL) was added. The mixture was heated at 100 °C for 24 h. After cooling to rt, the mixture was diluted with water, neutralized with 1 N HCl and it was extracted with EtOAc. The organic layer was washed with brine, dried over Na_2_SO_4_ and evaporated to dryness. The residue was subjected to FCC (hex/EtOAc = 9:1–8:2) to furnish compound **40**. Pale-orange solid; mp 54–56 °C; yield: 0.12 g, 66%; ^1^H-NMR (300 MHz, CDCl_3_) *δ* 5.79 (s, 1H), 3.63 (t, *J* = 6.6 Hz), 3.48 (s, 1H), 2.68 (t, *J* = 7.6 Hz, 2H), 2.60 (t, *J* = 7.6 Hz, 2H), 1.73–1.49 (m, 6H), 1.35–1.24 (m, 26H), 0.87 (t, *J* = 6.6 Hz, 3H); ^13^C-NMR (75 MHz, CDCl_3_) *δ* 173.3, 164.2, 100.2, 63.0, 32.8, 31.9, 29.53, 29.5 (2C, 2CH_2_), 29.45, 29.4, 29.28, 29.23, 29.21, 29.2, 29.1, 28.3, 27.5, 26.7, 26.0, 25.7, 22.7, 14.1; HRMS (ESI) (m/z): calcd for C_23_H_44_NO_2_ (M + H)^+^ 366.3367, found 366.3365; calcd for C_23_H_43_NO_2_Na (M + Na)^+^ 388.3186, found 388.3183.

#### 3.1.12. Nonanenitrile (**43**)

To a solution of 1-bromooctane (**42**) (2.5 mL, 14.4 mmol) in DMF (42 mL), NaCN (0.85 g, 17.3 mmol) was added. The resulting mixture was heated at 90 °C. An additional quantity of NaCN (1.41 g, 28.79 mmol) was added after 2 h and the reaction was stirred at the above temperature for 44 h. After cooling to rt, DMF was evaporated under reduced pressure, Et_2_O was added, the organic layer was washed with water, brine, dried over Na_2_SO_4_ and evaporated to dryness to furnish compound 43. Pale-yellow oil; yield: 1.38 g, 69%; ^1^H-NMR (300 MHz, CDCl_3_) δ 2.33 (t, *J* = 7.1 Hz, 2H), 1.70–1.61 (m, 2H), 1.49–1.40 (m, 2H), 1.23–1.36 (m, 8H), 0.88 (t, *J* = 6.6 Hz, 3H); ^13^C-NMR (75 MHz, CDCl_3_) δ 119.8, 31.7, 28.9, 28.8, 28.6, 25.4, 22.6, 17.1, 14.0.

#### 3.1.13. N’-hydroxynonanimidamide (**44**) 

To a solution of hydroxylamine hydrochloride (0.299 g, 4.31 mmol) in ^i^PrOH (4.4 mL), NaHCO_3_ (0.603 g, 7.18 mmol) was added. The resulting mixture was stirred at rt for 15 min. Then, nonanenitrile (**43**) (0.4 g, 2.87 mmol) was added and the reaction mixture was stirred at 80–85 °C for 4 h. Upon completion, it was cooled to rt, filtered and washed with ^i^PrOH. The filtrate was evaporated under vacuo. Pure compound 44 was obtained after FCC (CH_2_Cl_2_/MeOH = 98:2 to 95:5). White solid [52]; mp 79–81 °C; yield: 0.29 g, 59%; ^1^H-NMR (300 MHz, CDCl_3_) *δ* 4.52 (br. s, 2H), 2.13 (t, *J* = 7.5 Hz, 2H), 1.59–1.49 (m, 2H), 1.39–1.20 (m, 10H), 0.87 (t, *J* = 6.7 Hz, 3H); ^13^C-NMR (75 MHz, CDCl_3_) *δ* 154.2, 31.8, 31.3, 29.3, 29.1, 29.09, 26.6, 22.6, 14.1.

#### 3.1.14. General Procedure for the Synthesis of Compounds **47** and **48**

To the suspension of active ester **45** or **46** (2.61 mmol) in PhMe (2.5 mL), placed in a tube (10 mL pressure-rated reaction vial), N’-hydroxynonanimidamide (**44**) (0.3 g, 1.74 mmol) was added. The resulting mixture was irradiated using a CEM microwave reactor for 5 min at 160 °C. After cooling to rt, the mixture was extracted with EtOAc, the organic layer washed with water and brine, dried over Na_2_SO_4_ and evaporated to dryness. Compounds **47** and **48** were obtained after FCC purification (petroleum ether/EtOAc = 96:4).

##### 5-(5-bromopentyl)-3-octyl-1,2,4-oxadiazole (**47**)

It was synthesized according to the general procedure described above from active ester 45. Pale-yellow oil; yield: 0.306 g, 52%; ^1^H-NMR (300 MHz, CDCl_3_) *δ* 3.40 (t, *J* = 6.8 Hz, 2H), 2.84 (t, *J* = 7.6 Hz, 2H), 2.69 (t, *J* = 7.6 Hz, 2H), 1.94–1.65 (m, 6H), 1.50–1.21 (m, 22H), 0.87 (t, *J* = 6.3 Hz, 3H); ^13^C-NMR (75 MHz, CDCl_3_) *δ* 179.2, 170.8, 33.3, 32.3, 31.9, 29.3, 29.28, 29.27, 27.7, 27.1, 26.5, 26.1, 25.9, 22.8, 14.2; HRMS (ESI) (m/z): calcd for C_15_H_28_N_2_O^79^Br (M + H)^+^ 331.1380, found 331.1380; calcd for C_15_H_28_N_2_O^81^Br (M + H)^+^ 333.1355, found 333.1359. 

##### 5-(10-bromodecyl)-3-octyl-1,2,4-oxadiazole (**48**)

It was synthesized according to the general procedure described above from active ester **46**. Pale-yellow oil; yield: 0.227 g, 64%; ^1^H-NMR (300 MHz, CDCl_3_) *δ* 3.40 (t, *J* = 6.8 Hz, 2H), 2.84 (t, *J* = 7.6 Hz, 2H), 2.69 (t, *J* = 7.6 Hz, 2H), 1.94–1.65 (m, 6H), 1.50–1.21 (m, 22H), 0.87 (t, *J* = 6.3 Hz, 3H); ^13^C-NMR (75 MHz, CDCl_3_) *δ* 179.6, 170.5, 34.0, 32.8, 31.8, 29.3, 29.2, 29.15, 29.12, 29.1, 28.99, 28.96, 28.7, 28.1, 27.0, 26.6, 26.5, 26.0, 22.6, 14.1; HRMS (ESI) (m/z): calcd for C_20_H_38_N_2_O^79^Br (M + H)^+^ 401.2168, found 401.2162; calcd for C_20_H_38_N_2_O^81^Br (M + H)^+^ 403.2142, found 403.2141; calcd for C_20_H_37_N_2_O^79^BrNa (M + Na)^+^ 423.1987, found 423.1980; calcd for C_20_H_37_N_2_O^81^BrNa (M + Na)^+^ 425.1961, found 425.1957.

#### 3.1.15. General Procedure for the Synthesis of Compounds **51** and **52**

A solution of **47** or **48** (1 equiv, 1.0 mmol) and CH_3_CO_2_K (4 equiv) in DMF (0.1 M) was heated at 70–80 °C for 4 h. The solvent was removed under reduced pressure, the residue was taken up in EtOAc and washed with water and brine. The organic layer was dried over Na_2_SO_4_ and concentrated in vacuo to afford acetates 49 and 50, which were used to the next step without further purification.

To a solution of 49 or 50 (1 equiv, 1.0 mmol) in THF/MeOH (1:3, 0.05 M), a solution of LiOH (1.3 equiv) in H_2_O (0.2 M) was added dropwise. The mixture was stirred at rt for 2 h. Upon completion, the solvents were removed in vacuo, the residue was taken up in EtOAc and washed with water and brine. The organic layer was dried over Na_2_SO_4_ and concentrated in vacuo. Pure alcohols 51 and 52 were obtained after FCC purification.

##### 5-(3-octyl-1,2,4-oxadiazol-5-yl)pentan-1-ol (**51**)

It was synthesized according to the general procedure described above from bromide **47**. Pale-yellow oil; yield: 0.236 g, 82%; ^1^H-NMR (300 MHz, CDCl_3_) *δ* 3.65 (t, *J* = 6.1 Hz, 2H), 2.86 (t, *J* = 7.5 Hz, 2H), 2.69 (t, *J* = 7.6 Hz, 2H), 1.89–179 (m, 2H), 1.76–1.56 (m, 4H), 1.51–1.44 (m, 2H), 1.38–1.24 (m, 10H), 0.86 (t, *J* = 6.3 Hz); ^13^C-NMR (75 MHz, CDCl_3_) *δ* 179.3, 170.5, 62.4, 32.0, 31.8, 29.13, 29.11, 29.1, 27.0, 26.4, 26.2, 26.0, 25.2, 22.6, 14.1; HRMS (ESI) (m/z): calcd for C_15_H_29_N_2_O_2_ (M + H)^+^ 269.2224, found 269.2220.

##### 10-(3-octyl-1,2,4-oxadiazol-5-yl)decan-1-ol (**52**)

It was synthesized according to the general procedure described above from bromide **48**. Pale-yellow oil; yield: 0.271 g, 80%; ^1^H-NMR (300 MHz, CDCl_3_) *δ* 3.63 (t, *J* = 6.6 Hz, 2H), 2.84 (t, *J* = 7.6 Hz, 2H), 2.69 (t, *J* = 7.5 Hz, 2H), 1.84–1.68 (m, 4H), 1.59–1.50 (m, 2H), 1.40–1.24 (m, 22H), 0.87 (t, *J* = 6.2 Hz); ^13^C-NMR (75 MHz, CDCl_3_) *δ* 179.6, 170.5, 63.0, 32.8, 31.8, 29.4, 29.3, 29.2, 29.15, 29.12, 29.1, 29.0, 28.96, 27.0, 26.6, 26.5, 26.0, 25.7, 22.6, 14.07; HRMS (ESI) (m/z): calcd for C_20_H_39_N_2_O_2_ (M + H)^+^ 339.3006, found 339.3003.

#### 3.1.16. General Procedure for the Synthesis of Compounds **57** and **58**

To a solution of ester **55** or **56** (1 equiv, 1.0 mmol) in absolute ethanol (5.8 M), placed in a tube (10 mL pressure-rated reaction vial), hydrazine monohydrate (4.5 equiv) was added. The resulting mixture was irradiated using a CEM microwave reactor for 17 min at 120 °C. After cooling to rt, the solid was filtered to give compounds **57** and **58**. These compounds were used to the next step without further purification.

##### n-octanoic Acid Hydrazide (**57**) 

It was synthesized according to the general procedure described above from ester 55. White solid [53]; yield: 33%; m.p. 87–89 °C; ^1^H-NMR (300 MHz, CDCl_3_) *δ* 6.68 (br.s, 1H), 3.88 (br.s, 2H), 2.14 (t, *J* = 7.5 Hz, 2H), 1.63 (q, *J* = 7.5 Hz, 2H), 1.36–1.22 (m, 8H), 0.90–0.84 (m, 3H); ^13^C-NMR (75 MHz, CDCl_3_) *δ* 174.3, 34.7, 31.8, 29.3, 29.0, 25.6, 22.7, 14.1; ESI-MS (m/z): 181.01 (M + Na)^+^, 159.17 (M + H)^+^.

##### n-decanoic Acid Hydrazide (**58**) 

It was synthesized according to the general procedure described above from ester 56. White solid [54]; yield 54%; m.p. 92–94 °C; ^1^H-NMR (300 MHz, CDCl_3_) *δ* 6.70 (br.s, 1H), 3.89 (d, *J* = 4.2 Hz, 2H), 2.14 (t, *J* = 7.5 Hz, 2H), 1.62 (q, *J* = 7.5 Hz, 2H), 1.34–1.21 (m, 16H), 0.91–0.84 (m, 3H); ^13^C-NMR (75 MHz, CDCl_3_) *δ* 174.5, 34.7, 32.0, 29.7, 29.6, 29.5, 29.4, 25.6, 22.8, 14.2; ESI-MS (m/z): 209.10 (M + Na)^+^, 187.21 (M + H)^+^. 

#### 3.1.17. General Procedure for the Synthesis of Compounds **59** and **60**

To a rb-flask containing hydrazides **57** or **58** (1 equiv, 1.0 mmol) and **3** (1.2 equiv), POCl_3_ (6.5 equiv) was added and the resulting mixture was refluxed for 2 h. Then, it was cooled to 0 °C, H_2_O was added dropwise and it was extracted with EtOAc. The organic layer washed with water and brine, dried over Na_2_SO_4_ and concentrated under reduced pressure. Pure compounds **59** and **60** were obtained after FCC purification.

##### 2-(10-bromodecyl)-5-heptyl-1,3,4-oxadiazole (**59**)

It was synthesized according to the general procedure described above from hydrazide **57**. Pale-yellow oil; yield: 60%; ^1^H-NMR (300 MHz, CDCl_3_) *δ* 3.40 (t, *J* = 6.8 Hz, 2H), 2.80 (t, *J* = 7.6 Hz, 4H), 1.89–1.71 (m, 6H), 1.44–1.24 (m, 20H), 0.88 (t, *J* = 6.6 Hz, 3H); ^13^C-NMR (75 MHz, CDCl_3_) *δ* 166.94, 166.9, 34.0, 32.8, 31.6, 29.3, 29.2, 29.0 (2C, 2CH_2_), 28.9, 28.7, 28.68, 28.1, 26.46, 26.43, 25.3 (2C, 2CH_2_), 22.5, 14.0; HRMS (ESI) (m/z): calcd for C_19_H_36_N_2_O^79^Br (M + H)^+^ 387.2011, found 387.2005; calcd for C_19_H_36_N_2_O^81^Br (M + H)^+^ 389.1985, found 389.1984; calcd for C_19_H_35_N_2_O^79^BrNa (M + Na)^+^ 409.1830, found 409.1823; calcd for C_19_H_35_N_2_O^81^BrNa (M + Na)^+^ 411.1805, found 411.1799.

##### 2-(10-bromodecyl)-5-undecyl-1,3,4-oxadiazole (**60**)

It was synthesized according to the general procedure described above from hydrazide 58. White solid; m.p. 61–63 °C; yield: 77%; ^1^H-NMR (300 MHz, CDCl_3_) *δ* 3.39 (t, *J* = 6.8 Hz, 2H), 2.79 (t, *J* = 7.6 Hz, 4H), 1.84 (m, 3H), 1.75 (m, 3H), 1.42–1.25 (m, 28H), 0.87 (t, *J* = 6.7 Hz, 3H); ^13^C-NMR (75 MHz, CDCl_3_) *δ* 166.95, 166.89, 34.0, 32.8, 31.9, 29.56, 29.54, 29.5, 29.4, 29.3, 29.2, 29.1, 29.0, 28.97, 28.93, 28.7, 28.1, 26.47, 26.43, 25.31, 25.30, 22.7, 14.1; HRMS (ESI) (m/z): calcd for C_23_H_44_N_2_O^79^Br (M + H)^+^ 443.2637, found 443.2633. Calcd for C_23_H_44_N_2_O^81^Br (M + H)^+^ 445.2611, found 445.2612; calcd for C_23_H_43_N_2_O^79^BrNa (M + Na)^+^ 465.2456, found 465.2450; calcd for C_23_H_43_N_2_O^81^BrNa (M + Na)^+^ 467.2431, found 467.2428.

#### 3.1.18. General Procedure for the Synthesis of **63** and **64**

A solution of **59** or **60** (1 equiv, 1.0 mmol) and CH_3_COOK (4 equiv) in DMF (0.1 M) was heated at 70–80 °C for 3 h. DMF was then removed under reduced pressure. The residue was dissolved in EtOAc and washed with water and brine. The organic layer was dried over Na_2_SO_4_ and concentrated in vacuo to obtain the corresponding acetates **61** and **62,** which were used to the next step without further purification.

To a solution of **61** or **62** (1 equiv, 1.0 mmol) in THF/MeOH (1:3, 0.05 M), a solution of LiOH (1.3 equiv) in H_2_O (0.2 M) was added dropwise. The mixture was stirred at rt for 2–3 h. Upon completion, the solvents were removed in vacuo, the residue was taken up in EtOAc and washed with water and brine. The organic layer was dried over Na_2_SO_4_ and concentrated in vacuo. Pure alcohols **63** and **64** were obtained after FCC purification.

##### 10-(5-heptyl-1,3,4-oxadiazol-2-yl)decan-1-ol (**63**)

It was synthesized according to the general procedure described above from bromide **59**. White solid; m.p. 48–50 °C; yield 75%; ^1^H-NMR (300 MHz, CDCl_3_) *δ* 3.61 (t, *J* = 6.6 Hz, 2H), 2.78 (t, *J* = 7.6 Hz, 4H), 1.79–1.69 (m, 4H), 1.59–1.50 (m, 2H), 1.39–1.24 (m, 20H), 0.86 (t, *J* = 6.7 Hz, 3H); ^13^C-NMR (75 MHz, CDCl_3_) *δ* 166.94, 166.91, 62.9, 32.7, 31.6, 29.4, 29.3, 29.25, 29.0, 28.9, 28.7, 27.8, 26.45, 26.41, 25.7, 25.3 (2C, 2CH_2_), 22.5, 14.0; ESI-MS (m/z): 325.2 (M + H)^+^. HRMS (ESI) (m/z): calcd for C_19_H_37_N_2_O_2_ (M + H)^+^ 325.2855, found 325.2848.

##### 10-(5-undecyl-1,3,4-oxadiazol-2-yl)decan-1-ol (**64**)

White solid; m.p. 68–70 °C; yield: 73%; ^1^H-NMR (300 MHz, CDCl_3_) *δ* 3.63 (q, *J* = 6.1 Hz, 2H), 2.79 (t, *J* = 7.6 Hz, 4H), 1.80–1.70 (m, 4H), 1.59–1.51 (m, 2H), 1.38–1.25 (m, 28H), 0.87 (t, *J* = 6.6 Hz, 3H); ^13^C-NMR (75 MHz, CDCl_3_) *δ* 166.95, 166.91, 63.0, 32.8, 31.9, 29.56, 29.54, 29.4, 29.38, 29.32, 29.3, 29.26, 29.1, 29.0, 28.98, 28.92, 26.5, 26.4, 25.7, 25.31, 25.30, 22.7, 14.1; HRMS (ESI) (m/z): calcd for C_23_H_45_N_2_O_2_ (M + H)^+^ 381.3481, found 381.3473; calcd for C_23_H_44_N_2_O_2_Na (M + Na)^+^ 403.3300, found 403.3288.

#### 3.1.19. General Procedure for the Synthesis of Ether Phospholipids (Method B)

To a cold solution at −5 °C of POCl_3_ (1.3 equiv) in THF (0.2 M), a mixture of the appropriate alcohol (1 equiv, 1 mmol) and Et_3_N (1.8 equiv) in THF (0.15 M) was added dropwise. The reaction mixture was stirred at this temperature until the consumption of alcohol. Then, water was added and stirring was continued for 30 min. The aqueous layer was extracted with EtOAc and then with CH_2_Cl_2_. The combined organic extracts were dried over anhydrous Na_2_SO_4_ and the solvent was evaporated in vacuo to afford the corresponding phosphoric acid derivative, which was converted to its corresponding pyridinium salt upon addition of anhydrous pyridine and stirring for 2.5 h at 45 °C. After cooling, the solvent was evaporated in vacuo and pyridine (5 mL) was added to the residue. To the resulting solution were sequentially added choline *p*-toluenesulfonate (1.5 equiv) and 1-(mesitylene-2-sulfonyl)-3-nitro-1,2,4-triazole (MSNT) (1.8 equiv) at 0 °C and the mixture was stirred at ambient temperature for 48 h. Subsequently, the mixture was hydrolysed by the addition of 2-propanol/H_2_O (7:3). The mixture was stirred for 1 h, the solvent was evaporated in vacuo and the residue was subjected to FCC (CH_2_Cl_2_/MeOH/NH_4_OH = 95:5:0.5–60:40:0.5) to afford the desired phospholipid derivative.

##### 10-(5-decylisoxazol-3-yl)decyl (2-(trimethylammonio)ethyl) phosphate inner salt (**41**)

Compound 41 was prepared according to the general method for the synthesis of ether phospholipid derivatives reported above (Method B) using alcohol 40 (0.120 g, 0.33 mmol). Gummy solid; yield: 0.069 g, 40% yield; ^1^H-NMR (600 MHz, CD_3_OD) *δ* 5.86 (s, 1H), 4.18 (br.s, 2H), 3.80 (dd, *J* = 12.6 and 6.2 Hz, 2H), 3.56 (br.s, 2H), 3.17 (s, 9H), 2.65 (t, *J* = 7.4 Hz, 2H), 2.54 (t, *J* = 7.6 Hz, 2H), 1.63–1.55 (m, 6H), 1.31–1.20 (m, 26H), 0.82 (t, *J* = 6.8 Hz, 3H); ^13^C-NMR (150 MHz, CD_3_OD) *δ* 174.4, 164.9, 101.2, 66.5 (m), 65.7 (d, *J_C,P_* = 65.7 Hz,), 61.8, 58.8 (d, *J_C,P_* = 58.9 Hz), 54.5 (3C, N(CH_3_)_3_), 32.5, 31.4, 31.3, 30.14, 30.1, 30.0, 29.93, 29.9, 29.8, 29.7, 29.6, 28.8, 28.1, 27.1, 26.5, 26.4, 23.2, 14.3; ^31^P-NMR (121 MHz, CD_3_OD) *δ*−0.13; HRMS (ESI) (m/z): calcd for C_28_H_56_N_2_O_5_P (M + H)^+^ 531.3927, found 531.3926; calcd for C_28_H_55_N_2_O_5_PNa (M + Na)+ 553.3746, found 553.3740.

##### 5-(3-octyl-1,2,4-oxadiazol-5-yl)pentyl (2-(trimethylammonio)ethyl) phosphate inner salt (**53**)

Compound **53** was prepared according to the general method for the synthesis of ether phospholipid derivatives reported above (Method B) using alcohol **51** (0.205 mg, 0.764 mmol). Gummy solid; yield: 0.151 g, 46%; ^1^H-NMR (600 MHz, CD_3_OD) *δ* 4.23 (br.s, 2H), 3.87 (q, *J* = 6.4 Hz, 2H), 3.62–3.60 (m, 2H), 3.21 (s, 9H), 2.90 (t, *J* = 7.5 Hz, 2H), 2.67 (t, *J* = 7.5 Hz, 2H), 1.88–1.80 (m, 2H), 1.72–1.65 (m, 4H), 1.51–1.46 (m, 2H), 1.32–1.28 (m, 10H), 0.88 (t, *J* = 6.9 Hz, 3H); ^13^C-NMR (150 MHz, CD_3_OD) *δ* 181.4, 171.6, 67.5 (m), 66.5 (d, *J_C,P_* = 65.1 Hz), 60.3 (d, *J_C,P_* = 58.8 Hz), 54.7, 54.69, 54.64, 33.0, 31.3, 31.25, 30.3, 30.0, 28.0, 27.3, 27.1, 26.6, 26.4, 23.7, 14.4; ^31^P-NMR (121 MHz, CD_3_OD) δ−0.10; HRMS (ESI) (m/z): calcd for C_20_H_41_N_3_O_5_P (M + H)^+^ 434.2784, found 434.2779; calcd for C_20_H_40_N_3_O_5_PNa (M + Na)^+^ 456.2603, found 456.2589.

##### 10-(3-octyl-1,2,4-oxadiazol-5-yl)decyl (2-(trimethylammonio)ethyl) phosphate inner salt (**54**)

Compound **54** was prepared according to the general method for the synthesis of ether phospholipid derivatives reported above (Method B) using alcohol **52** (0.085 g, 0.25 mmol). Gummy solid; yield: 0.04 g, 32%; ^1^H-NMR (600 MHz, CD_3_OD) *δ* 4.25 (br. s, 2H), 3.86 (q, *J* = 6.4 Hz, 2H), 3.65–3.62 (m, 2H), 3.23 (s, 9H), 2.88 (t, *J* = 7.4 Hz, 2H), 2.68 (t, *J* = 7.4 Hz, 2H), 1.80–1.60 (m, 8H), 1.39–1.25 (m, 20H), 0.88 (t, *J* = 6.9 Hz, 3H); ^13^C-NMR (150 MHz, CD_3_OD) *δ* 181.5, 171.5, 67.4 (m), 66.8 (d, *J_C,P_* = 65.5 Hz), 60.2 (d, *J_C,P_* = 58.9 Hz), 54.74, 54.69, 54.64, 32.94, 32.92, 31.8, 30.6, 30.49, 30.42, 30.24, 30.2, 30.0, 29.99, 27.9, 27.6, 27.1, 26.9, 26.6, 23.7, 14.5; ^31^P-NMR (121 MHz, CD_3_OD) *δ*−0.06; HRMS (ESI) (m/z): calcd for C_25_H_51_N_3_O_5_P (M + H)^+^ 504.3561, found 504.3564; calcd for C_25_H_50_N_3_O_5_PNa (M + Na)^+^ 526.3380, found 526.3375.

##### 10-(5-heptyl-1,3,4-oxadiazol-2-yl)decyl (2-(trimethylammonio)ethyl) phosphate inner salt (**65**)

Phospholipid **65** was prepared according to the general method for the synthesis of ether phospholipid derivatives reported above (Method B) using alcohol **63** (0.250 mg, 0.77 mmol). Gummy solid; yield: 0.136 g, 36%; ^1^H-NMR (600 MHz, CD_3_OD) *δ* 4.24 (br. s, 2H), 3.86 (q, *J* = 6.5 Hz, 2H), 3.64–3.61 (m, 2H), 3.22 (s, 9H), 2.84 (t, *J* = 7.4 Hz, 4H), 1.81–1.72 (m, 4H), 1.67–1.58 (m, 2H), 1.40–1.30 (m, 20H), 0.90 (t, *J* = 6.6 Hz, 3H); ^13^C-NMR (150 MHz, CD_3_OD) *δ* 168.97, 168.95, 67.5 (m), 66.8 (d, *J_C,P_* = 65.5 Hz), 60.2 (d, *J_C,P_* = 58.8 Hz), 54.74, 54.69, 54.63, 32.8, 31.9, 31.8, 30.7, 30.5, 30.4, 30.2, 29.97, 29.92, 29.9, 27.4, 26.9 (2C, 2CH_2_), 25.9, 23.6, 14.4; ^31^P-NMR (121 MHz, CD_3_OD) δ−0.04; HRMS (ESI) (m/z): calcd for C_24_H_49_N_3_O_5_P (M + H)^+^ 490.3404, found 490.3406; calcd for C_24_H_48_N_3_O_5_PNa (M + Na)+ 512.3224, found 512.3223.

##### 2-(trimethylammonio)ethyl (10-(5-undecyl-1,3,4-oxadiazol-2-yl)decyl) phosphate inner salt (**66**)

Lipid **66** was prepared according to the general method for the synthesis of ether phospholipid derivatives reported above (Method B) using alcohol 64 (0.2 g, 0.53 mmol). Gummy solid; yield: 0.113 g, 39%; ^1^H-NMR (600 MHz, CD_3_OD) *δ* 4.24 (br. s, 2H), 3.86 (q, *J* = 6.5 Hz, 2H), 3.64–3.61 (m, 2H), 3.22 (s, 9H), 2.84 (t, *J* = 7.4 Hz, 4H), 1.81–1.71 (m, 4H), 1.68–1.58 (m, 2H), 1.40–1.27 (m, 28H), 0.89 (t, *J* = 6.6 Hz, 3H); ^13^C-NMR (150 MHz, CD_3_OD) *δ* 168.95, 168.94, 67.5 (m), 66.8 (d, *J_C,P_* = 65.5 Hz) 60.3 (d, *J_C,P_* = 58.8 Hz), 54.7, 54.68, 54.63, 33.1, 31.9, 31.8, 30.7, 30.68, 30.66, 30.54, 30.51, 30.5, 30.4, 30.21, 30.18, 29.97, 29.94, 27.4, 27.38, 26.92, 25.9, 23.7, 14.5; ^31^P-NMR (121 MHz, CD_3_OD) *δ*−0.04; HRMS (ESI) (m/z): calcd for C_28_H_57_N_3_O_5_P (M + H)^+^ 546.4030, found 546.4035; calcd for C_28_H_56_N_3_O_5_PNa (M + Na)+ 568.3850, found 568.3848. 

### 3.2. Biological Evaluation

#### 3.2.1. Parasites

A cloned line of *L. infantum* (MOM/ MA671TMAP263) promastigotes were maintained in RPMI 1640 medium supplemented with 10% heat-inactivated foetal bovine serum (FBS), 2 mM L-glutamine, 20 mM HEPES and 1% Penn/Strep. Maintenance of promastigotes was done in T-25 flasks at 26 °C by subpassage at 106 parasites/mL every 5−6 days. *L. infantum* axenic amastigotes expressing episomal luciferase were maintained in MAA/20 (axenic amastigote medium) at 37 °C under a 5% CO_2_ environment with subpassages every 5 days. LUC-positive parasites were selected by addition of geneticin sulphate (G418) to the culture at 60 μg/mL.

*L. donovani* strain BPK 190 [55] was cultivated at 25 °C in modified Medium199 (Sigma-Aldrich, with 20**%** heat-inactivated FCS, 40 mM HEPES pH 7.4, 0.2**%** NaHCO3, 100 μM adenin, 1.2 μg/mL 6-biopterin and 10 μg/mL haem, with 1 × Pen/Strep/l-glutamine (Sigma, Saint Louis, MI, USA), pH 7.0).

*Trypanosoma brucei brucei* Lister 427 bloodstream forms were grown at 37 °C, 5% CO_2_ in complete HMI-9 medium supplemented with 10% foetal calf serum (FCS) and 100 UI/mL of penicillin/streptomycin. Cultures were diluted before a cell density of 2 × 106/mL was reached.

*T. cruzi* epimastigotes (Y strain) were obtained by axenic cultivation in the LIT medium, supplemented with 10% FBS (Cultilab, São Paulo, Brazil). Cultures were maintained at 280 °C for 4–5 days until they reached the log growth phase. New cultures were made every 4 days from the initial inoculum of 2 × 106 parasites/mL. *T. cruzi* trypomastigotes (Y strain) were maintained in culture by the infection of LLC-MK2 (ATTC-CCL7) epithelial cells. This cell line was cultured in 175 cm^3^ tissue culture bottles (TPP) in RPMI 1640 medium (Gibco), (Thermo Fischer Scientific, Carlsbad, CA, USA) supplemented with 10% FBS (Gibco) (Thermo Fischer Scientific, Carlsbad, CA, USA). Cultures were maintained at 37 °C under 5% CO_2_ atmosphere, and RPMI 1640 medium and SFB were exchanged every 48 h. After confluence, the cultures were treated with 1 mL of a mixture of trypsin and versene (Sigma, São Paulo, Brazil) (0.2 and 0.002%, respectively). After 24 h, a subconfluence culture was infected with the trypomastigotes in the approximate proportion of 10:1 parasites/cell. From five to seven days post infection, the trypomastigote stage was obtained from the culture supernatant. Alternatively (Figure 2), *T. cruzi* strain Y was maintained as previously described [56].

#### 3.2.2. In-Vitro Evaluation of Activity Against *L. donovani* Intramacrophage Amastigotes

Activity against *L. donovani* strain BPK190 was tested in vitro using bone marrow-derived macrophages [57] as host cells. Infection was established for 24 h prior to the addition of compounds from 10 mM stocks in DMSO. Parasite load was determined 48 h later by quantifying *Leishmania* actin DNA relative to mouse actin DNA using real time qPCR as described [58].

#### 3.2.3. In-Vitro Evaluation of Activity Against *L. infantum* MHOM/TN/80/LEM235 Intramacrophage Amastigotes

THP-1 cells were differentiated with 1 mM retinoic acid (Sigma, Saint Louis, MI, USA) for 3 days at 37 °C and 5% CO_2_. Infection of THP-1 cells with promastigotes was achieved by mixing the THP-1 and the parasite cultures at a ratio of 1:4 and incubation at 37 °C and 5% CO_2_. The next day cells were washed by centrifugation at 400 × *g* for 10 min, the pellet was resuspended in RPMI medium, and the suspension was overlaid with an equal volume of Histopaque 1077 (Invitrogen, Waltham, MA, USA). The free promastigotes were removed by centrifugation at 1000 × *g* for 20 min. The cell layer was washed with PBS twice and resuspended in RPMI at 4 × 105 cells/mL. Infection rate was assessed microscopically by Giemsa staining of the infected THP-1 suspension. An amount of 200 µL of infected THP-1 was plated in a 24-well tissue culture plate (Cellstar®, Greiner) (Sigma-Aldrich Chemie GMBH, Taufkirchen, Germany) and mixed with an equal volume of the appropriate compound concentration (compounds were dissolved in DMSO/ethanol, 50/50 *v*/*v*, to a final concentration of 65 mM and linear 5-fold dilutions ranging from 100 to 0.8 µM were done in the culture medium). Incubation proceeded for 72 h at 37 °C and 5% CO_2_, and the percentage of infected cells was monitored microscopically after Giemsa staining.

#### 3.2.4. In-Vitro Evaluation of Activity Against *L. infantum* MHOM/MA/67/ITMAP-263 Intramacrophage Amastigotes

The efficacy of compounds against *L. infantum* intracellular amastigotes was determined according to the literature [59] with slight modifications. Briefly, 1 × 106 THP-1 cell/mL were seeded in a 96 well flat bottom plate (100 µL/well) and differentiated into macrophages by addition of 20 ng/mL of phorbol-myristate 13-acetate (PMA, Sigma, Saint Louis, MI, USA) for 18 h, followed by replacement with fresh medium for 24 h. Then these cells were infected with luciferase-expressing *L. infantum* axenic amastigotes in a macrophage:amastigotes ratio of 1:10 for 4 h at 37 °C, 5% CO_2_. Non-internalized parasites were washed and compounds were added at different concentrations in a final volume of 100 µL. After 72 h of incubation, the media was substituted by 100 µL of PBS. Then of 25 µL of Glo-lysis buffer from the Steady-Glo Luciferase Assay System (Promega, Madison, WI, USA) was added mixed and incubated for 10 min with agitation at 100 rpm. Finally, 30 µL of the Steady-Glo reagent (Promega, Madison, WI, USA) was then added to the plate and was incubated for 15 min with agitation at 100 rpm and then the content of each well was transferred to white-bottom 96-well plates. Luminescence intensity was read using a Synergy 2 Multi-Mode Reader (Biotek, Winooski, VT, USA). The antileishmanial effect was evaluated by the determination of the IC_50_ value (concentration required to inhibit growth in 50%) and calculated by the non-linear regression analysis using GraphPad Prism version 8.1.1 for Windows (GraphPad Software, San Diego CA, USA).

#### 3.2.5. In-Vitro Evaluation of Activity Against *T. brucei* Bloodstream Forms

The efficacy of compounds against *T. brucei* bloodstream forms was evaluated using a modified resazurin-based assay previously described [60]. Mid-log bloodstream forms were added to an equal volume of serial dilutions of compounds in supplemented complete HMI-9 medium at a final cell density of 5 × 103/mL. Following incubation for 72 h at 37 °C 5% CO_2_, 20 μL of a 0.5 mM resazurin solution was added and plates were incubated for a further 4 h under the same conditions. Fluorescence was measured at 540 nm and 620 nm excitation and emission wavelength, respectively, using a Synergy 2 Multi-Mode Reader (Biotek, Winooski, VT, USA). For pentamidine, the antitrypanosomatid effect was evaluated by the determination of the IC_50_ value (concentration required to inhibit growth in 50%) and calculated by non-linear regression analysis using GraphPad Prism version 8.1.1 for Windows (GraphPad Software, San Diego CA, USA).

#### 3.2.6. In-Vitro Evaluation of Antitrypanosomal Activity

The trypomastigotes were obtained from the supernatant of a previously infected cell line (LLC-MK2). Between 5 and 7 days after infection, protozoa were collected from the supernatants of the infected LLC-MK2 cells and were then incubated with fresh RPMI 1640 medium supplemented with 0.5% FBS, with or without the compounds, for 24 h at 37 °C under a 5% CO_2_ atmosphere. The concentration of the compounds at which 50% of the parasites were lysed (LC50) was calculated by counting the cells in a Neubauer chamber when trypomastigotes were treated with 100 nM, 500 nM, 1.0 µM, 1.5 µM and 3.0 µM. The experiment was performed in duplicate for each of the three different experiments. For the antiproliferative assay involving epimastigotes, 106 parasites/mL were cultivated in LIT medium supplemented with 10% FBS. After 24 h of epimastigote growth, different concentrations of 50 nM to 50 µM were added to the culture and incubated for 120 h at 28 °C. Cells were collected every 24 h for counting in a Neubauer chamber. Two controls were used and consisted of liver infusion broth-tryptose (LIT) supplemented with 10% FBS and LIT added with 0.01% DMSO. To investigate the effect of the compounds on intracellular amastigotes, peritoneal macrophages from Swiss mouse (CF1) plated in 96 wells plates (Costar®, Sigma, São Paulo, Brazil) were incubated for 2 h with *T. cruzi* trypomastigotes at a ratio of 10 parasites to 1 cell. The non-internalized parasites were removed by washing with the RPMI 1640 medium, and the host cells were incubated for 24 h at 37 °C to allow full internalization and differentiation of trypomastigotes to amastigotes. Fresh 10% FBS-RPMI 1640 medium with 1 µg/mL Hoescht 3348 and 1 µg/mL of WGA-FITC (control) or the same solution containing the inhibitors was added to the infected cells, which were then incubated for 96 h at 37 °C. Every 24 **h** samples were taken and analysed in high content equipment (InCell Analyser 2000, GE Healthcare, Chicago, IL, USA) with 20× air objective (NA 0.4) using respectively filters to excitation and emission to DAPI (405/455 nm), FITC (488/505 nm) and Cy3 (514/605 nm). Six images were collected for each well for reliable statistical analysis. No significant differences were observed in cell or amastigote numbers among images in different locations within wells. The infection index (i.e., the percentage of infected host cells multiplied by the average number of intracellular amastigotes per infected host cell) was determined by counting a total of 500 host cells. Inhibitor activity was calculated using the SigmaPlot (version 10) program (Systat Software, Inc., San Jose, CA, USA). The results are expressed as the mean values from three independent experiments. The IC_50_ was calculated for promastigotes and intracellular amastigotes by fitting the values to a non-linear curve analysis. The regression analyses were performed with SigmaPlot 10 (version 10) program (Systat Software, Inc., San Jose, CA, USA).

#### 3.2.7. Cytotoxicity Assessment against THP-1 Macrophages

The effect of compounds selected compounds on THP-1-derived macrophages was assessed by the colorimetric MTT assay (3-(4,5-dimethylthiazol-2-yl)-2,5-diphenyl tetrazolium bromide). Briefly, 1 × 105 THP-1 cells/mL were differentiated into macrophages by addition of 20 ng/mL of phorbol-myristate 13-acetate (PMA, Sigma, Saint Louis, MI, USA) for 18 h, followed by replacement with fresh medium for 24 h. Cells were incubated with 100 µL of compounds ranging from 100 to 1 µM after dilution in the RPMI complete medium containing a maximum amount of 1% DMSO. After incubation for 72 h, the medium was removed and 100 µL of 0.5 mg/mL MTT solution diluted in RPMI was added. Plates were incubated for an additional 4 h to allow viable cells to convert MTT into a purple formazan product. Solubilization of formazan crystals was achieved by the addition of 2-propanol and absorbance was read at 570 nm using a Synergy 2 Multi-Mode Reader (Biotek, Winooski, VT, USA). Cytotoxicity was evaluated by the determination of the CC50 value (drug concentration that reduced the percentage of viable cells in 50**%**) and calculated by non-linear regression analysis using GraphPad Prism version 8.1.1 for Windows (GraphPad Software, San Diego, CA, USA).

#### 3.2.8. Early ADMET Profiling

*Cytotoxicity assay:* A549 cells and WI-38 cells were grown on surface-modified T175 cell culture flasks in Dulbecco’s modified Eagle medium with 10% foetal calf serum (FCS), streptomycin (100 μg/mL) and 100 U/mL penicillin G. At about 80% confluency, cells were washed, trypsinized, resuspended and counted in RPMI-1640 medium before seeding (in triplicate) into white 384-well microtiter plates (20 μL) at 500 cells/well and incubated at 37 °C in the presence of 5% CO_2_ for 24 h. A total of 20 μL/well of CellTiter-Glo (CTG) reagent (Promega Corp., Madison, WI, USA) was added to each well, and plates were read using an EnVision Multilabel 2103 Reader after a 10-min incubation in the dark. Dose–response assays for compounds made use of 11-point curves. Each test compound (200 nL of 10 mM top concentration in 100% *v*/*v* DMSO) was added to cells seeded in polystyrene 384-well cell culture microtiter plates using the Echo 550 Liquid Handler and read after 24 h of incubation at 37 °C in the presence of 5% CO_2_ using CTG as described above. The compounds/positive control (paclitaxel with final concentration of 10 μM and 1% *v*/*v* DMSO) and no compound (final 1% *v*/*v* DMSO) were added into the 384-well plates (200 nL/well; 1% *v*/*v* DMSO) using the Echo 550 Liquid Handler. The raw luminescence signal of each sample was converted into the percentage of cell growth.

*Cytochrome (CYP) P450 inhibition assay:* These assays made use of microsomal preparations of CYP450 (1A2, 2C9, 2C19, 2D6 and 3A4) from baculovirus-infected insect cells (Corning Inc., Corning, NY, USA) and cytochrome c reductase (and cytochrome b5 for CYP450 3A4). For detection of CYP450 activity, the luminescence-based P450-Glo (Promega Corp., Madison, WI, USA) assay system was used that contained a luminogenic CYP450 substrate, lyophilized luciferin detection reagent and reconstitution buffer. The substrates were luciferin derivatives of CYP450-specific substrates that produce (4S)-4,5-dihydro-2-(6-hydroxybenzothiazolyl)-4-thiazolecarboxylic acid (D-luciferin) after cleavage by CYP450 (CYP450 3A4, luciferin-IPA; CYP450 2C19, luciferin-H EGE; CYP450 2C9, luciferin-H; CYP450 2D6, luciferin-ME EGE; CYP450 1A2, luciferin-1A2). CYP450 reactions were initiated by addition of the NADPH regeneration system to the enzyme–substrate mixture with the luciferin detection reagent stopping the reaction and the D-luciferin being converted to oxyluciferin under the production of light being proportional to the CYP450 activity. The CYP450 assays were performed using the Tecan Fluent liquid-handling automation platform (Tecan Group Ltd., Mannedorf, Switzerland) in the 384-well assay format. Compounds were added into an empty 384-well plate (100 nL/well in 1% *v*/*v* DMSO) using the Echo 550 Liquid Handler followed by the addition of 5 μL/well of the CYP450/substrate mixture and incubation for 30 min at 37 °C, after which the reaction was initiated by the addition of the 5 μL/well NADPH regeneration system. After a further 30-min incubation at 37 °C, the CYP450 reaction was stopped and the luciferase reaction was simultaneously initiated by addition of 10 μL/well of luciferin detection reagent, followed by an additional 30-min incubation at 37 °C. The luminescence signal was detected using an Infinite M1000 PRO plate reader (Tecan Group Ltd., Mannedorf, Switzerland). The NCs yielded 0% inhibition (1% *v*/*v* DMSO) and standard CYP450 specific inhibitors were used as positive controls, yielding 100% inhibition (CYP450 1A2, alpha-naphthoflavone; CYP450 2C9, sulfaphenazole; CYP450 2C19, troglitazone; CYP450 2D6, quinidine; CYP450 3A4, ketoconazole).

*Aurora B kinase assay:* Inhibition of Aurora B kinase was determined using the ADP-Glo Kinase Enzyme System (Promega Corp., Madison, WI, USA). The positive control was SU6656 at a final concentration of 1 μM, with the NC being DMSO at the same concentration (*v*/*v*). An enzyme master mix containing 1 × buffer, 50 μM DTT and 17.5 ng/μL (35 μL/well) Aurora B (all reagents provided in the kit) was prepared. A substrate master mix containing 1 × buffer, 36 μM adenosine triphosphate (ATP) and 7.5 ng/μL (15 ng/well) myelin basic protein (MBP) as a substrate (buffer and MBP were provided in the Aurora B Kinase Enzyme System; ultrapure ATP was provided in the ADP-Glo Kinase Assay System) was prepared. Two microliters of the enzyme master mix and 2 μL of the substrate master mix were added to each well of a 384-well low-volume plate. The plate was sealed using Thermowell sealing tape (Corning Inc., Corning, NY, USA) and incubated for 45 min at rt. The enzymatic reaction was stopped by adding 4 μL of ADP-Glo reagent (provided in the ADP-Glo Kinase Assay System) and the plate sealed using Thermowell sealing tape and incubated for 40 min at rt. Following this, 8 μL of detection reagent was added to each well, and the plate was sealed again and incubated for 45 min at rt, with the luminescence measured using the EnVision Multilabel 2103 Reader (PerkinElmer, Waltham, MA. USA). DMSO concentration was tolerated up to 2% *v*/*v* final.

*hERG cardiotoxicity assay:* The Predictor® hERG fluorescence polarization assay (Thermo Fischer Scientific, Waltham, MA, USA) was used to test compounds for potential cardiotoxicity. To each well of the assay plate, 100 nL of the test/control compound was added followed by addition of 5 μL homogenized membrane solution (undiluted) and 5 μL of tracer (1 nM final concentration in the assay). The plates were incubated for 2 h at 25 °C in a humidity controlled incubator, and the fluorescence polarization was measured using an EnVision Multilabel 2103 Reader (PerkinElmer, Waltham, MA. USA). The NCs (0% inhibition) and positive controls with E-4031, a blocker of hERG-type potassium channels (yielding 100% inhibition), were used to normalize the raw data.

*Mitochondrial toxicity assay:* This assay made use of the MitoTracker Red CMXRos dye (Thermo Fischer Scientific, Waltham, MA, USA), which stains the mitochondria in live cells and its accumulation is dependent on the presence of a membrane potential. The renal carcinoma 786-0 cell line was used for mitochondrial toxicity screening. Cells were harvested from a 75 cm^2^ flask at 80% confluency by washing once with 5 mL of rt PBS and incubating with 1 mL of 0.05% *v*/*v* trypsin/0.02% *v*/*v* EDTA for 3 min. Cells were suspended in 10 mL of prewarmed cell culture media (RPMI-1 640 supplemented with 10% *v*/*v* FCS, 100 U/mL penicillin and 100 μg/mL streptomycin) and counted using a Scepter (Merck Millipore, Darmstadt, Germany). The 786-0 cells were diluted to 75,000 cells/mL and 20 μL of this suspension added to each well of a 384-well plate. Cells were incubated for 36 h at 37 °C and 5% CO_2_, and compounds were added using a predilution plate. The positive control was valinomycin at a final concentration of 1 μM, with the negative control being DMSO at the same concentration. Ten microliters of compounds and controls were added to cells and incubated for 6 h at 37 °C and 5% CO_2_ in a humidity controlled atmosphere. After incubation, 10 μL of a 200 nM solution of MitoTracker Red CMXRos in prewarmed cell culture media was added to each well, and the 786-0 cells were incubated for an additional 45 min at 37 °C and 5% CO_2_. MitoTracker Red CMXRos uptake was measured using an Opera Imaging System. To facilitate automatic image analysis, the layout containing the compound area and the valinomycin and DMSO control areas were created and stored. A sublayout of five evenly dispersed fields per well were used. These settings also included a measurement height of 1 μm, which was stored in an exposure file format. By using the stored settings and files, an automated run was repeatedly created and executed. The images were transferred to a file server and uploaded into Columbus 2.4.0 (PerkinElmer, Waltham, MA. USA) using the built-in helper function and analysed therein.

*Data analysis:* The screening data were obtained in triplicate and analysed using ActivityBase (IDBS, Guildford, UK), and outlier elimination in the control wells was performed using the 3-sigma method. Unless stated, dose–response experiments were performed in the 11-point format with the IC_50_ value, Hill slope, minimum signal and maximum signal for each dose–response curve obtained using a four-parameter logistic fit in the XE module of ActivityBase (IDBS).

#### 3.2.9. Electron Microscopy

##### Scanning Electron Microscopy

Control and treated epimastigotes (5 μM of 27–72 h) and trypomastigotes (1 μM of 27–24 h) were washed and then fixed in a solution containing 2.5% glutaraldehyde in 0.1 M cacodylate buffer (pH 7.2) for 1 h at room temperature. After fixation, cells were washed with 0.1 M cacodylate buffer (pH 7.2) and postfixed in a solution containing 1.25% K_4_[Fe(CN)_6_], 1% OsO_4_, 5 mM CaCl_2_ and 0.1 M cacodylate buffer (pH 7.2) for 30 min. After that, cells were washed again in 0.1 M cacodylate buffer (pH 7.2), dehydrated in an ethanol series (30, 50, 70, 90 and 100%), critical point-dried in a Baltec CPD 030 apparatus (Bal- Tec A.G., Balzers, Leichtenstein), and mounted on specimen stubs. The samples were ion sputtered with a 10 nm gold layer to avoid a charge effect and observed with a JSM 5310 JEOL operating at 20 or 25 kV.

##### Transmission Electron Microscopy

Epimastigotes and trypomastigotes were cultivated and treated as previously described (treatment with 0.5 μM-72 h and 1 μM-24 h of 27 for epimastigotes and trypomastigotes, respectively). Intracellular amastigotes were allowed to grow inside peritoneal macrophages platted in 60 mm^2^ Petri dishes (TPP, Trasadingen, Switzerland) and treated for 72 h with 0.25 μM of 27. After the experimental procedure, the cells were washed and then fixed and post fixed as described above for SEM; then, they were dehydrated in increasing concentrations of acetone and embedded in Epon. Ultrathin sections were stained with uranyl acetate and lead citrate and observed under a Jeol 1200EX (Jeol Tokyo, Japan) or Zeiss EM900 (Carl Zeiss A.G., Jena, Germany) transmission electron microscope.

#### 3.2.10. Flow Cytometry and Fluorescence Microscopy for Apoptosis Detection

All epimastigotes of *T. cruzi* (i.e., those treated with compound 27 0.5 µM for 24 or 48 h) were washed, suspended in Annexin V binding buffer and were incubated at room temperature for 15 min with Annexin V-Alexa 488 (Molecular Probes, Eugene, OR, USA) at the concentration indicated by the manufacturer (BD Bioscience, San Jose, CA, USA). At the moment of acquisition, 10 µg/mL of propidium iodide (Molecular Probes, Eugene, OR, USA) was added to the samples. Data were collected by Cellquest Pro**^®^** in a BD FACSCalibur**^®^** and analysed using Summit 4.3 (Dako Colorado Inc., Fort Collins, CO, USA). Ten thousand gated events were harvested from each sample. The control consisted of epimastigotes permeabilized with 0.1% Triton X100 and incubated with 10 µg/mL propidium iodide and/or Annexin V. Two independent experiments and 10,000 events were analysed for each culture condition.

## 4. Conclusions

In summary, 5-membered heterocyclic rings were introduced on the miltefosine scaffold to investigate their effect on antiparasitic activity and toxicity. In detail, seventeen novel ether phospholipid analogues, containing 1,2,3-triazolyl, isoxazolyl, 1,3,4-oxadiazolyl and 1,2,4-oxadiazolyl in the lipid portion, were synthesized. The library was evaluated for their in vitro antiparasitic activity against *L. infantum* and *L. donovani* intracellular amastigotes, against *T. b. brucei* and against *T. cruzi* different developmental stages. The nature of the substituents of the heterocyclic ring (tail) and the oligomethylene spacer between the head group and the heterocyclic ring strongly affected the activity and toxicity of these compounds. The evaluation of the early toxicological profile of the new derivatives pointed out the overall safe profile for the potent compounds.

Our study pinpointed compound **27**, a 1,2,3-triazole derivative substituted by a decyl tail, an undecyl spacer and a choline head group, as the most promising derivative of the series. **27** exhibited a broad spectrum antiparasitic activity, showing submicromolar IC_50_ against the intracellular amastigotes of two *Leishmania infantum* strains and *T. cruzi* Y strain epimastigotes, intracellular amastigotes and trypomastigotes. The compound cytotoxicity against THP-1 macrophages ranged between 50 < CC_50_ < 100 μM. Thus, the click phospholipid **27** showed a lower toxicity compared to miltefosine and a selectivity index >10. Replacement of the 1,2,3-triazole moiety by isoxazolyl, 1,3,4-oxadiazolyl or 1,2,4-oxadiazolyl rings resulted in some cases to more potent compounds accompanied however, by toxicity against THP-1 macrophages. This extensive SAR study paves the way for the understanding of the chemical features required for the development of an optimized miltefosine analogue.

## Figures and Tables

**Table 1:** In vitro evaluation of antiparasitic activity against *L. infantum* (MHOM/MA/67/ITMAP-263) intracellular amastigotes, cytotoxicity against PMA-differentiated THP-1 cells and selectivity index.

	*L. infantum* (MHOM/MA/67/ITMAP-263) Intracellular Amastigotes		Toxicity CC_50_ ± SD Or CC_50_ Interval Estimation (μM)	Selectivity Index CC_50_/IC_50_
Compound	Mean % Inhibition ± SD at 10μM or (1 μM)	IC_50_ ± SD (μM)		
**25**	32 ± 19	34.3 ± 19.4	>100	>3
**26**	44 ± 17		>12.5	
**27**	78 ± 21	4.23 ± 0.94	50–100	11.8–23.6
**28**	N.A.		>100	
**29**	43 ± 1		>100	
**30**	N.A.		>100	
**31**	N.A.		>100	
**32**	5 ± 18		>100	
**33**	12 ± 5		> 10	
**34**	10 ± 9		50–100	
**35**	N.A.		>100	
**36**	N.A.		>100	
**41**	93.7 ± 10		12.5–25	
**53**	98.4 ± 1 (34.6 ± 19)		12.5–25	
**54**	100.4 ± 1 (67.1 ± 11)	0.8 ± 0.18	10.85	13.6
**65**	92.9 ± 5 (4.8 ± 25)		12.5–25	
**66**	94.2 ± 3 (3.3 ± 20)		25–50	
Miltefosine		3.2 ± 1.4	15.9 ± 1.2	4.9

**Table 2:** In vitro evaluation of the antiparasitic activity against *T. cruzi* epimastigotes (Y strain) after incubation for 24 h, 48 h or 72 h.

Compound	IC_50_ (μM) 24 h	IC_50_ (μM) 48 h	IC_50_ (μM) 72 h
**25**	7.8	3.85	2.16
**27**	1.27	0.89	0.67
**29**	>10	>10	>10
Miltefosine	18.4 ± 1.2	9.5 ± 0.9	8.34 ± 0.0

**Table 3:** In vitro evaluation of the antiparasitic activity against *T. cruzi* intracellular amastigotes (Y strain) after incubation for 24 h, 48 h or 72 h.

Compound	IC_50_ (μM) 24 h	IC_50_ (μM) 48 h	IC_50_ (μM) 72 h
**27**	0.1	0.087	0.057
Miltefosine	12.4 ± 0.6	4.59 ± 0.1	4.3 ± 0.12

## Data Availability

Data of the compounds are available from the authors.

## Supplementary Materials

The following are available online. Table S1: In vitro evaluation of antiparasitic activity against *L. Infantum* in tricellular amastigotes, Table S2: In vitro evaluation of toxicities of compounds at 10 μM and miltefosine (at 1 or 10 μΜ), Table S3: In vitro evaluation of antiparasitic activity against the *T. brucei* L427 WT bloodstream form. In addition, copies of ^1^H, ^13^C and ^31^P NMR of the final compounds are included, Figure S1. ^1^H-NMR of compound 25 in CDCl_3_ at 600 MHz, Figure S2. ^13^C-NMR of compound 25 in CDCl_3_ at 150 MHz, Figure S3. ^31^P-NMR of compound 25 in CDCl_3_ at 121.44 MHz, Figure S4. ^1^H-NMR of compound 26 in CDCl_3_ at 300 MHz, Figure S5. ^13^C-NMR of compound 26 in CDCl_3_ at 75 MHz, Figure S6. ^31^P-NMR of compound 25 in CDCl_3_ at 121.44 MHz, Figure S7. ^1^H-NMR of compound 27 in CD_3_OD at 600 MHz, Figure S8. ^13^C-NMR of compound 27 in CD_3_OD at 150 MHz, Figure S9. ^31^P-NMR of compound 27 in CD_3_OD at 121.44 MHz, Figure S10. ^1^H-NMR of compound 28 in CDCl_3_ at 600 MHz, Figure S11. ^13^C-NMR of compound 28 in CDCl_3_ at 150 MHz, Figure S12. ^31^P-NMR of compound 28 in CDCl_3_ at 121.44 MHz, Figure S13. ^1^H-NMR of compound 29 in CD_3_OD at 600 MHz, Figure S14. ^13^C-NMR of compound 29 in CD_3_OD at 150 MHz, Figure S15. ^31^P-NMR of compound 29 in CD_3_OD at 121.44 MHz, Figure S16. ^1^H-NMR of compound 30 in CD_3_OD at 600 MHz, Figure S17. ^13^C-NMR of compound 30 in CD_3_OD at 150 MHz, Figure S18. ^31^P-NMR of compound 30 in CD_3_OD at 121.44 MHz, Figure S19. ^1^H-NMR of compound 31 in CDCl_3_ at 600 MHz, Figure S20. ^13^C-NMR of compound 31 in CD_3_OD at 150 MHz, Figure S21. ^31^P-NMR of compound 31 in CD_3_OD at 121.44 MHz, Figure S22. ^1^H-NMR of compound 32 in CD_3_OD at 600 MHz, Figure S23. ^13^C-NMR of compound 32 in CD_3_OD at 150 MHz, Figure S24. ^31^P-NMR of compound 32 in CD_3_OD at 121.44 MHz, Figure S25. ^1^H-NMR of compound 33 in CDCl_3_ at 600 MHz, Figure S26. ^13^C-NMR of compound 33 in CDCl_3_ at 150 MHz, Figure S27. ^31^P-NMR of compound 33 in CDCl_3_ at 121.44 MHz, Figure S28. ^1^H-NMR of compound 34 in CD_3_OD at 600 MHz, Figure S29. ^13^C-NMR of compound 34 in CD_3_OD at 150 MHz, Figure S30. ^31^P-NMR of compound 34 in CD_3_OD at 121.44 MHz, Figure S31. ^1^H-NMR of compound 35 in CD_3_OD at 600 MHz, Figure S32. ^13^C-NMR of compound 35 in CD_3_OD at 150 MHz, Figure S33. ^31^P-NMR of compound 35 in CD_3_OD at 121.44 MHz, Figure S34. ^1^H-NMR of compound 36 in CD_3_OD at 600 MHz, Figure S35. ^13^C-NMR of compound 36 in CD_3_OD at 150 MHz, Figure S36. ^31^P-NMR of compound 36 in CD_3_OD at 121.44 MHz, Figure S37. ^1^H-NMR of compound 41 in CD_3_OD at 600 MHz, Figure S38. ^13^C-NMR of compound 41 in CD_3_OD at 150 MHz, Figure S39. ^31^P-NMR of compound 41 in CD_3_OD at 121.44 MHz, Figure S40. ^1^H-NMR of compound 53 in CD_3_OD at 600 MHz, Figure S41. ^13^C-NMR of compound 53 in CD_3_OD at 150 MHz, Figure S42. ^31^P-NMR of compound 53 in CD_3_OD at 121.44 MHz, Figure S43. ^1^H-NMR of compound 54 in CD_3_OD at 600 MHz, Figure S44. ^13^C-NMR of compound 54 in CD_3_OD at 150 MHz, Figure S45. ^31^P-NMR of compound 54 in CD_3_OD at 121.44 MHz, Figure S46. ^1^H-NMR of compound 65 in CD_3_OD at 600 MHz, Figure S47. ^13^C-NMR of compound 65 in CD_3_OD at 150 MHz, Figure S48. ^31^P-NMR of compound 65 in CD_3_OD at 121.44 MHz, Figure S49. ^1^H-NMR of compound 66 in CD_3_OD at 600 MHz, Figure S50. ^13^C-NMR of compound 66 in CD_3_OD at 150 MHz, Figure S51. ^31^P-NMR of compound, 66 in CD_3_OD at 121.44 MHz.

## Sample Availability

Samples of the compounds are available from the corresponding author.
