# Supplementary material for: Design, Synthesis and Antiparasitic Evaluation of Click Phospholipids
Source: Molecules. 2021 Jul 10;26(14):4204. doi: 10.3390/molecules26144204 (PMC8305768; doi:10.3390/molecules26144204)
Supplement: Supplementary file 1 [file molecules-26-04204-s001.zip › molecules-1263642-supplementary.pdf]

molecules
MDPI

Supplementary Material

Design, Synthesis and Antiparasitic Evaluation of Click Phospholipids

George E. Magoulas [1,†], Pantelis Afroudakis [1,†], Kalliopi Georgikopoulou [1], Marina Roussaki [1], Chiara Borsari [2], Theano Fotopoulou [1], Nuno Santarem [3,4], Emile Barrias [5,6], Paloma Tejera Nevado [7], Julia Hachenberg [7], Eugenia Bifeld [7], Bernhard Ellinger [8,9], Maria Kuzikov [8,9], Irini Fragiadaki [10], Effie Scoulica [10], Joachim Clos [7], Sheraz Gul [8,9], Maria Paola Costi [11], Wanderley de Souza [5,6], Kyriakos C. Prousis [1], Anabela Cordeiro da Silva [3,4,12] and Theodora Calogeropoulou [1,*]

[1] National Hellenic Research Foundation, Institute of Chemical Biology, 11653 Athens, Greece; gmagoulas@eie.gr (G.E.M.); pafroudakischem@yahoo.gr (P.A.); k.georgikopoulou@access-one.gr (K.G.); mroussaki@eie.gr (M.R.); tfotop@eie.gr (T.F.); kyrprous@eie.gr (K.C.P.)
[2] Department of Biomedicine, University of Basel, 4058 Basel, Switzerland; chiara.borsari@unibas.ch
[3] i3S–Instituto de Investigação e Inovação em Saúde, Universidade do Porto, 4200 -135 Porto, Portugal; santarem@ibmc.up.pt (N.S.); cordeiro@ibmc.up.pt (A.C.d.S.)
[4] Parasite Disease Group, IBMC-Instituto de Biologia Molecular e Celular, 4150-180 Porto, Portugal
[5] Instituto Nacional de Ciência e Tecnologia em Biologia Estrutural e Bioimagens, Universidade Federal do Rio de Janeiro, 21941-902 Rio de Janeiro, Brazil; emilebarrias@gmail.com (E.B.); wsouza@biof.ufrj.br (W.d.S.)
[6] Instituto de Biofísica Carlos Chagas Filho, Universidade Federal do Rio de Janeiro, Ilha do Fundão, 21941-902 Rio Janeiro, Brazil
[7] Bernhard Nocht Institute for Tropical Medicine, D-20359 Hamburg, Germany; paloma.tejera.nevado@gu.se (P.T.N.); Julia.Hachenberg@amedes-group.com (J.H.); bifeld@bnitm.de (E.B.); clos@bni-hamburg.de (J.C.)
[8] Fraunhofer Institute for Translational Medicine and Pharmacology ITMP, 22525 Hamburg, Germany; Bernhard.Ellinger@itmp.fraunhofer.de (B.E.); Maria.Kuzikov@itmp.fraunhofer.de (M.K.); Sheraz.Gul@itmp.fraunhofer.de (S.G.)
[9] Fraunhofer Cluster of Excellence for Immune-Mediated Diseases CIMD, 22525 Hamburg, Germany
[10] Department of Clinical Microbiology and Microbial Pathogenesis, Faculty of Medicine, University of Crete, 70013 Heraklion, Crete, Greece; fragiada@med.uoc.gr (I.F.); e.scoulica@uoc.gr (E.S.)
[11] Department of Pharmacy, Università degli Studi di Modena e Reggio Emilia, 41125 Modena, Italy; costimp@unimore.it
[12] Departmento de Ciências Biológicas, Faculdade de Farmácia, Universidade do Porto, 4099-002 Porto, Portugal
* Correspondence: tcalog@eie.gr; Tel.: +30-2107273833
† These authors have contributed equally to this manuscript.

Table of Contents

1. Supplementary Tables S2

Table S1. *In vitro* evaluation of antiparasitic activity against *L. infantum* (MHOM/TN/80/LEM235) in-tracellular amastigotes. S2

Table S2. *In vitro* evaluation of toxicities (hERG, CYP1A2, CYP2C9, CYP2C19, CYP2D6, CYP3A4 and A549 and WI-38 cytotoxicities) of compounds at 10 μM and Miltefosine (10 μM or 1 μM). S3

Table S3. *In vitro* evaluation of antiparasitic activity against *T. brucei* L427 WT blood-stream form.......... S3

2. Copies of ^{1}H, ^{13}C and ^{31}P NMR of final products S5

1. Supplementary Tables

Table S1. *In vitro* evaluation of antiparasitic activity against *L. infantum* (MHOM/TN/80/LEM235) in-tracellular amastigotes.

Compound	*L. infantum* *MHOM/TN/80/LEM235* intracellular amastigotes	ToxicityCC$_{50}$±SDorCC$_{50}$ in-terval estimation (μM)	Selectivity Index CC$_{50}$/IC$_{50}$
	IC$_{50}$ ± SD (μM)		
**25**	9.33 ± 0.99	>100	10.7
**26**	5.21 ± 0.95	>12.5	2.4
**27**	1.54 ± 0.22	50–100	32.5–64.9
**28**	1.5 ± 0.4	>100	66.7
**29**	0.5 ± 0.2	>100	200
**Miltefosine**	6.68±0.96	15.9 ± 1.2	4.9

Table S2. *In vitro* evaluation of toxicities (hERG, CYP1A2, CYP2C9, CYP2C19, CYP2D6, CYP3A4 and A549 and WI-38 cytotoxicities) of compounds at 10 μM and Miltefosine (10 μM or 1 μM). N.T. = Not Tested.

c	% inhibition ± SD at 10 μM						% cell growth ± SD at 10 μM		% toxicity ± SD at 10 μM	% inhibition ± SD at 10 μM
**Comp.**	***h*ERG**	**CYP1A2**	**CYP2C9**	**CYP2C19**	**CYP2D6**	**CYP3A4**	**A549**	**W1-38**	**Mito-chondria**	**Aurora B**
**25**	–7.70 ± 10.95	–20.41 ± 7.94	–23.11 ± 4.90	–32.10 ± 3.43	–37.71 ± 9.99	–20.13 ± 4.31	114.88 ± 2.04	93.46 ± 9.11	11.05 ± 4.92	–10.28 ± 19.80
**28**	–3.64 ± 5.93	–13.84 ± 9.34	–23.46 ± 6.53	–32.71 ± 4.81	–56.57 ± 9.98	–33.70 ± 4.40	121.51 ± 2.96	114.04 ± 25.66	8.99 ± 15.76	–11.71 ± 9.17
**29**	–4.90 ± 5.58	–28.56 ± 5.97	–22.63 ± 6.19	–28.74 ± 4.48	–46.97 ± 17.56	–33.59 ± 4.80	120.63 ± 0.86	92.93 ± 15.18	1.08 ± 2.29	–6.17 ± 7.07
**30**	N.T.	–2.91 ± 3.00	85.98 ± 0.94	74.33 ± 2.72	50.77 ± 3.64	42.47 ± 8.94	N.T.	N.T.	N.T.	N.T.
**31**	–6.43 ± 13.73	13.80 ± 0.84	8.95 ± 1.08	–2.38 ± 6.77	11.06 ± 1.05	–7.33 ± 3.74	108.64 ± 5.73	123.63 ± 6.26	5.07 ± 4.57	N.T.
**32**	N.T.	12.02 ± 4.19	93.19 ± 0.78	54.87 ± 3.86	83.96 ± 2.45	60.74 ± 6.21	N.T.	N.T.	N.T.	N.T.
**33**	16.60 ± 2.64	15.23 ± 4.45	19.72 ± 2.68	–4.22±4.90	9.87±6.48	7.61 ± 9.09	114.14 ± 9.60	136.32 ± 19.05	-1 ± 4.35	N.T.
**34**	N.T.	–3.73 ± 3.31	18.94 ± 3.85	4.89 ± 11.86	23.08 ± 13.73	19.27 ± 3.43	N.T.	N.T.	N.T.	N.T.
**35**	0.10 ± 4.10	9.42 ± 1.87	2.57 ± 0.76	–3.09 ± 5.40	7.26 ± 1.30	–8.64 ± 5.66	100.96 ± 6.64	119.10 ± 5.69	6.58 ± 11.15	N.T.
**36**	N.T.	3.18 ± 2.49	67.54 ± 2.08	35.51 ± 4.84	58.33 ± 2.06	35.63 ± 5.11	N.T.	N.T.	N.T.	N.T.
**41**	48.78 ± 4.00	10.86 ± 0.71	41.31 ± 8.34	5.65 ± 5.95	45.83 ± 14.78	53.97 ± 5.39	85.15 ± 1.43	54.16 ± 12.05	2.99 ± 9.19	N.T.
**53**	8.95 ± 6.41	20.03 ± 0.84	5.45 ± 6.65	–17.22 ± 1.84	–26.41 ± 6.04	–12.40 ± 4.93	87.74 ± 2.23	83.83 ± 5.08	–1.08 ± 12.96	N.T.
**54**	17.93 ± 5.86	9.37 ± 0.58	37.98 ± 3.38	10.43 ± 8.37	6.07 ± 11.09	13.88 ± 0.37	85.82 ± 2.06	31.39 ± 17.44	8.56 ± 12.10	N.T.
**65**	8.64 ± 8.38	18.05 ± 4.21	28.32 ± 5.98	–1.75 ± 2.75	–30.44 ± 9.48	–2.44 ± 12.03	90.48 ± 5.75	77.74 ± 11.47	40.25 ± 5.67	N.T.
**66**	22.98 ± 9.56	12.37 ± 2.06	53.41 ± 2.53	23.97 ± 7.01	53.99 ± 2.89	59.34 ± 5.31	90.83 ± 8.30	49.49 ± 17.33	19.78 ± 15.77	N.T.
**Miltefosine**	–16.92 ± 7.34	–20.62 ± 11.85	–0.80 ± 38.33	7.92 ± 15.50	30.83 ± 16.70	8.39 ± 8.99	95.80 ± 15.78	113.50 ± 26.66	3.99 ± 8.95	–12.54 ± 10.71

Table S3. *In vitro* evaluation of antiparasitic activity against *T. brucei* L427 WT blood-stream form.

	***T.b. brucei* L427 WT Blood-Stream**	
**Comp.**	**Single Dose Assay 10μM Mean Inhibitory Activity (%) ± SD**	**Dose Response Curves IC_{50} ± SD (nM)**
**25**	N.A	
**26**	N.A	
**27**	N.A	

**28**	N.A.	
**29**	N.A	
**30**	N.A	
**31**	20 ± 1	
**32**	36 ± 4	
**33**	23 ± 3	
**34**	N.A	
**35**	N.A	
**36**	30 ± 7	
**53**	N.A	
**54**	N.A	
**65**	N.A	
**66**	N.A	
**Pentamidine**		1.55 ± 0.24

Average activities of at least 2 independent assays, N.A. No significant activity (less than 20%).

1. Copies of ^{1}H, ^{13}C and ^{31}P NMR of final products

Compound 25

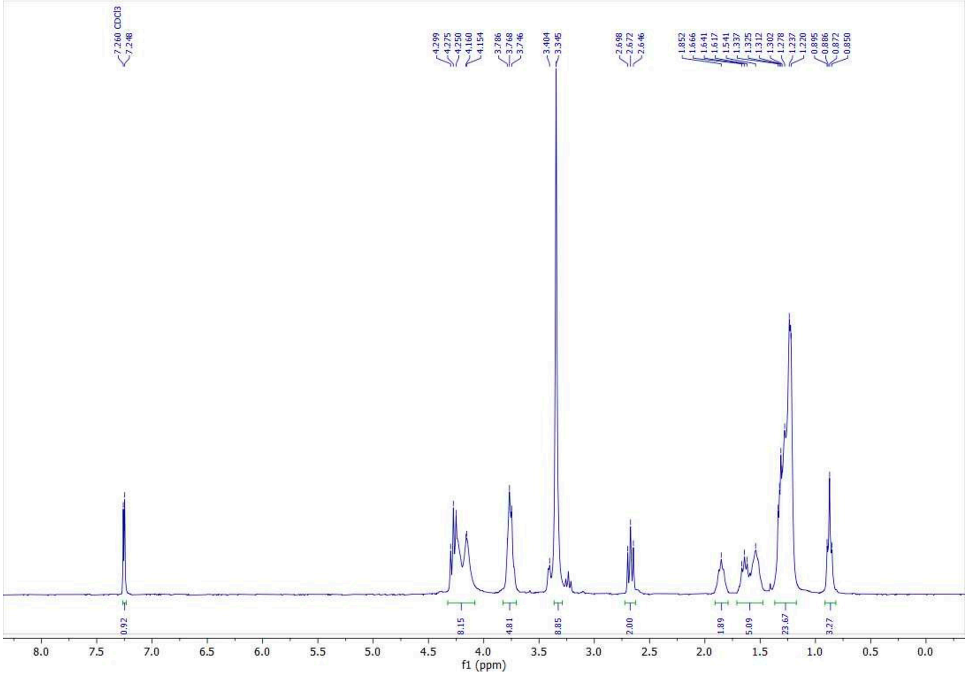

Figure S1. ^{1}H-NMR of compound **25** in $CDCl_3$ at 600 MHz.

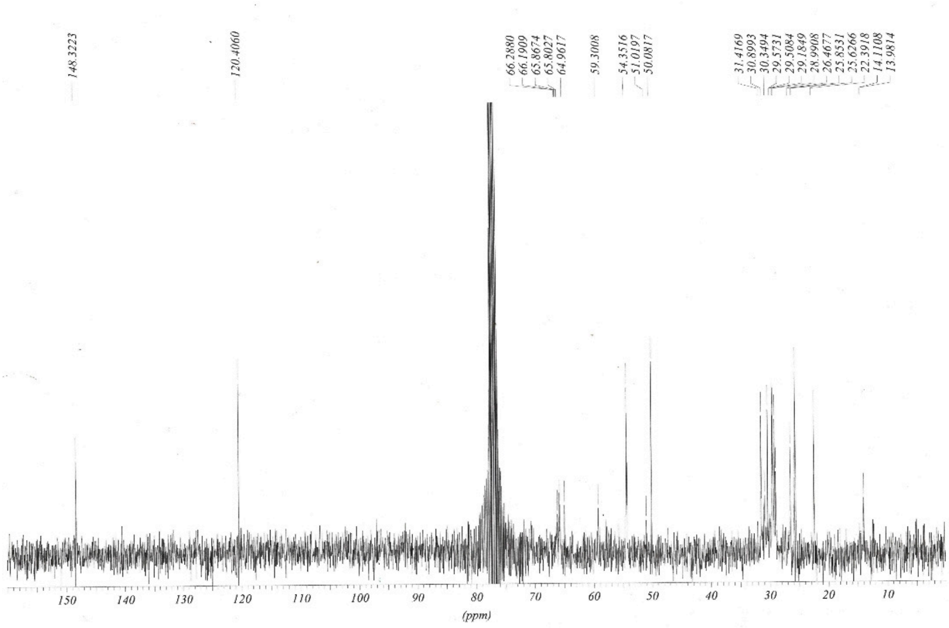

Figure S2. ^{13}C-NMR of compound **25** in $CDCl_3$ at 150 MHz.

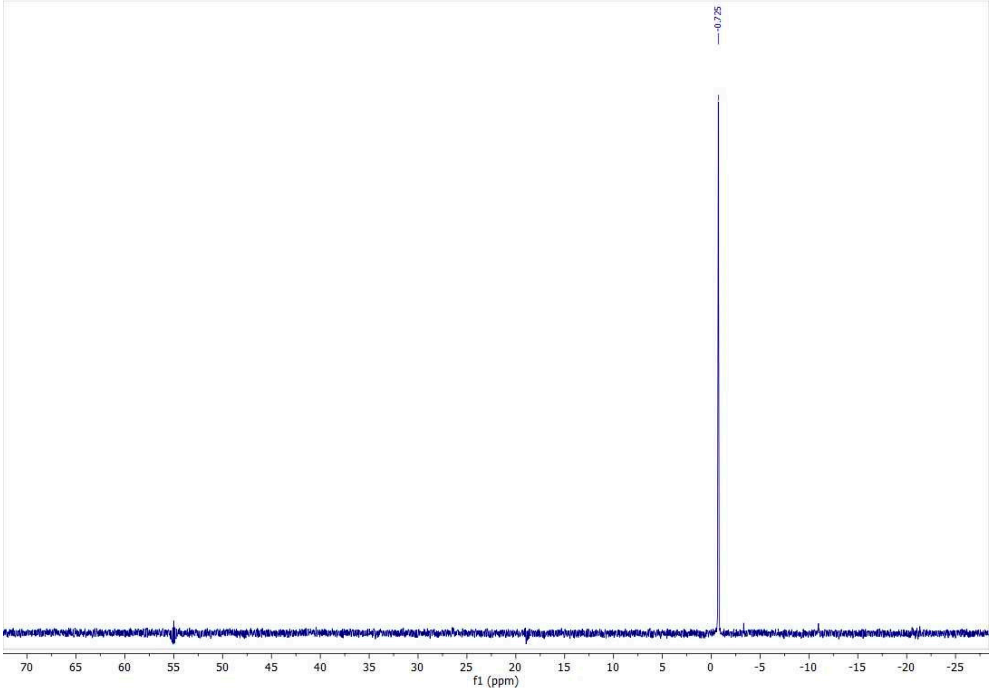

Figure S3. ^{31}P-NMR of compound **25** in $CDCl_3$ at 121.44 MHz.

Compound 26

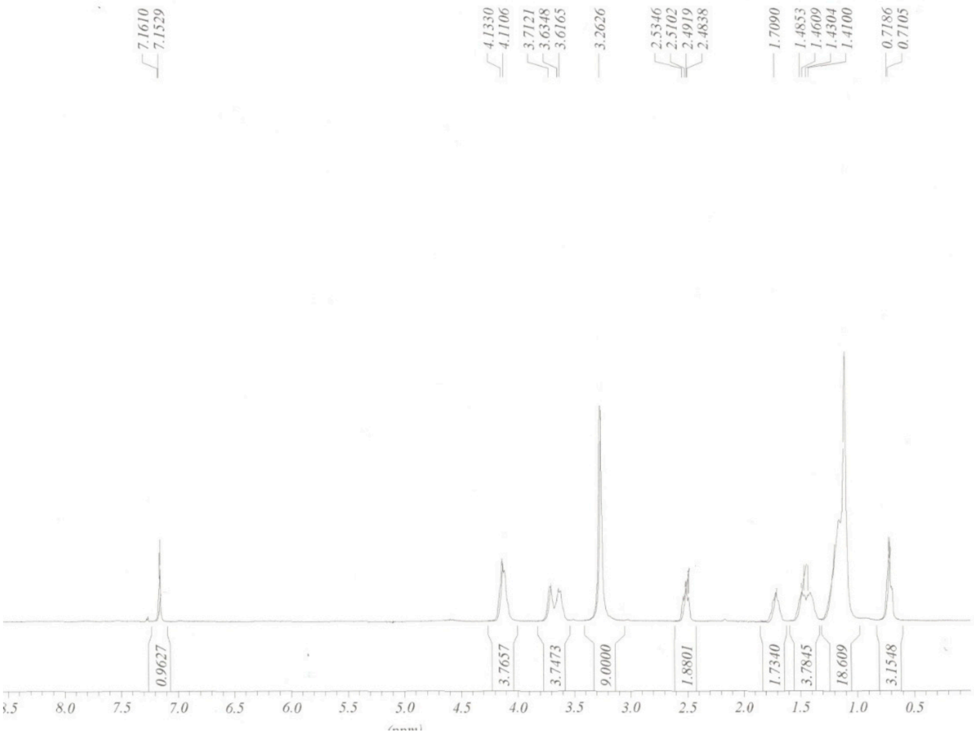

Figure S4. ^{1}H-NMR of compound **26** in $CDCl_3$ at 300 MHz.

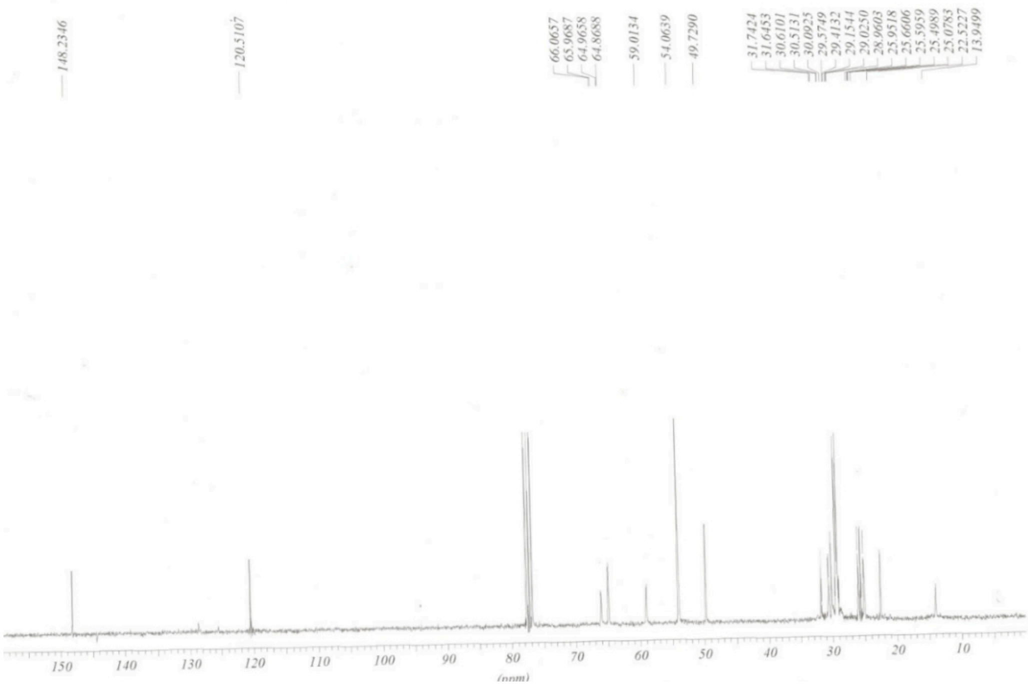

Figure S5. ^{13}C-NMR of compound **26** in $CDCl_3$ at 75 MHz.

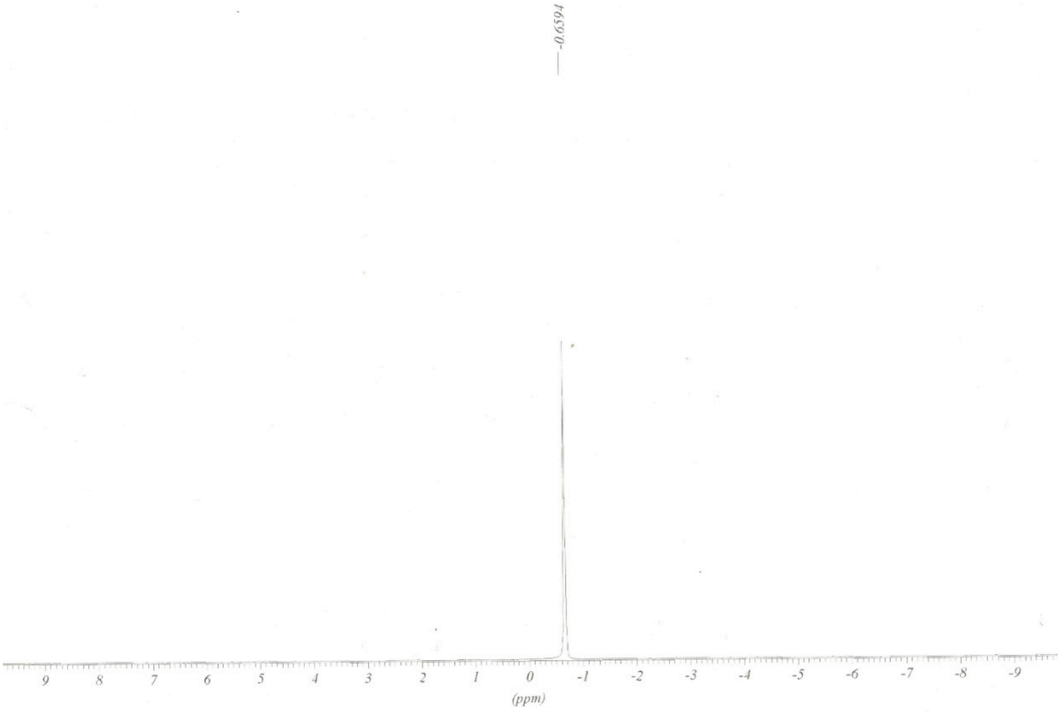

Figure S6. ^{31}P-NMR of compound **25** in $CDCl_3$ at 121.44 MHz.

Compound 27

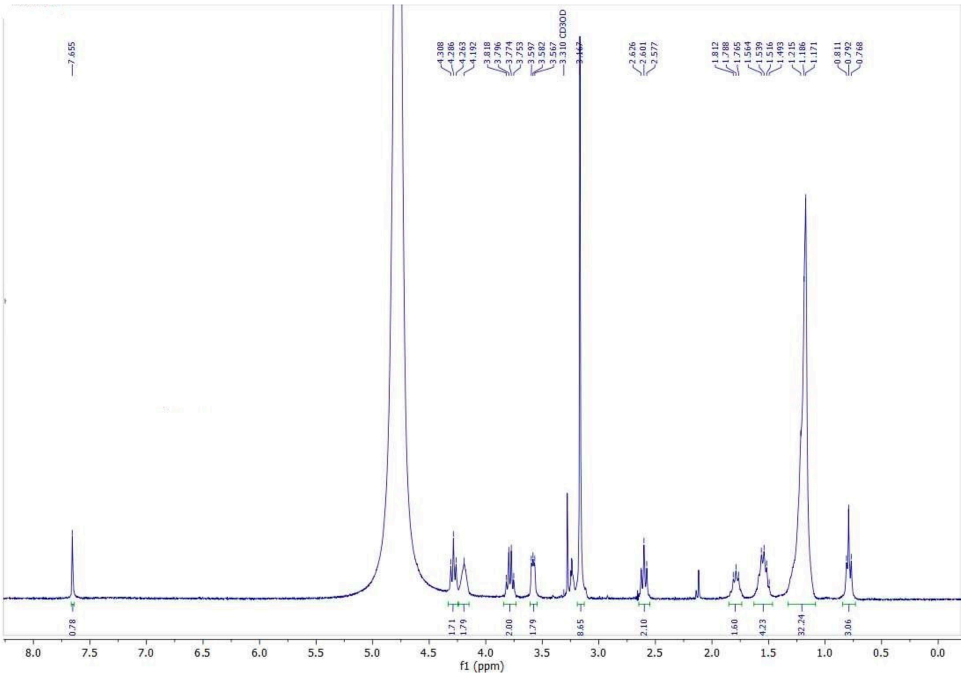

Figure S7. ^{1}H-NMR of compound **27** in CD_3OD at 600 MHz.

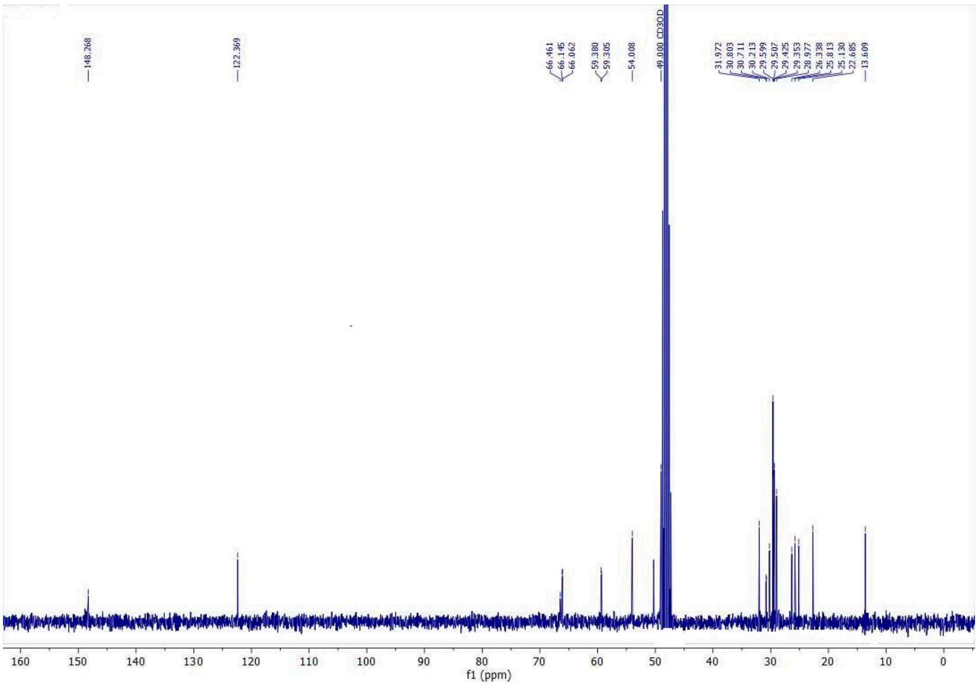

Figure S8. ^{13}C-NMR of compound **27** in CD_3OD at 150 MHz.

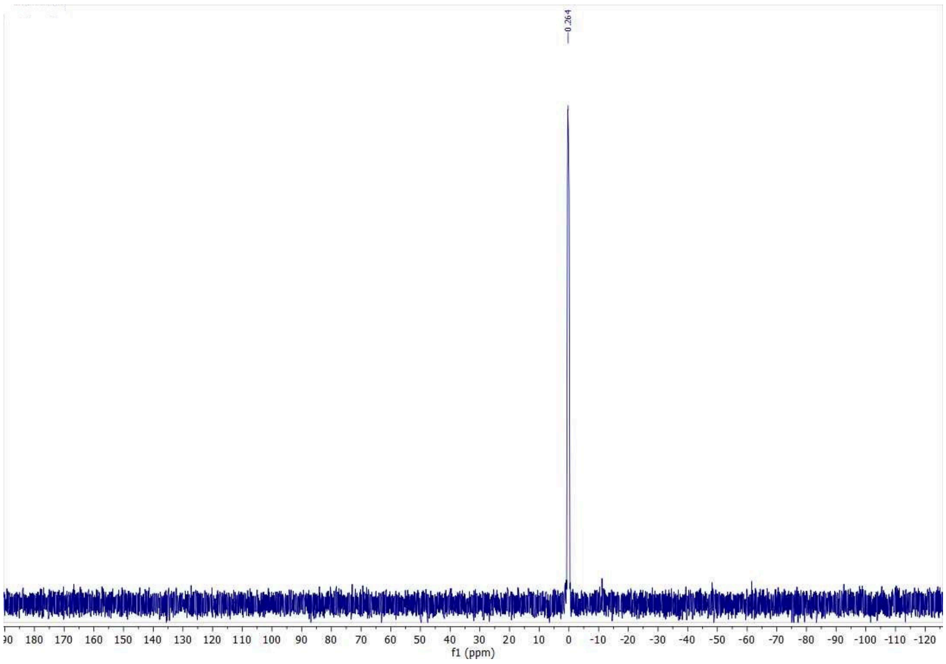

Figure S9. ^{31}P-NMR of compound **27** in CD_3OD at 121.44 MHz.

Compound 28

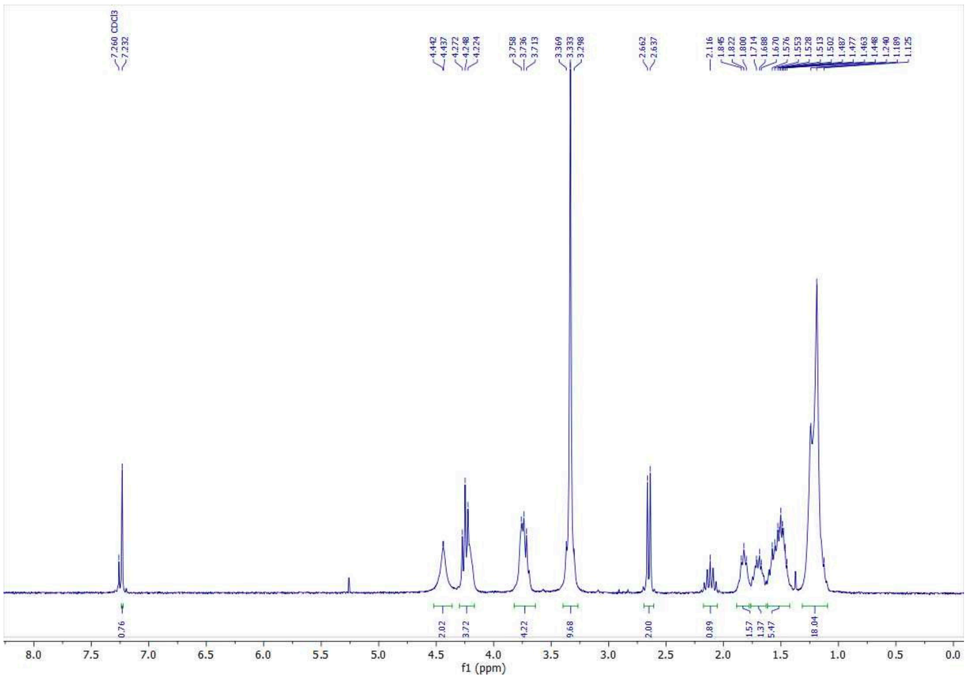

Figure S10. ^{1}H-NMR of compound **28** in $CDCl_3$ at 600 MHz.

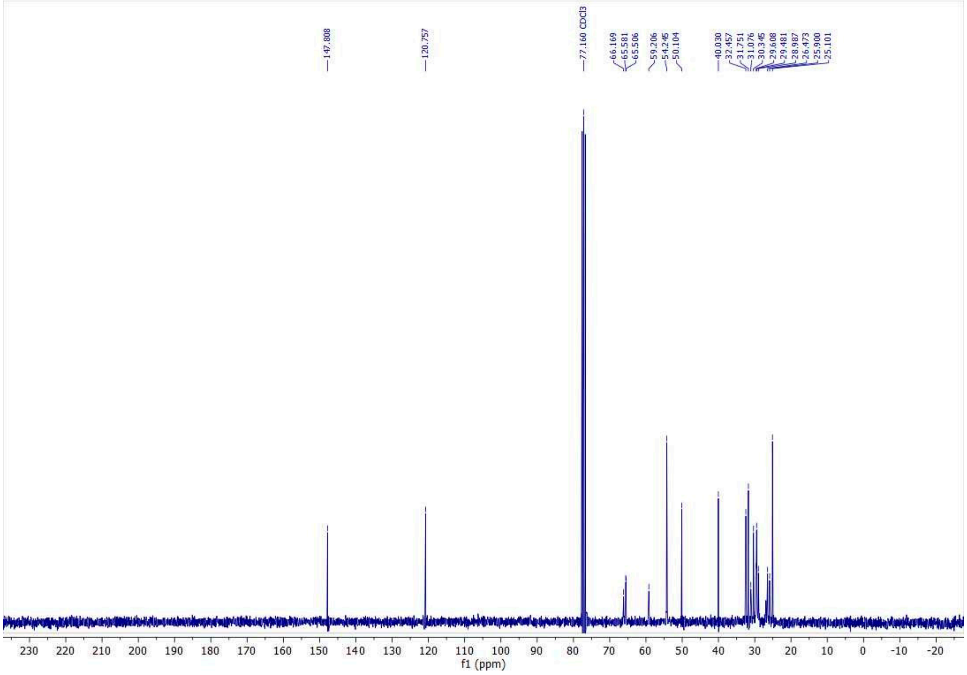

Figure S11. ^{13}C-NMR of compound **28** in $CDCl_3$ at 150 MHz.

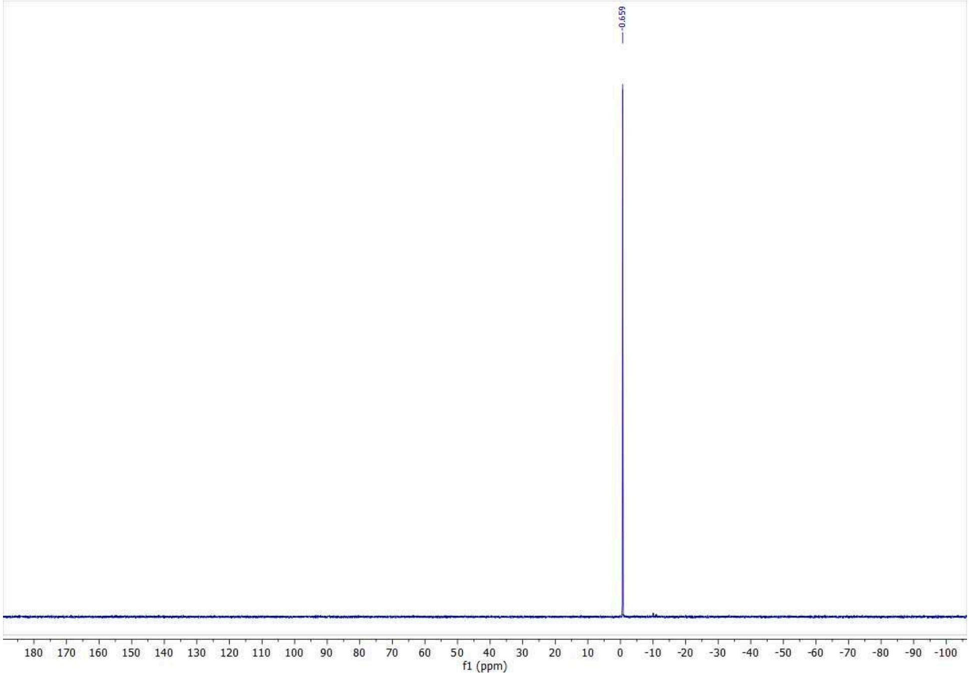

Figure S12. ^{31}P-NMR of compound **28** in $CDCl_3$ at 121.44 MHz.

Compound 29

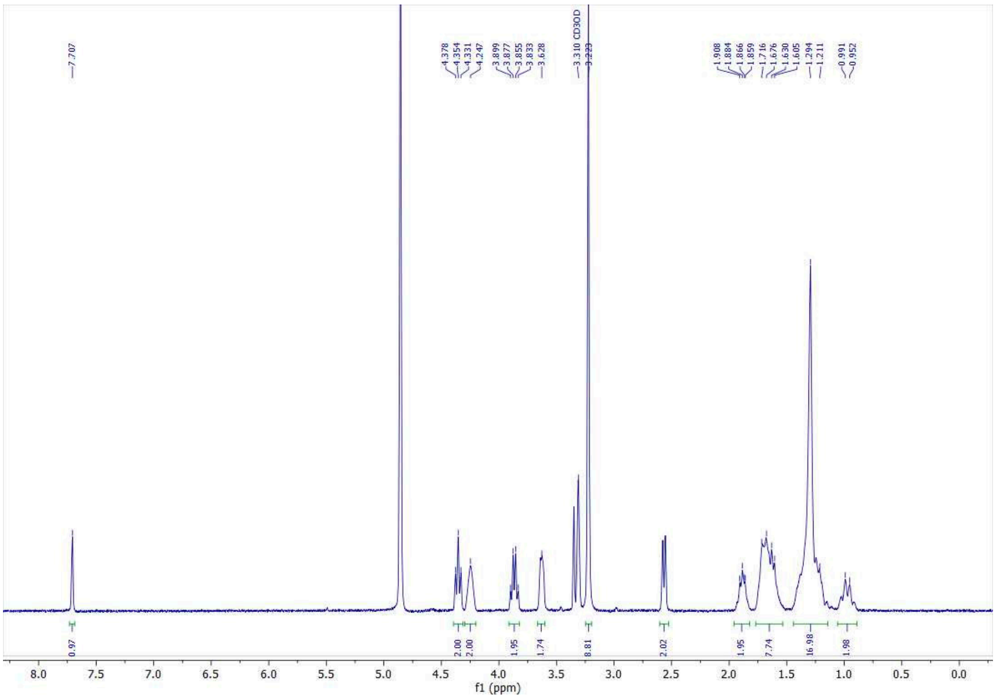

Figure S13. ^{1}H-NMR of compound **29** in CD_3OD at 600 MHz.

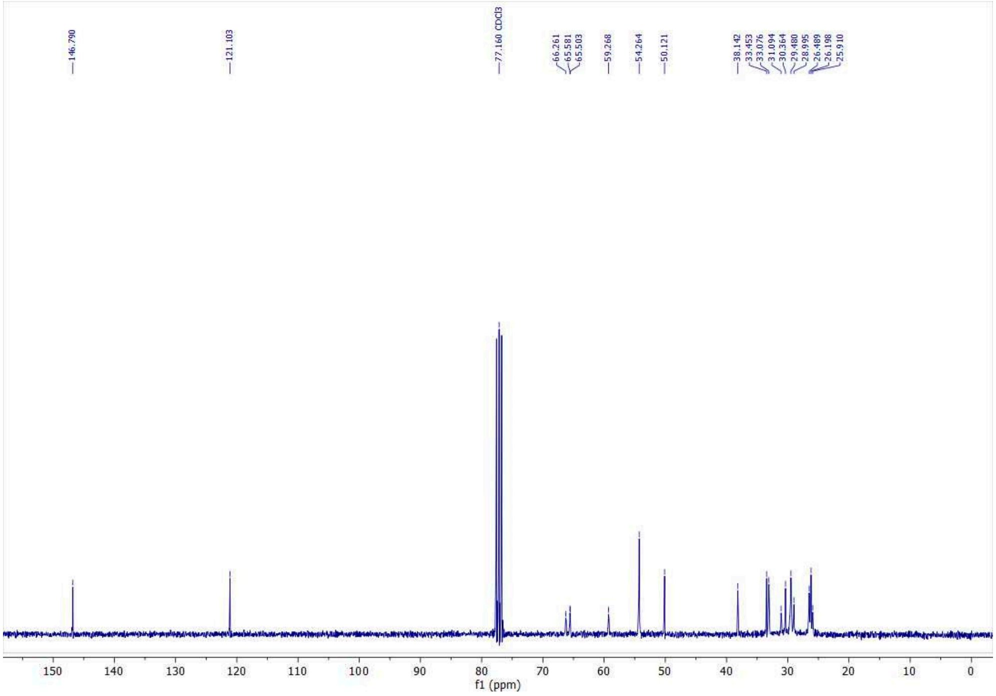

Figure S14. ^{13}C-NMR of compound **29** in CD_3OD at 150 MHz.

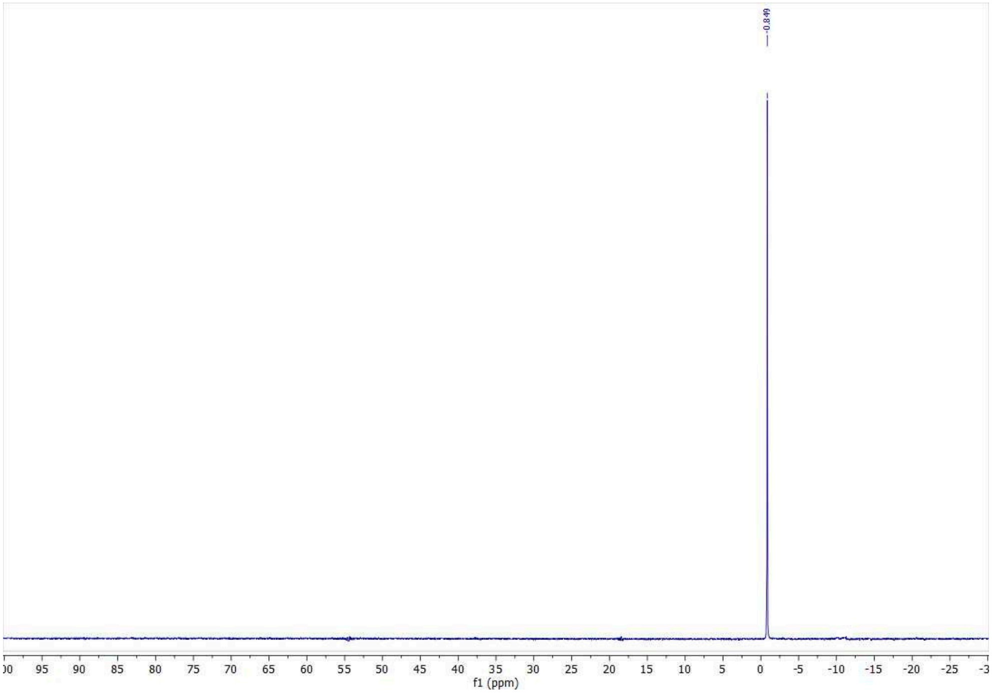

Figure S15. ^{31}P-NMR of compound **29** in CD_3OD at 121.44 MHz.

Compound 30

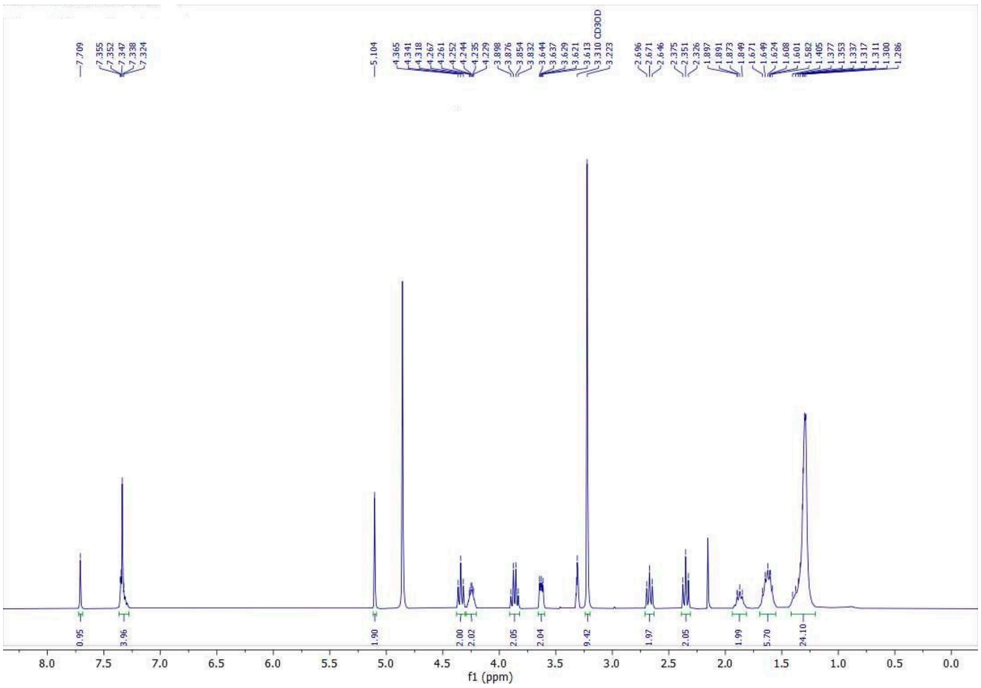

Figure S16. ^{1}H-NMR of compound **30** in CD_3OD at 600 MHz.

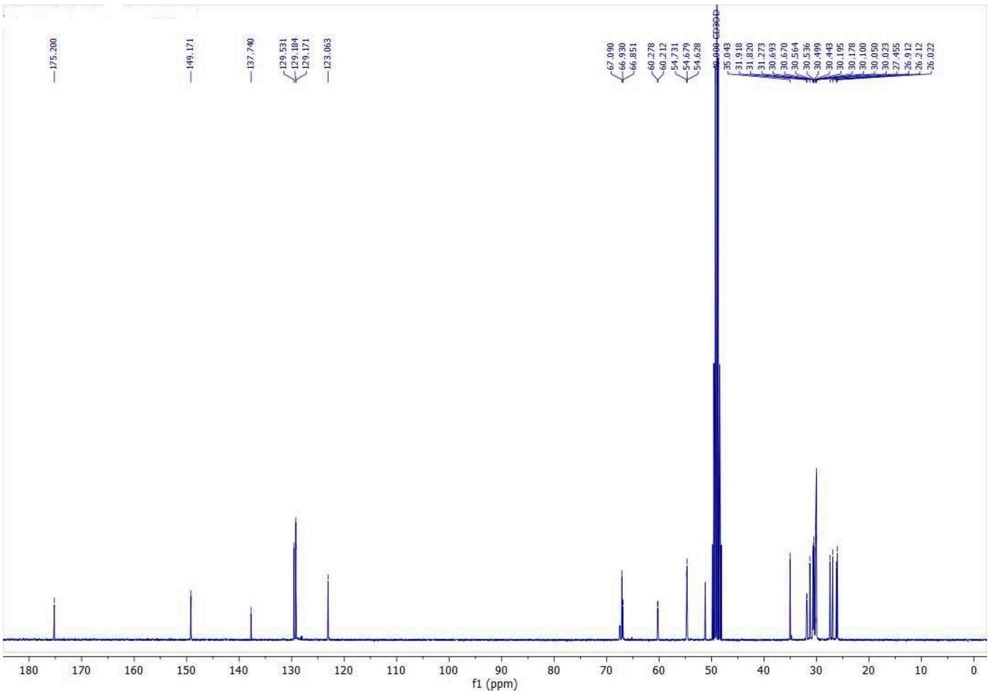

Figure S17. ^{13}C-NMR of compound **30** in CD_3OD at 150 MHz.

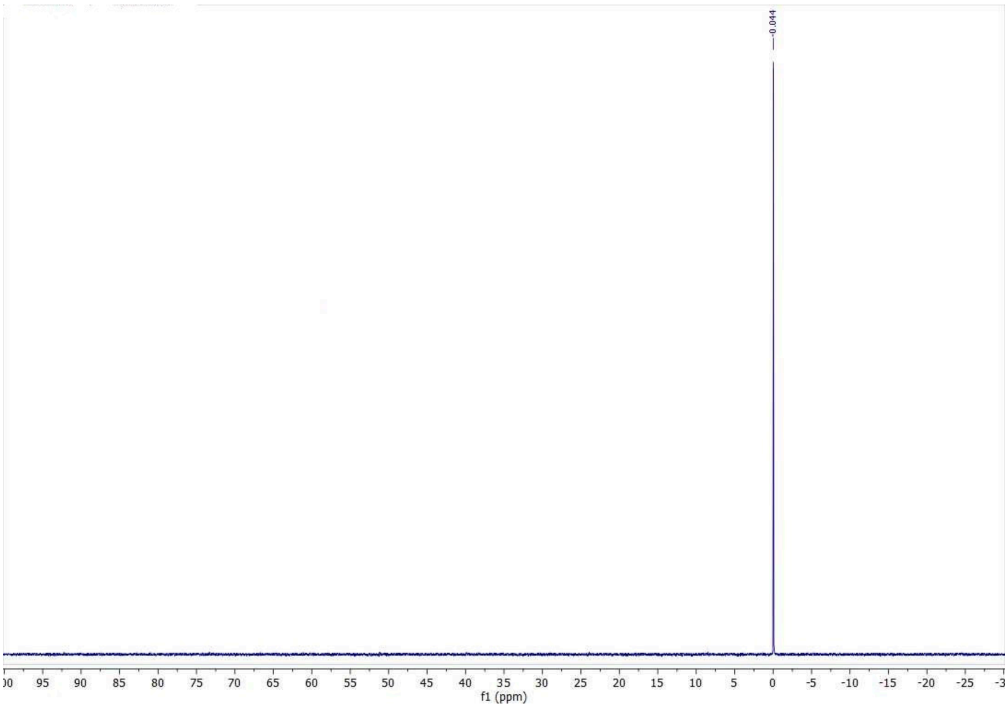

Figure S18. ^{31}P-NMR of compound **30** in CD_3OD at 121.44 MHz.

Compound 31

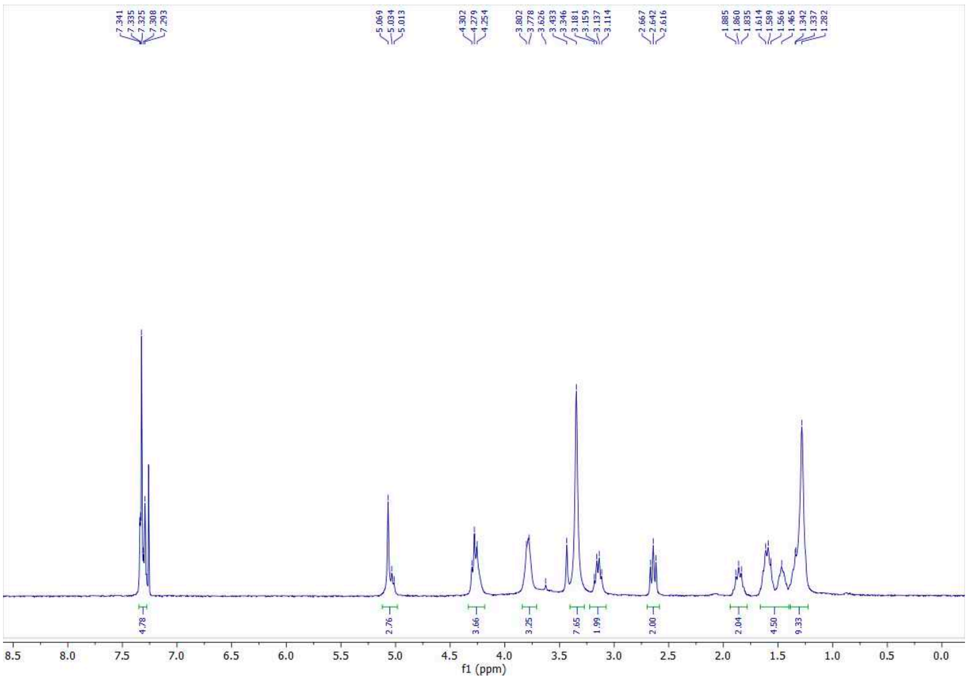

Figure S19. ^{1}H-NMR of compound **31** in $CDCl_3$ at 600 MHz.

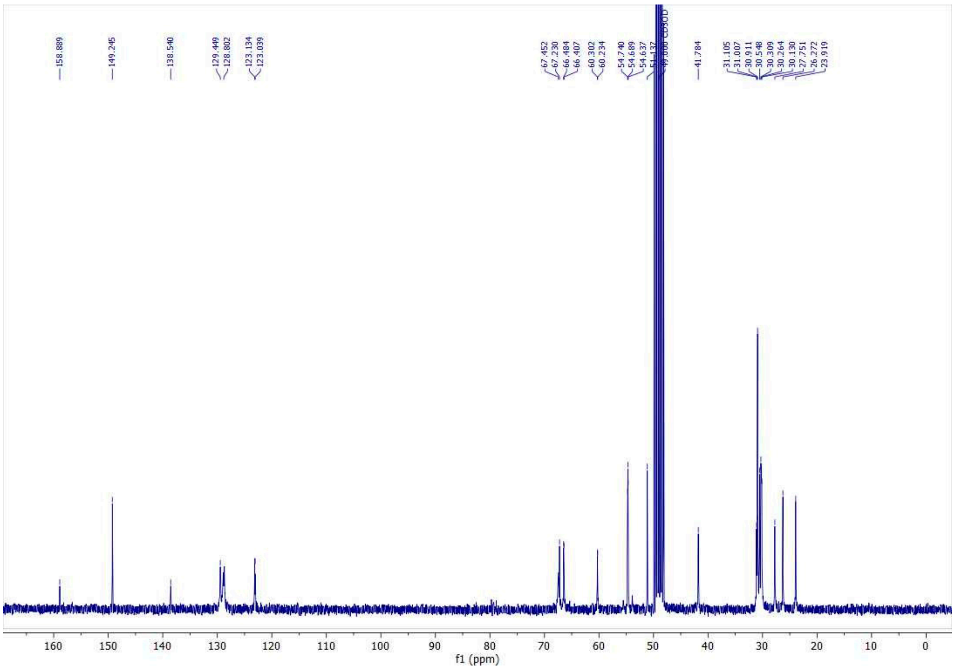

Figure S20. ^{13}C-NMR of compound **31** in CD_3OD at 150 MHz.

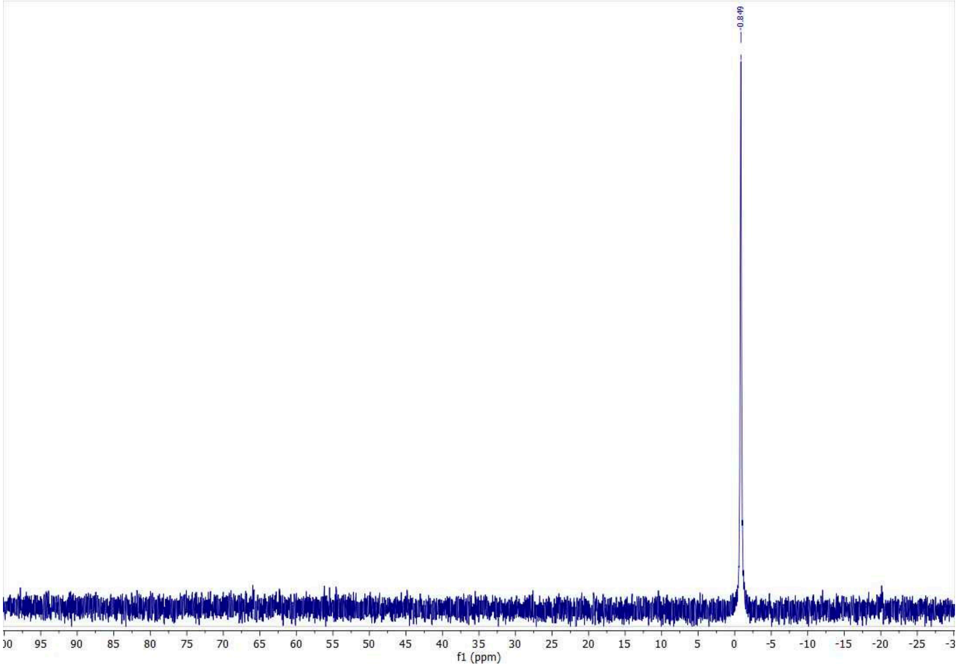

Figure S21. ^{31}P-NMR of compound **31** in CD_3OD at 121.44 MHz.

Compound 32

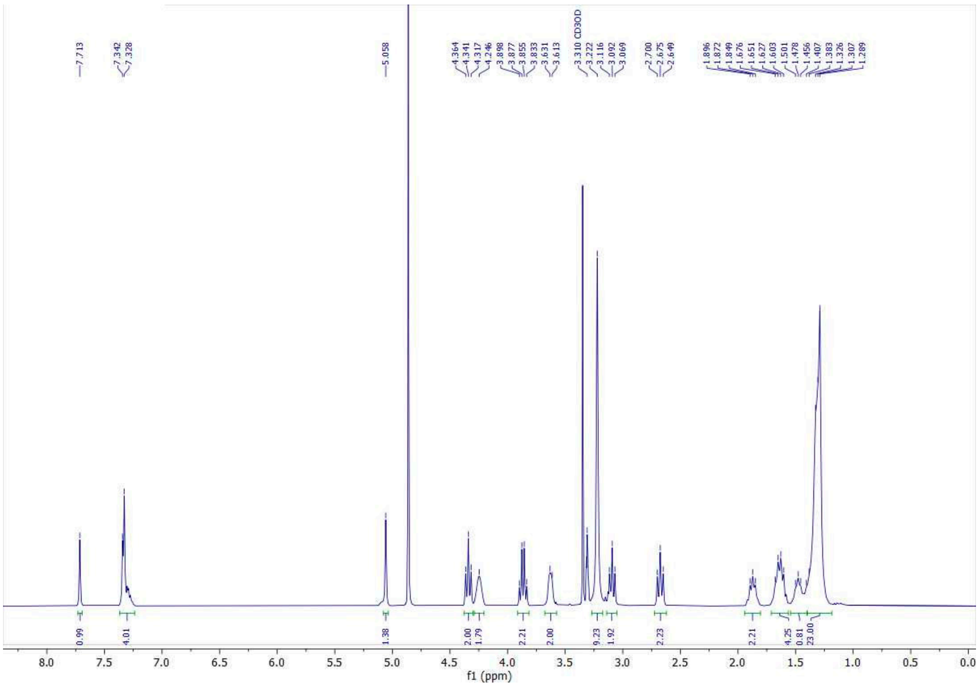

Figure S22. ^{1}H-NMR of compound **32** in CD_3OD at 600 MHz.

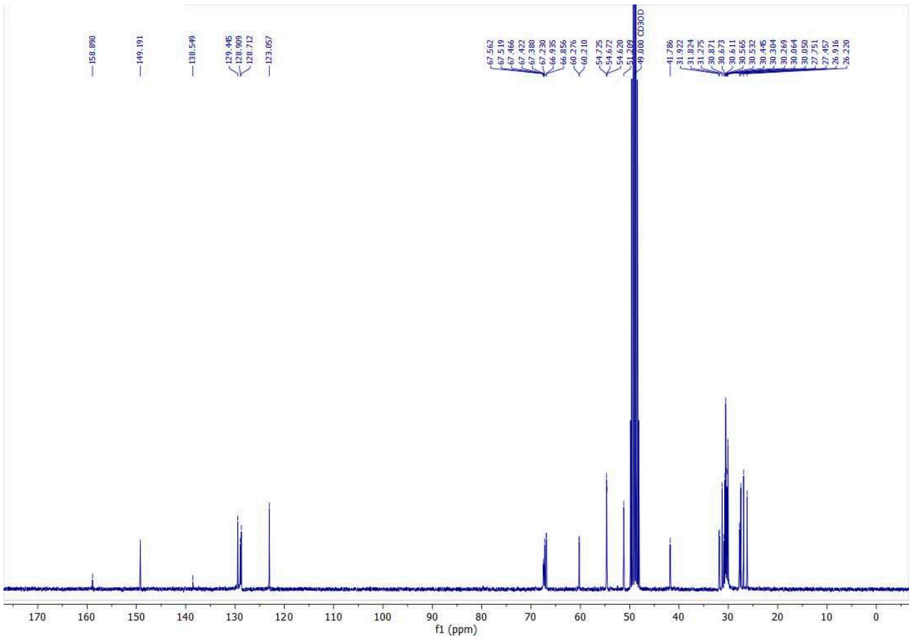

Figure S23. ^{13}C-NMR of compound **32** in CD_3OD at 150 MHz.

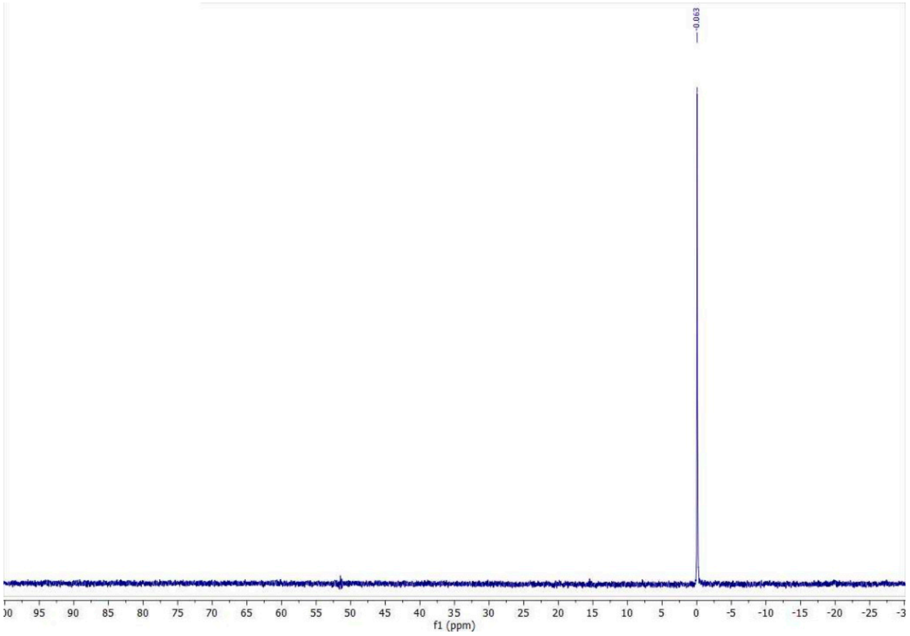

Figure S24. ^{31}P-NMR of compound **32** in CD_3OD at 121.44 MHz.

Compound 33

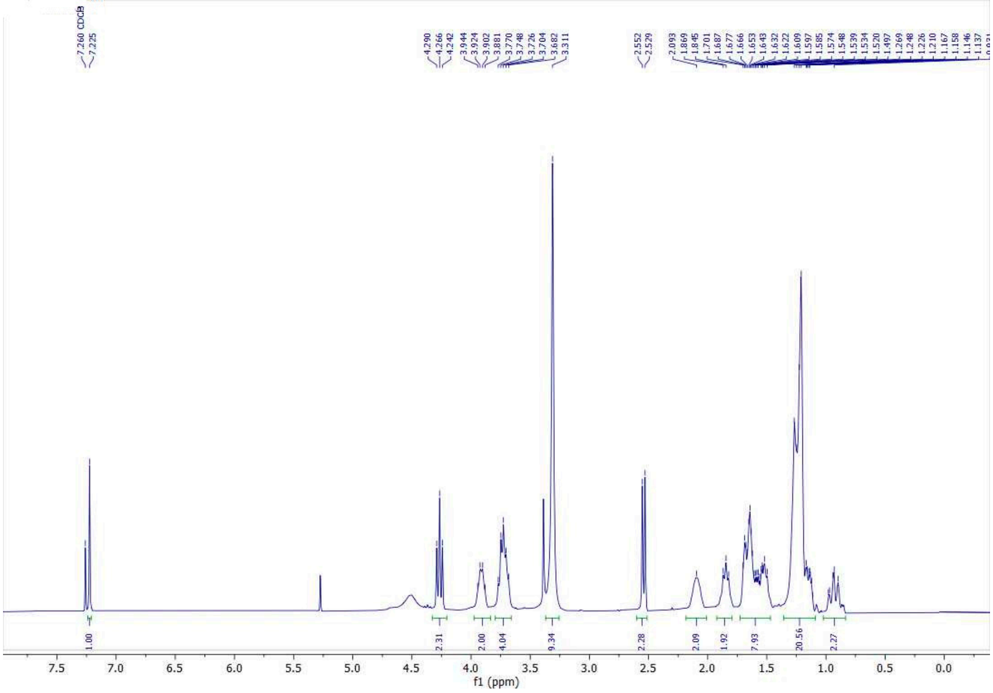

Figure S25. ^{1}H-NMR of compound **33** in $CDCl_3$ at 600 MHz.

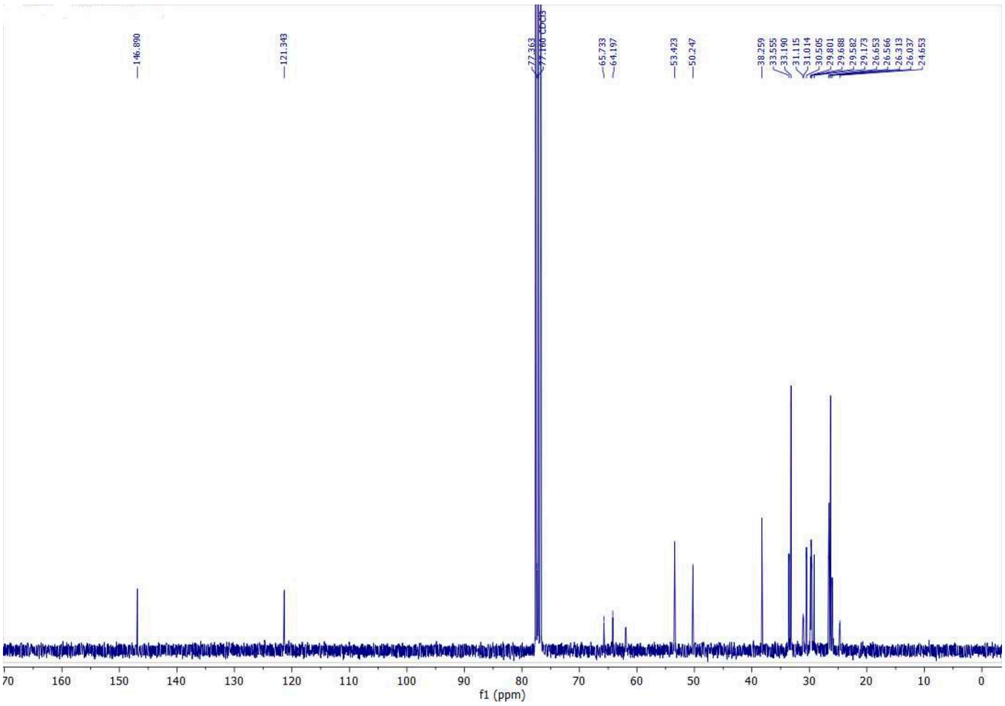

Figure S26. ^{13}C-NMR of compound **33** in $CDCl_3$ at 150 MHz.

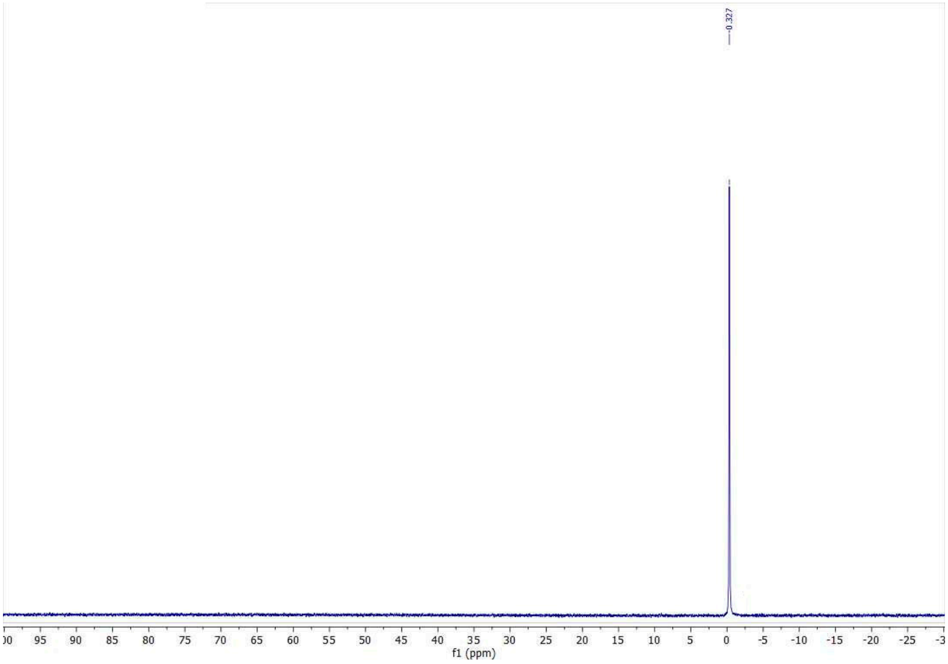

Figure S27. ^{31}P-NMR of compound **33** in $CDCl_3$ at 121.44 MHz.

Compound 34

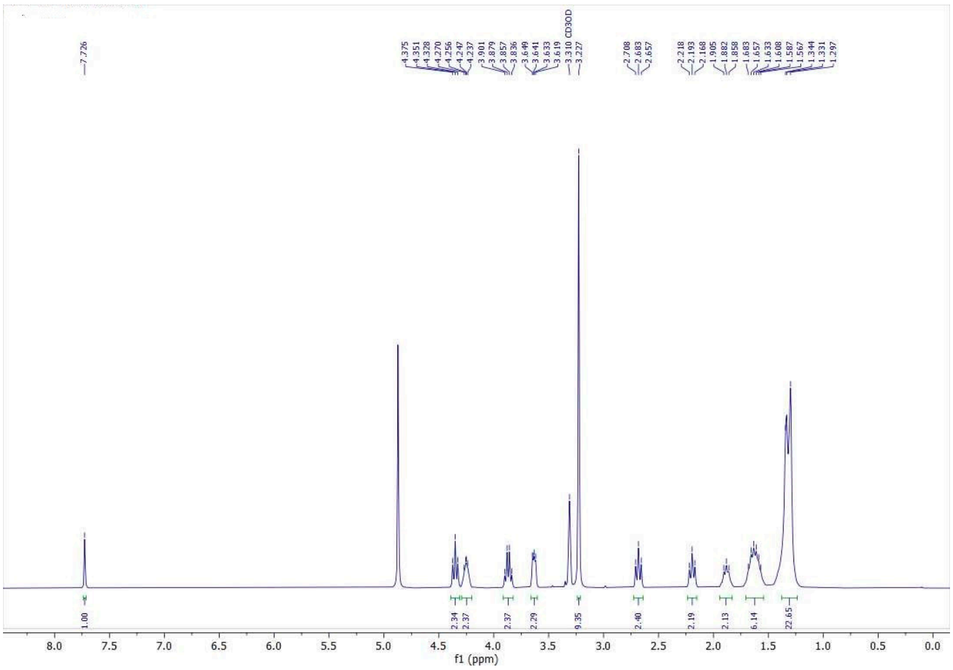

Figure S28. ^{1}H-NMR of compound **34** in CD_3OD at 600 MHz.

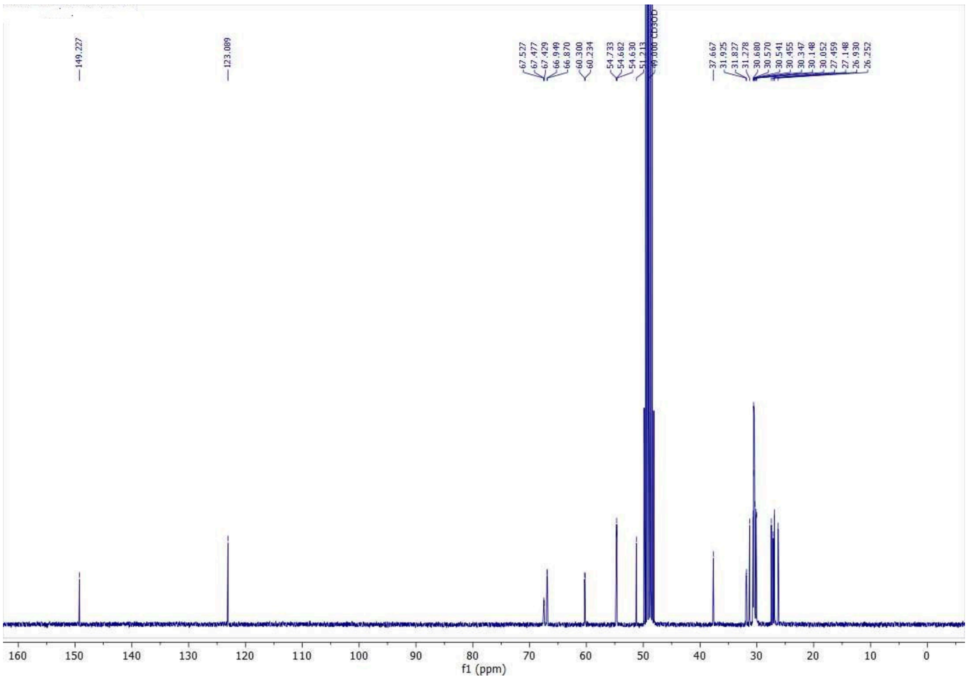

Figure S29. ^{13}C-NMR of compound **34** in CD_3OD at 150 MHz.

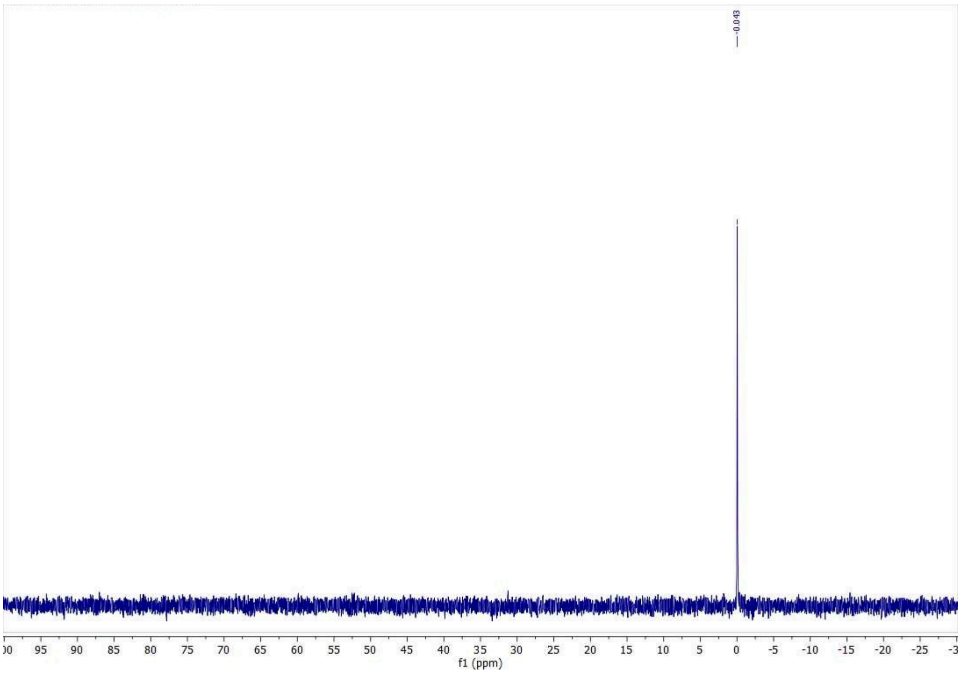

Figure S30. ^{31}P-NMR of compound **34** in CD_3OD at 121.44 MHz.

Compound 35

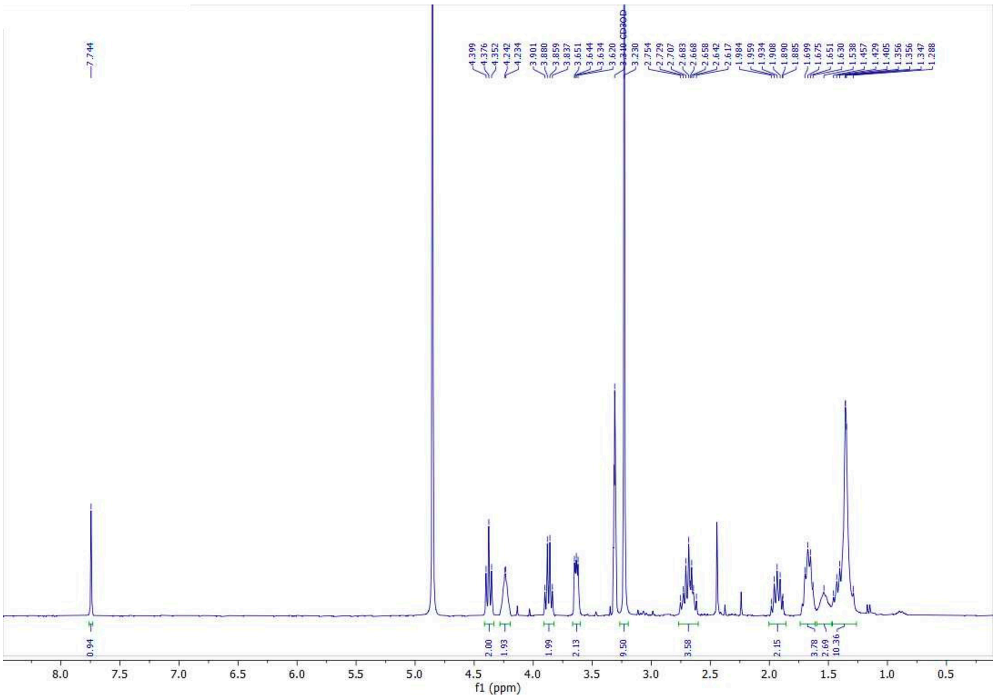

Figure S31. ^{1}H-NMR of compound **35** in CD_3OD at 600 MHz.

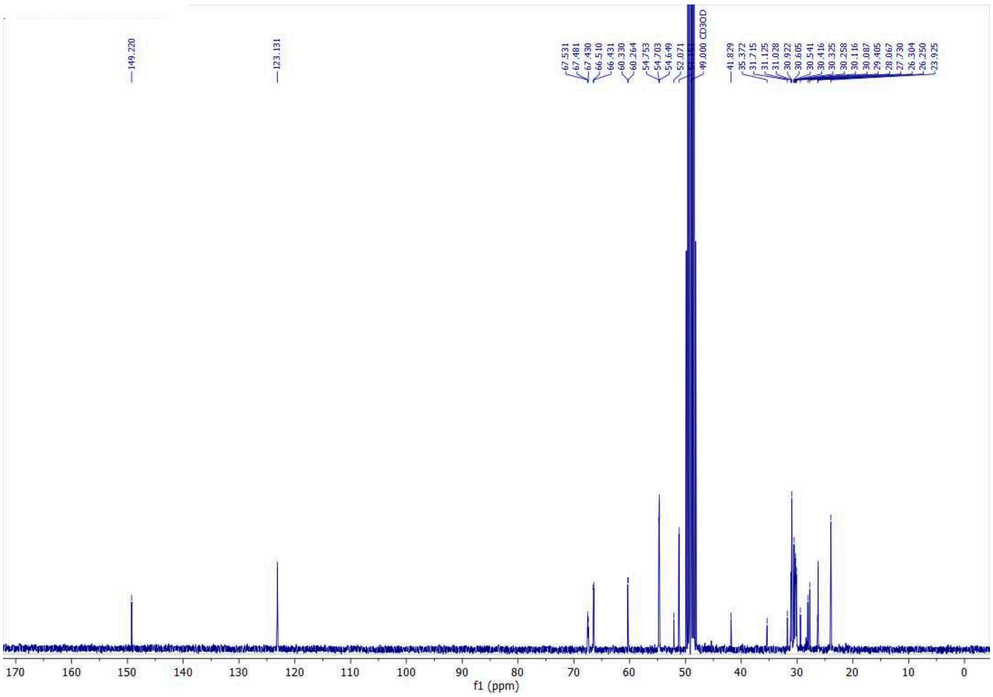

Figure S32. ^{13}C-NMR of compound **35** in CD_3OD at 150 MHz.

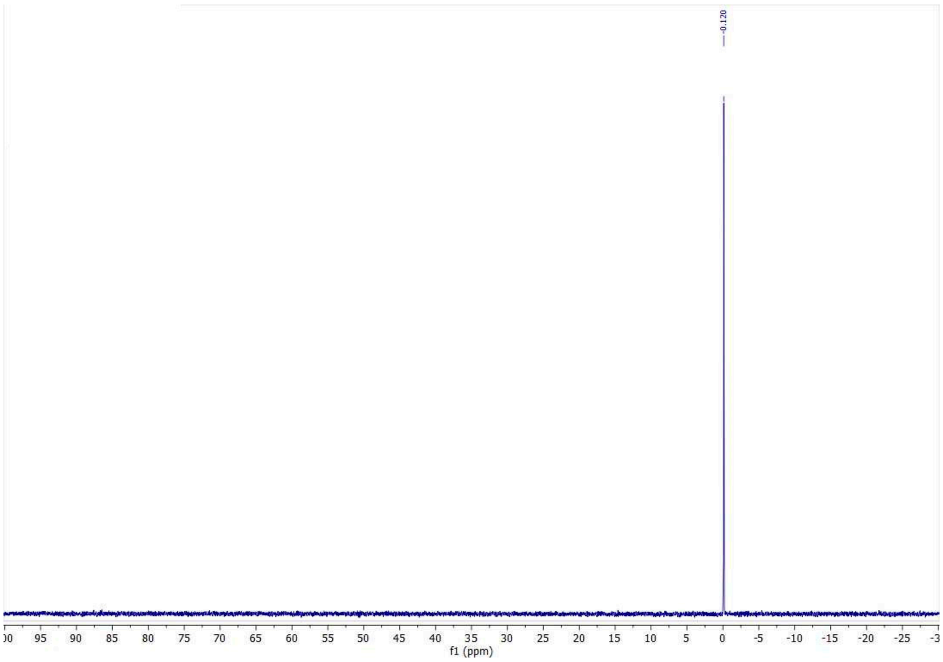

Figure S33. ^{31}P-NMR of compound **35** in CD_3OD at 121.44 MHz.

Compound 36

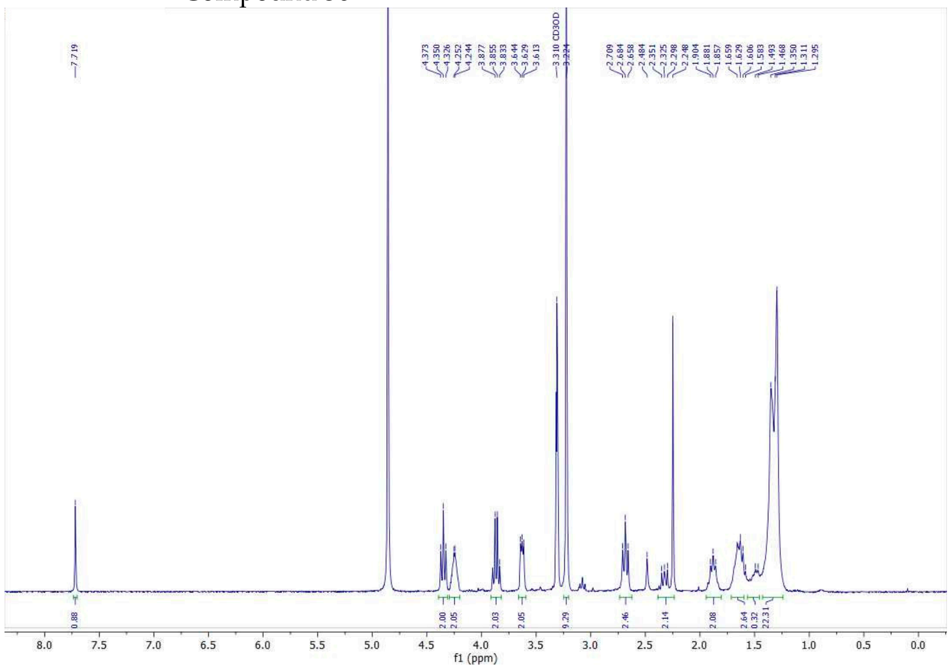

Figure S34. ^{1}H-NMR of compound **36** in CD_3OD at 600 MHz.

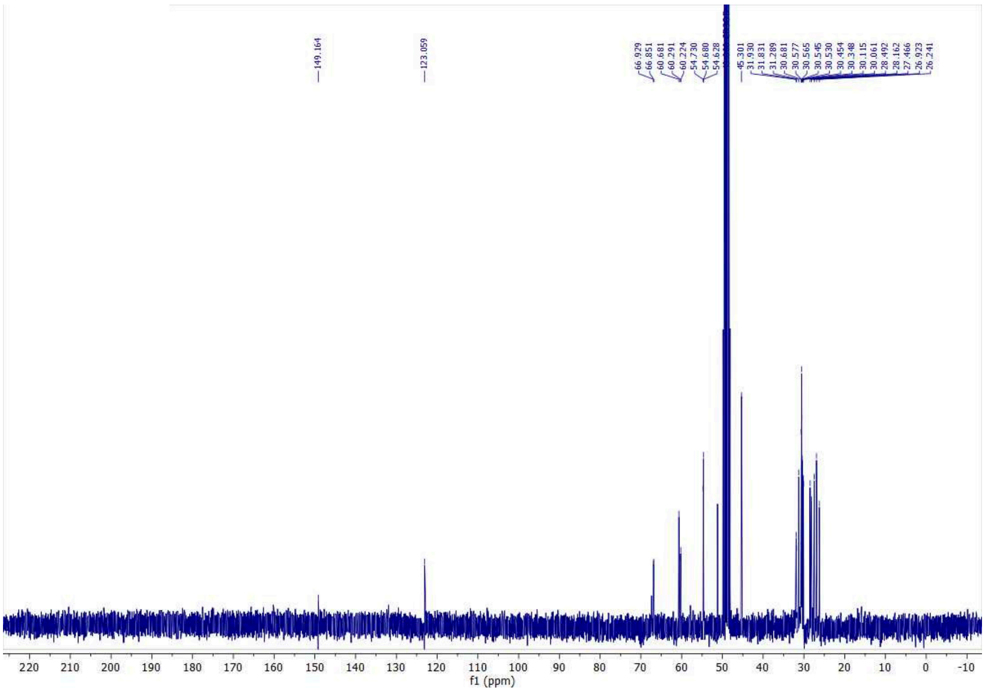

Figure S35. ^{13}C-NMR of compound **36** in CD_3OD at 150 MHz.

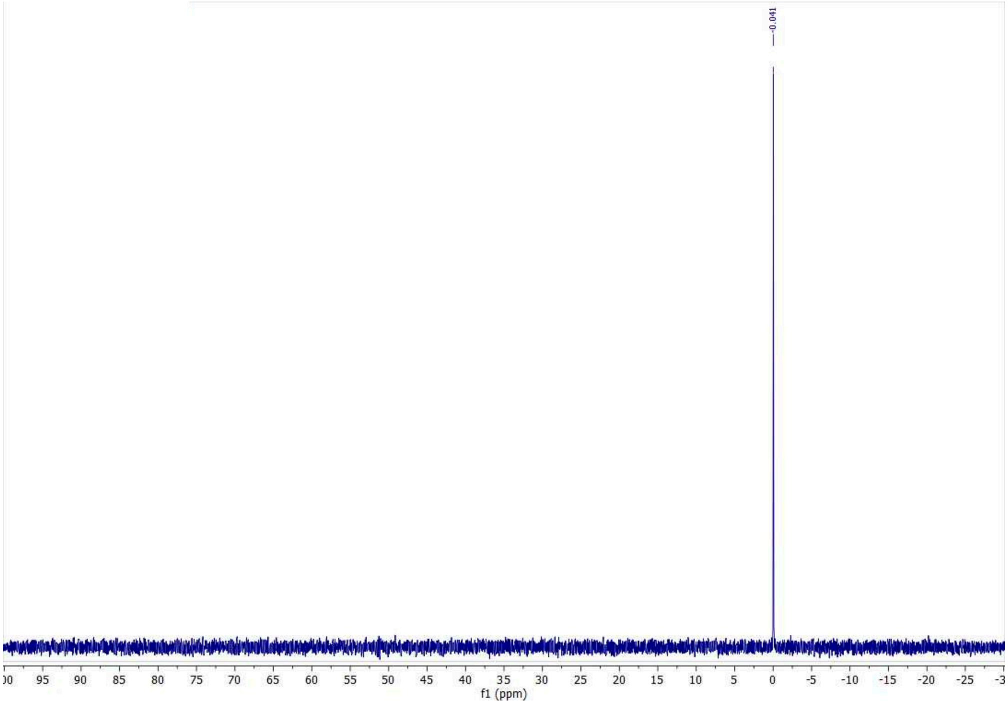

Figure S36. ^{31}P-NMR of compound **36** in CD_3OD at 121.44 MHz.

Compound 41

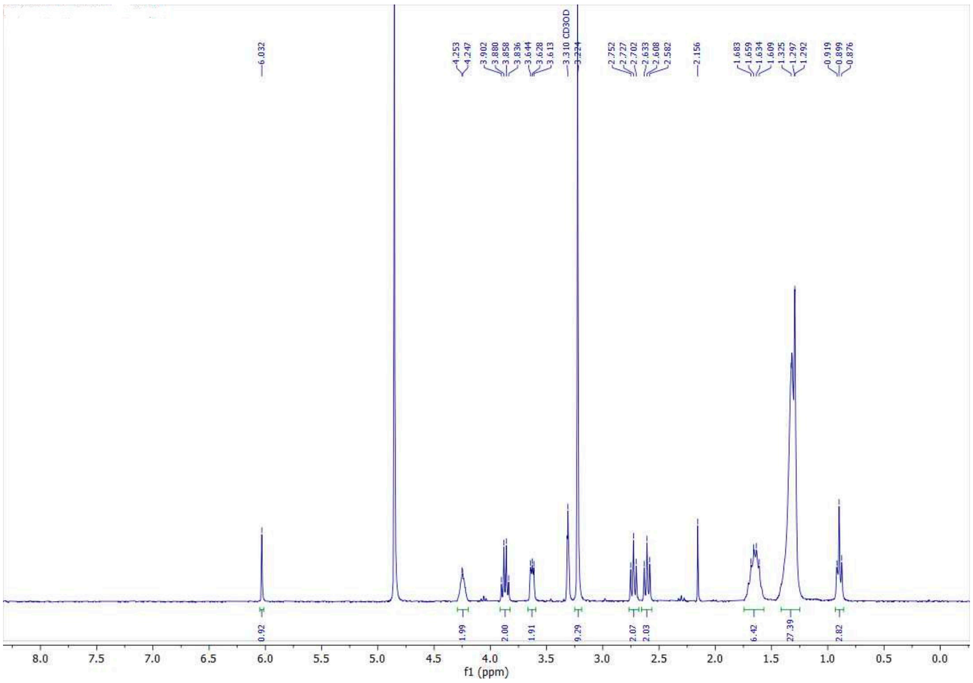

Figure S37. ^{1}H-NMR of compound **41** in CD_3OD at 600 MHz.

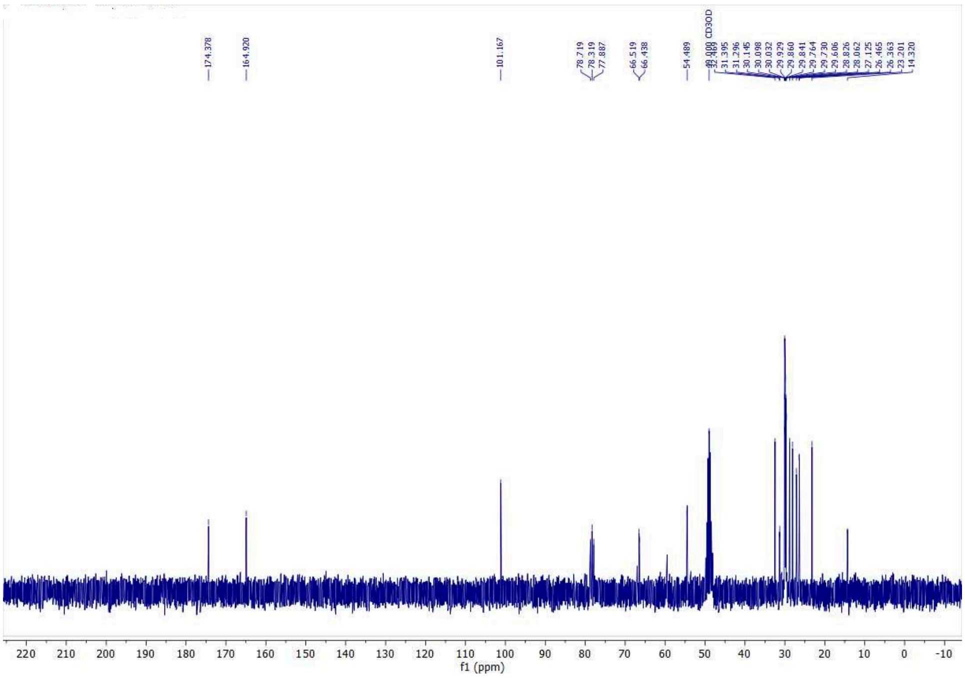

Figure S38. ^{13}C-NMR of compound **41** in CD_3OD at 150 MHz.

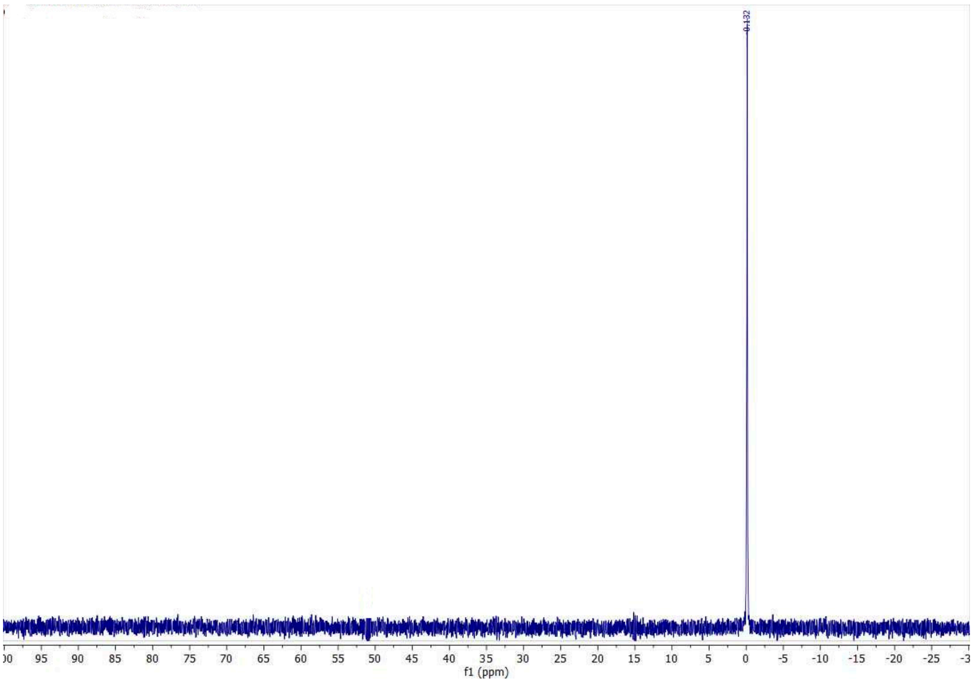

Figure S39. ^{31}P-NMR of compound **41** in CD_3OD at 121.44 MHz.

Compound 53

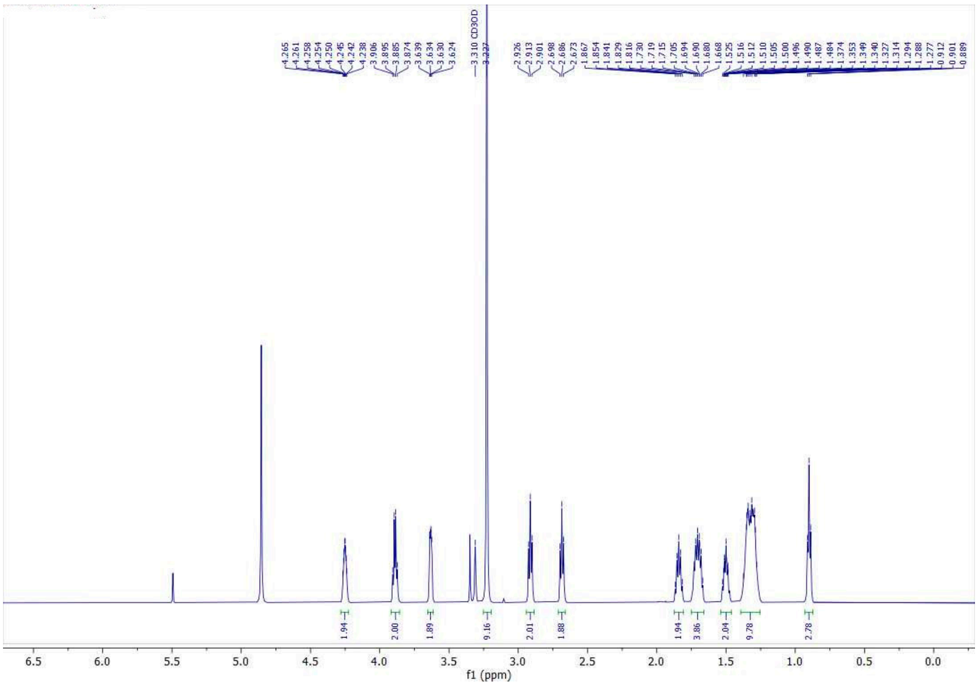

Figure S40. ^{1}H-NMR of compound **53** in CD_3OD at 600 MHz.

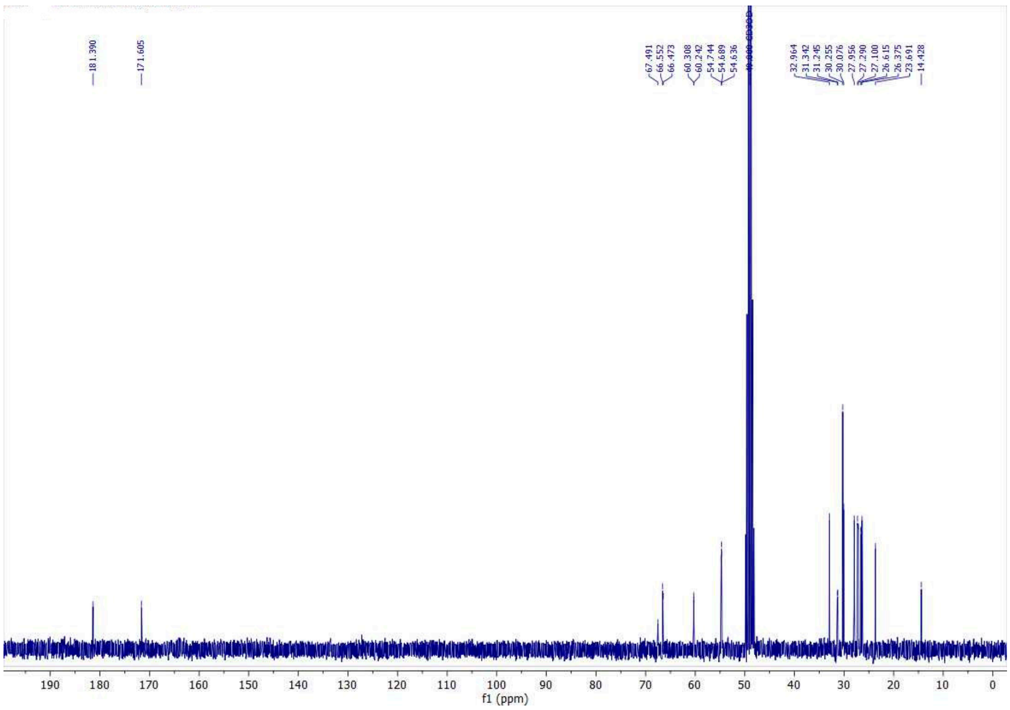

Figure S41. ^{13}C-NMR of compound **53** in CD_3OD at 150 MHz.

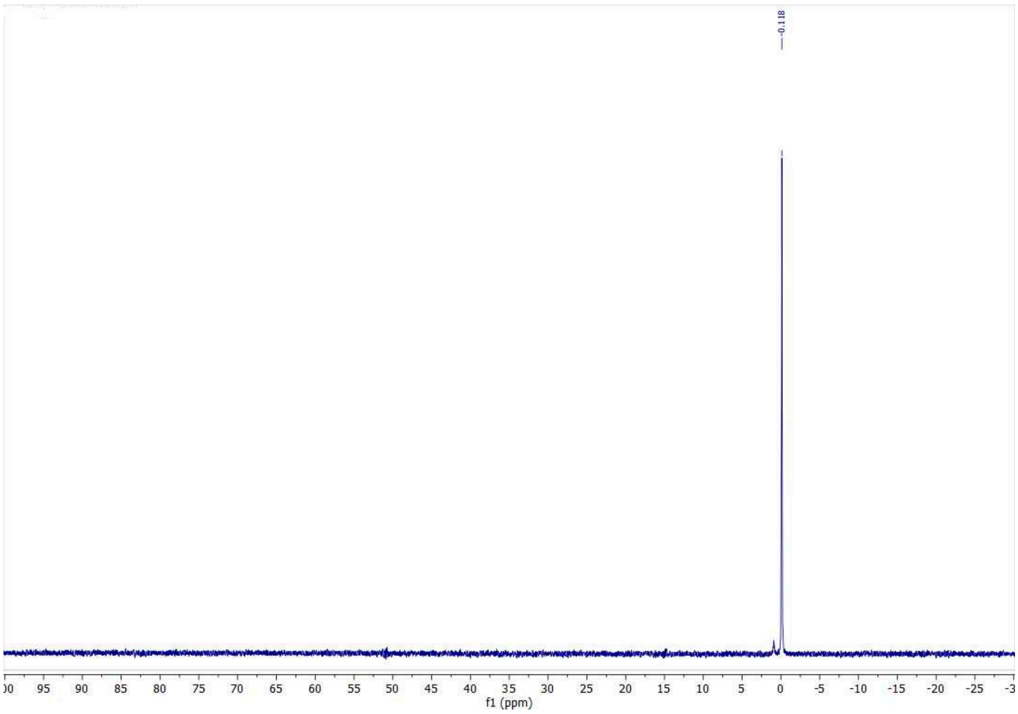

Figure S42. ^{31}P-NMR of compound **53** in CD_3OD at 121.44 MHz.

Compound 54

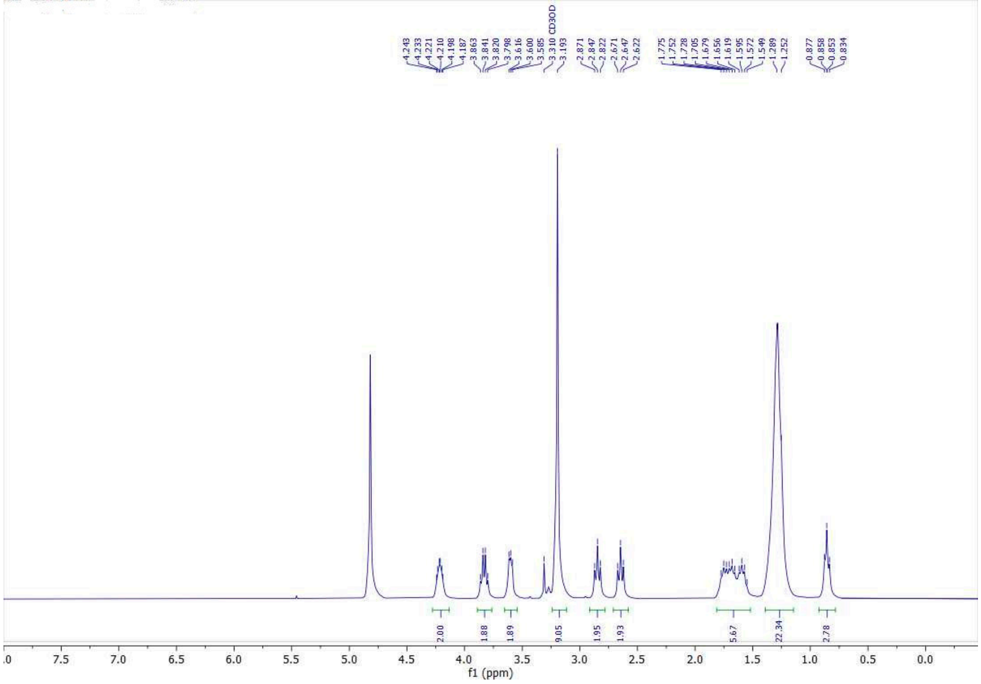

Figure S43. ^{1}H-NMR of compound **54** in CD_3OD at 600 MHz.

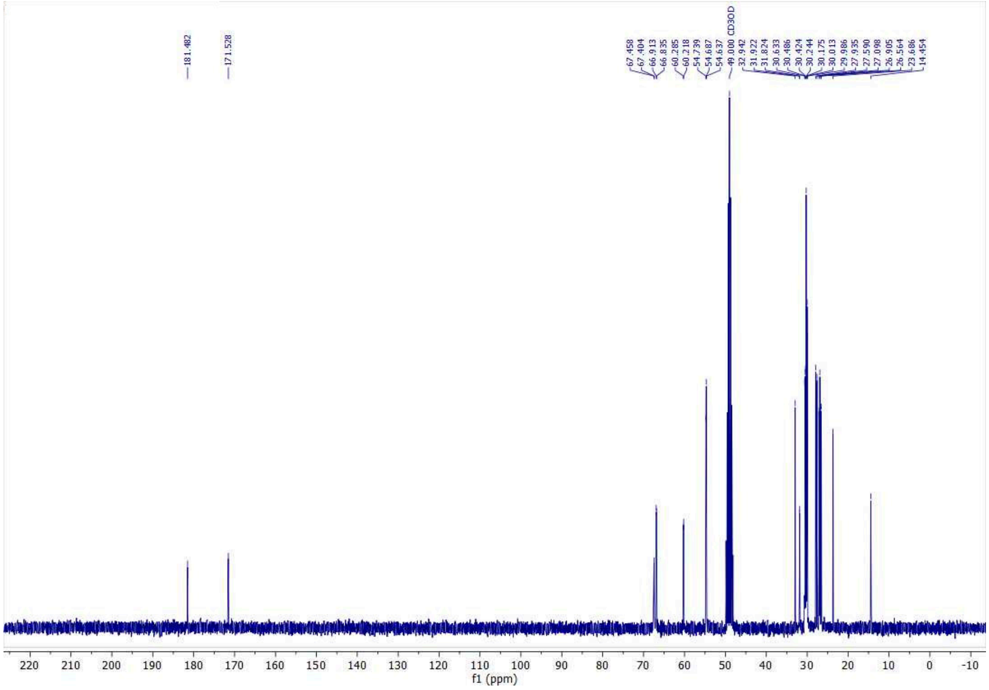

Figure S44. ^{13}C-NMR of compound **54** in CD_3OD at 150 MHz.

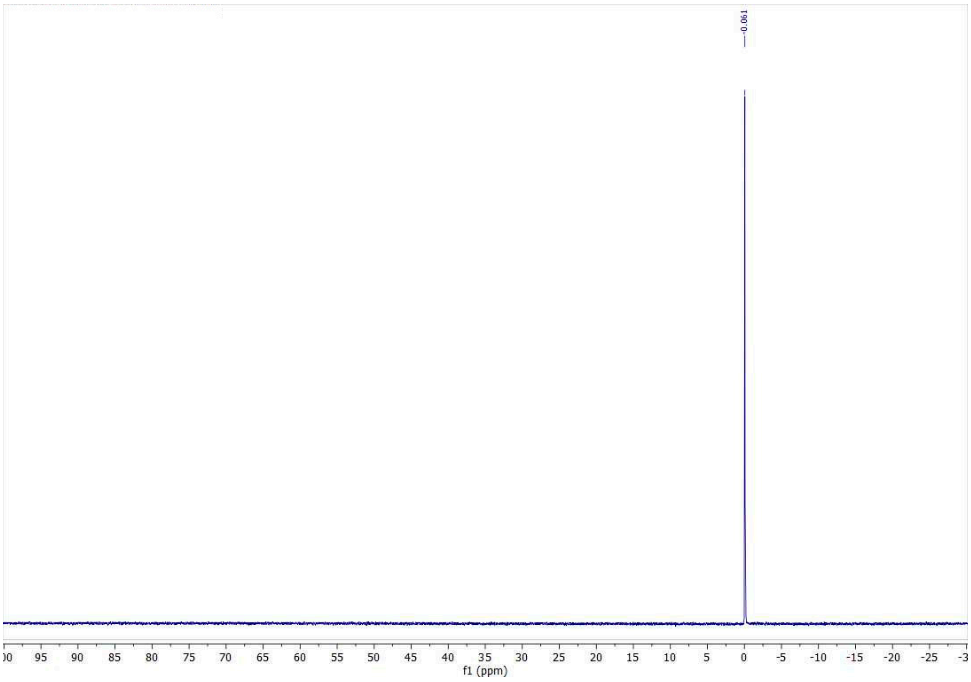

Figure S45. ^{31}P-NMR of compound **54** in CD_3OD at 121.44 MHz.

Compound 65

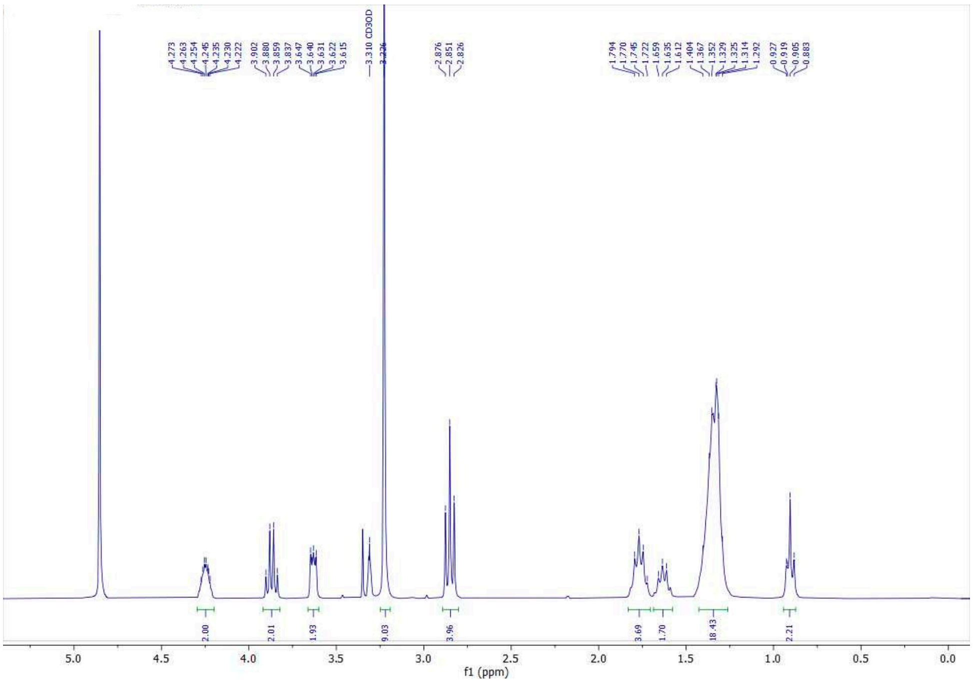

Figure S46. ^{1}H-NMR of compound **65** in CD_3OD at 600 MHz.

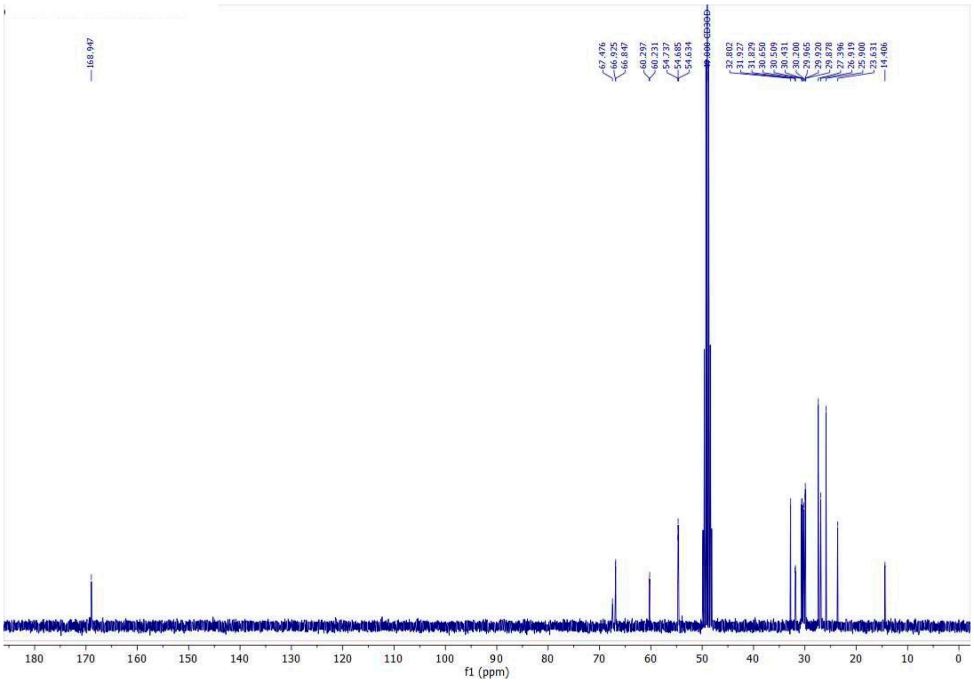

Figure S47. ^{13}C-NMR of compound **65** in CD_3OD at 150 MHz.

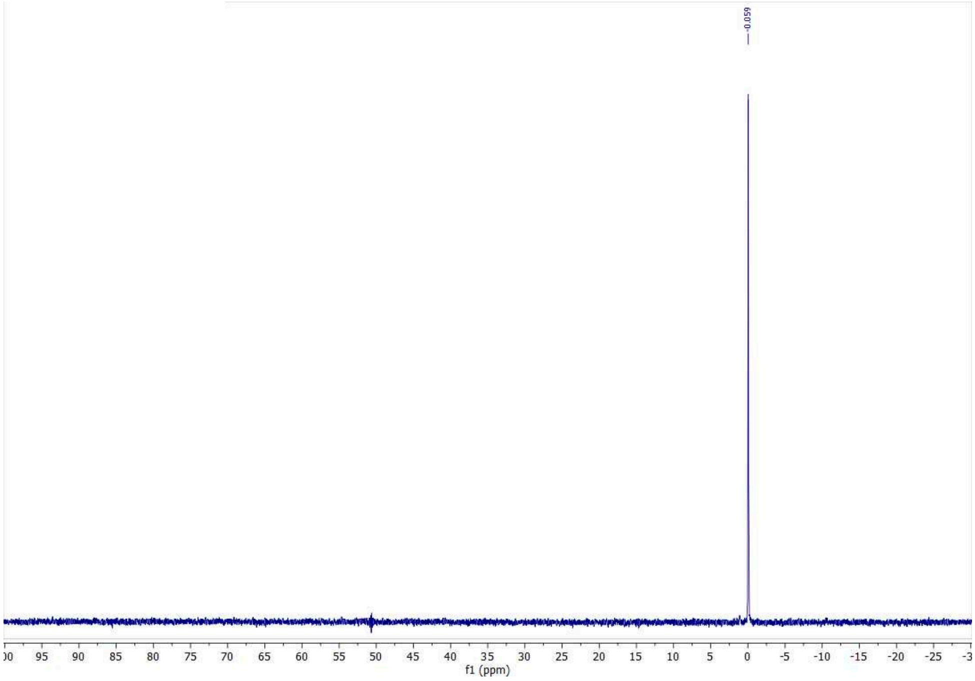

Figure S48. ^{31}P-NMR of compound **65** in CD_3OD at 121.44 MHz.

Compound 66

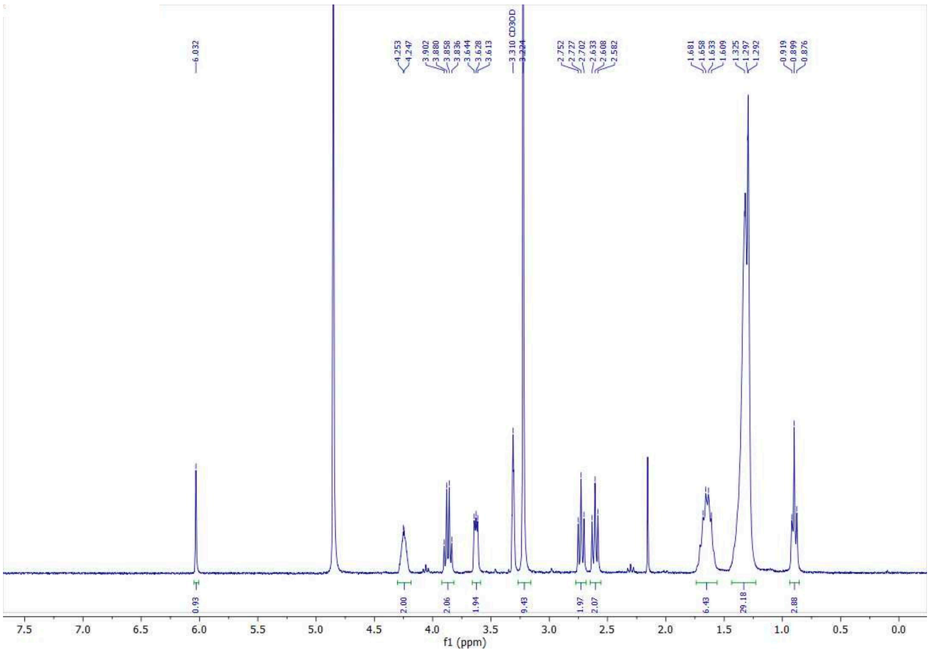

Figure S49. ^{1}H-NMR of compound **66** in CD_3OD at 600 MHz.

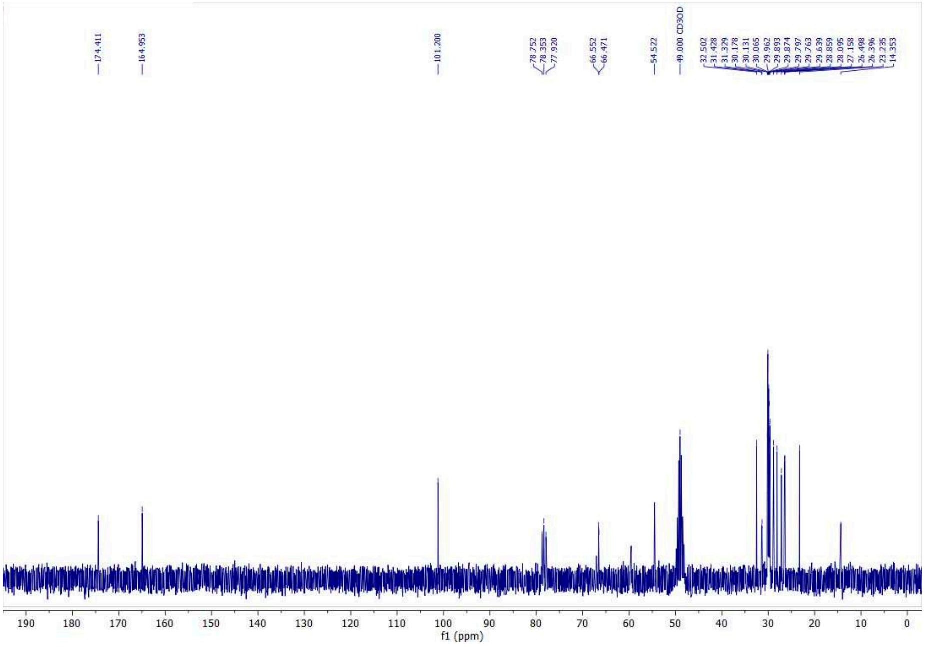

Figure S50. ^{13}C-NMR of compound **66** in CD_3OD at 150 MHz.

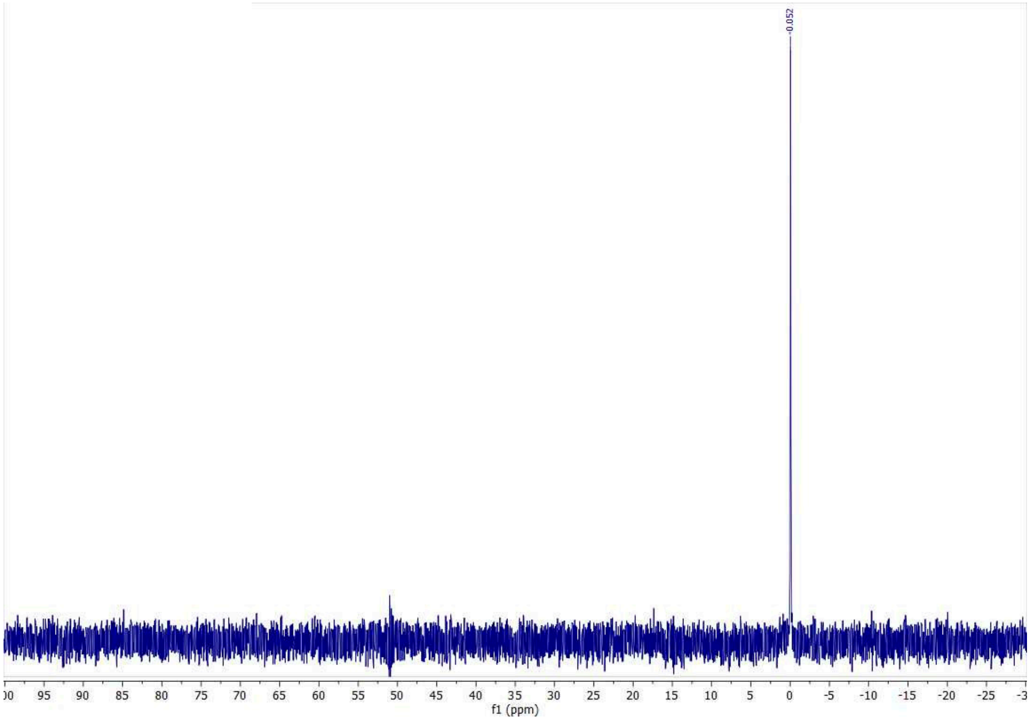

Figure S51. ^{31}P-NMR of compound **66** in CD_3OD at 121.44 MHz.
